# Proteolytic activation of executioner caspase-3 and -7 regulates different physiological processes in mice

**DOI:** 10.1038/s44318-026-00871-4

**Published:** 2026-07-21

**Authors:** Noëlle Sieg, Johanna Stachelscheid, Besarta Thaqi, Tanja Schwab, Fabian Schorn, Daniela Hahn, Adrian Georg Simon, Yuri Tolkach, Nima Abedpour, Simon E Tröder, Ingo Voigt, Felix Gaedke, Katja Wiegmann, Susanne Brodesser, Astrid Schauss, Branko Zevnik, Martin Krönke, Reinhard Büttner, Alexander Quaas, Michael Hallek, Melanie Fritsch, Hamid Kashkar

**Affiliations:** 1 University of Cologne, Faculty of Medicine and University Hospital of Cologne, Institute for Molecular Immunology, Cologne, Germany; 2 University of Cologne, Faculty of Medicine and University Hospital of Cologne, Department I of Internal Medicine, Cologne, Germany; 3 University of Cologne, Faculty of Medicine and University Hospital of Cologne, Institute of Pathology and Center for Integrated Oncology (CIO) Cologne Bonn, Cologne, Germany; 4 University of Cologne, Faculty of Medicine and University Hospital Cologne, Department of Translational Genomics, Cologne, Germany; 5 University of Cologne, Faculty of Medicine and University Hospital of Cologne, Center for Molecular Medicine Cologne (CMMC), Cologne, Germany; 6 Cancer Research Centre Cologne Essen, Faculty of Medicine and University Hospital Cologne, University of Cologne, Cologne, Germany; 7 University of Cologne, Faculty of Medicine and University Hospital of Cologne, Cluster of Excellence Cellular Stress Responses in Aging-associated Diseases (CECAD), Cologne, Germany; 8 In Vivo Research Facility, Faculty of Medicine and University Hospital Cologne, University of Cologne, Cologne, Germany; 9 Max Planck Institute for Biology of Ageing, Cologne, Germany; 10 University of Cologne, Faculty of Medicine and University Hospital of Cologne, Institute of Medical Microbiology, Immunology and Hygiene, Cologne, Germany; 11 Center for Integrated Oncology (CIO); Aachen Bonn Cologne Duesseldorf, Cologne, Germany

**Keywords:** Autophagy & Cell Death, Development

## Abstract

Caspase-3 (CASP3) and caspase-7 (CASP7) are the two major executioner caspases that are proteolytically activated by upstream initiator caspases. They possess almost indistinguishable activity, which has led to the overall view that these caspases have functionally redundant roles. Here, we generate knock-in mice expressing cleavage-resistant CASP3(D175A) or CASP7(D198A). Our results show that proteolytic activation of CASP3 and CASP7 is decisive for their activity in vivo and controls redundant processes during embryonic development as combined expression of both CASP3(D175A) and CASP7(D198A) causes embryonic lethality. In adult mice, however, activation of CASP3 and CASP7 controls different processes in different tissues, without the involvement of apoptosis. While CASP7 activation is required for male fertility by controlling spermatogenesis, CASP3 activation appears crucial for lymphoid tissue development by regulating interferon signalling. Our findings shed light on emerging roles of caspases in non-apoptotic processes and provide impetus for reconsidering their involvement in physiological and pathological conditions.

## Synopsis

Executioner caspase-3 (CASP3) and caspase-7 (CASP7) are commonly thought to have redundant biological functions. This study shows that in adult mice CASP7 activation is required for spermatogenesis and male fertility, whereas CASP3 activation blocks aberrant interferon production and inflammation.

- Activation of either CASP3 or CASP7 is sufficient for embryonic development.
- CASP3 is the major executioner caspase for apoptotic cell death.
- CASP7 activation is required for spermatogenesis and male fertility.
- CASP3 activity controls interferon production by cleaving cGAS and MAVS.
- Mice expressing cleavage-resistant CASP3(D175A) develop chronic perivascular inflammation resembling STING-associated inflammatory disorder.

## Abstract (web summary)

CASP3 activation is involved in interferon production, while CASP7 activity controls spermatogenesis in mice.

## Graphical abstract

**Figure:**
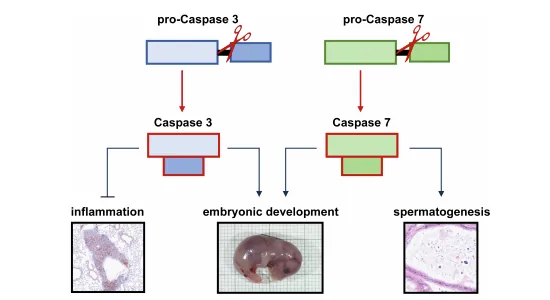

**Figure:**
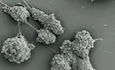

## Introduction

Caspases are cysteine-dependent aspartate-specific proteases (Alnemri et al, 1996) that elicit signalling events resulting in cellular death: apoptosis induced by pro-apoptotic caspases and pyroptosis induced by inflammatory caspases (Ramirez and Salvesen, 2018). Caspases are normally inactive zymogens consisting of an N-terminal domain followed by a C-terminal catalytic domain. The N-terminal domain is variable and receives the activation signals *via* interaction with upstream signalling platforms. The C-terminal part represents the catalytic unit which is a single domain but splits into two chains by proteolytic cleavage during maturation/activation, yielding a large and a small subunit. The catalytic dyad residues, Cys and His, reside in the large subunit, while the substrate recognition groove is formed primarily through residues within the small subunit (Ramirez and Salvesen, 2018; Salvesen and Dixit, 1997).

In vertebrates, the pro-apoptotic caspases can be further subdivided into initiator caspases (caspases-8, -9 and -10) and executioner caspases (caspases-3, -6 and -7) (Fuentes-Prior and Salvesen, 2004). The initiator caspases represent the point at which a cellular death signal is converted to proteolytic activity. This is achieved by remarkably coordinated clustering mechanisms that lead to initiator caspase dimerization followed by its conformational activation (Salvesen and Dixit, 1999). One of the major roles of the initiator caspases is to activate downstream executioner caspases, particularly caspases-3 and -7 (CASP3 and CASP7). The zymogens of executioner CASP3 and CASP7 are obligate dimers and their activity is held in check by a linker peptide, separating the large and small subunits. Extensive biochemical investigations indicate that the proteolytic processing of the linker peptide allows the assembly of the catalytic site and stabilizes the active enzyme conformation (Fuentes-Prior and Salvesen, 2004). Much of the biochemical and structural work on activation of the initiator caspases has focused on caspase-8 and -9 (CASP8 and CASP9), but the same concept holds true for the activation of the inflammatory caspase-1 (CASP1) (Ramirez and Salvesen, 2018). Furthermore, similar to initiator caspases, CASP1 can also activate executioner CASP3 and CASP7 by proteolytic processing of the linker peptide (Lamkanfi et al, 2008). Although apical caspases, including the initiator CASP8 and CASP9 and the inflammatory CASP1, respond to very different signals and are activated in distinct activation platforms, they all share the capability to cleave the linker peptide of executioner CASP3 (at D175) and CASP7 (at D198) (Fuentes-Prior and Salvesen, 2004). In contrast to executioner caspases, the cleavage of the linker peptide of initiator caspases does not seem to be required for their activation (Boatright et al, 2003). However, by expressing non-cleavable CASP8(D387A) (*Casp8*^*D387A/D387A*^) in mice, recent studies indicated that linker peptide cleavage of CASP8 is important for its proapoptotic activity and to prevent inflammation (Fritsch et al, 2019; Kang et al, 2008; Newton et al, 2019; Tummers et al, 2020).

In response to very different cell death stimuli, CASP3 and CASP7 are proteolytically activated by different apical caspases. Here, we investigated the physiological role of the cleavage of the linker peptide of executioner CASP3 and CASP7 in mice. Our data indicate that the proteolytic activation of CASP3 and CASP7 is decisive for their activation in vivo and controls redundant and non-redundant physiologic processes in mice, but not exclusively by involving apoptosis.

## Results

### Mice expressing cleavage-resistant CASP3(D175A) or CASP7(D198A) do not show obvious phenotypic alterations

**Figure EV1:**
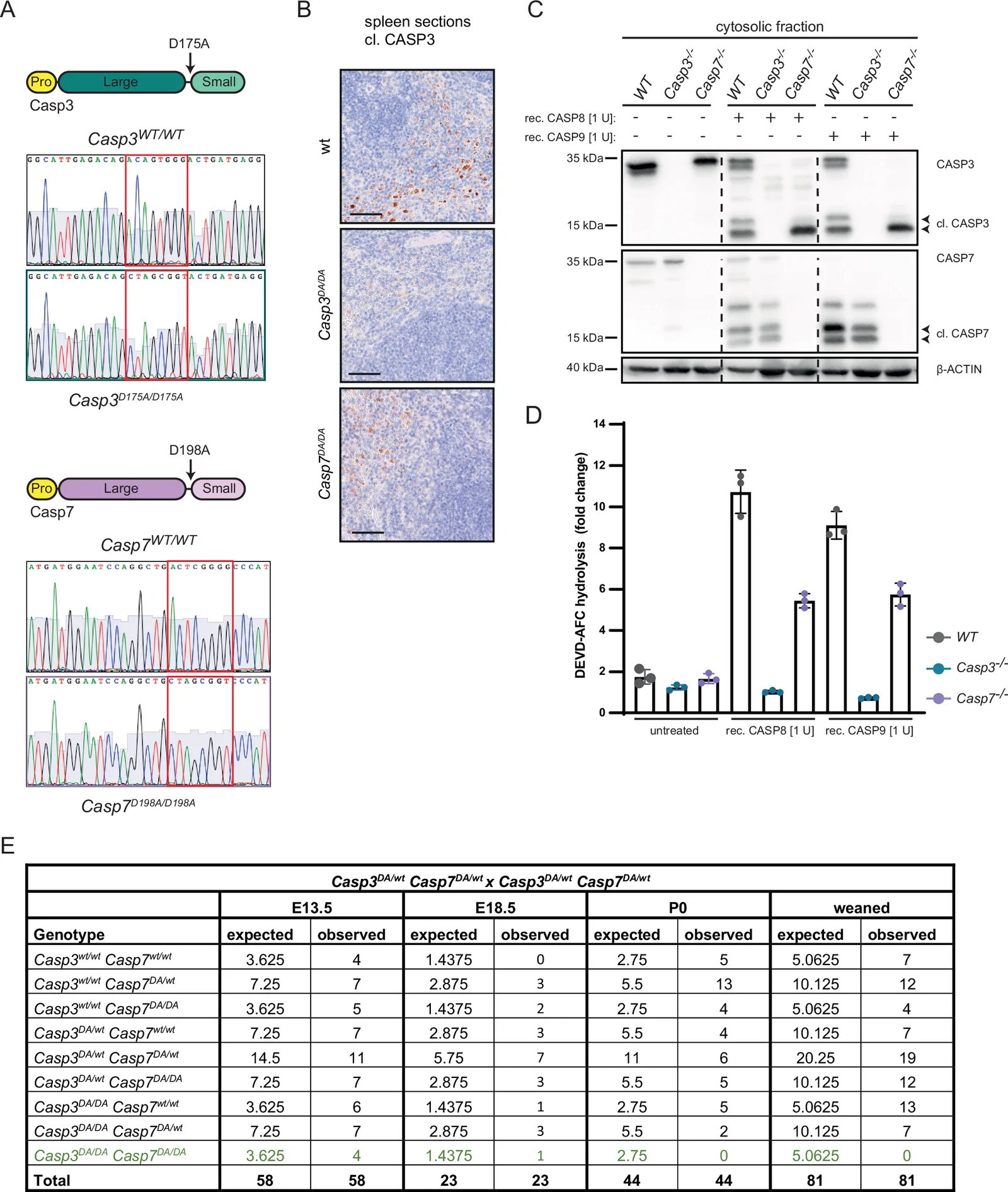
Mice expressing cleavage-resistant CASP3(D175A) or CASP7(D198A) do not show obvious phenotypic alterations. Related to Fig. 1. (**A**) Schematic illustration of the *Casp3* and *Casp7* genes with the domain structure and the position of the cleavage side (arrow) with representative sequence analysis of mice with the respective genotypes. (**B**) Representative spleen sections from 50-week-old *WT*, *Casp3*^*DA/DA*^, and *Casp7*^*DA/DA*^ mice immunostained for cleaved CASP3. Scale bars: 50 µm. (**C**) WB analysis of cytosolic extracts from *WT*, *Casp3*^−*/*−^, and *Casp7*^−*/*−^ MDFs treated with 1 U recombinant active CASP8 and CASP9. Lysates were analysed for cleavage of the indicated caspases. Results representative of three independent experiments. (**D**) DEVD-AFC hydrolysis measurement using cytosolic extracts (100 µg) from *WT*, *Casp3*^−*/*−^, and *Casp7*^−*/*−^ MDFs treated with 1 U recombinant active CASP8 or CASP9. Dots represent technical replicates of one experiment, representative of three independent experiments. Data is represented as mean ± s.d. (**E**) Expected and observed numbers of mice per genotype at E13.5, E18.5, day of birth (P0) and weaned, obtained from the indicated crossings. Source data are available online for this figure.

**Figure 1:**
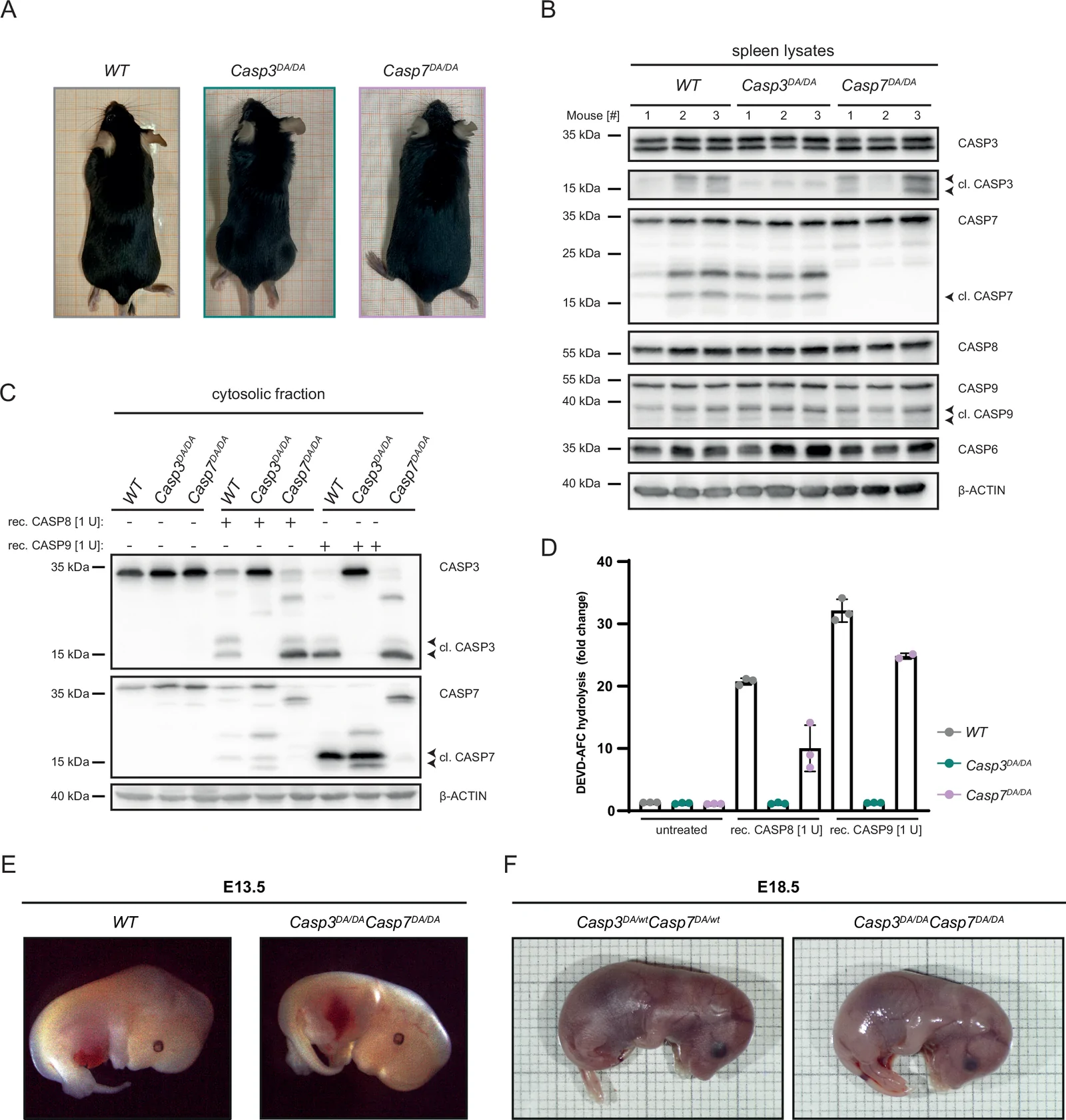
Mice expressing cleavage-resistant CASP3(D175A) or CASP7(D198A) do not show obvious phenotypic alterations. (**A**) Representative images of *WT*, *Casp3*^*DA/DA*^, and *Casp7*^*DA/DA*^ mice at 50 weeks of age. (**B**) Western blot (WB) analysis of spleen lysates from *WT* (*n* = 3), *Casp3*^*DA/DA*^ (*n* = 3), and *Casp7*^*DA/DA*^ (*n* = 3) mice at 30 weeks of age. Lanes represent individual mice. Lysates were analysed for the expression of the indicated proteins. (**C**) WB analysis of cytosolic extracts from *WT*, *Casp3*^*DA/DA*^, and *Casp7*^*DA/DA*^ MEFs treated with 1 U recombinant active CASP8 or CASP9. Lysates were analysed for cleavage of the indicated caspases. Results are representative of at least three independent experiments. (**D**) DEVD-AFC hydrolysis measurement using cytosolic extracts (100 µg) from *WT*, *Casp3*^*DA/DA*^, and *Casp7*^*DA/DA*^ MEFs treated with 1 U recombinant active CASP8 or CASP9. Dots represent technical replicates, representative of three independent experiments. Data is represented as mean ± s.d. (**E**) Representative images of *WT* and *Casp3*^*DA/DA*^*Casp7*^*DA/DA*^ embryos at E13.5. (**F**) Images of *Casp3*^*DA/wt*^*Casp7*^*DA/wt*^ and *Casp3*^*DA/DA*^*Casp7*^*DA/DA*^ embryos at E18.5. Source data are available online for this figure.

In order to investigate the physiological role of the linker peptide cleavage in executioner caspases, we generated mice with point mutations in the endogenous loci that express cleavage-resistant CASP3(D175A) or CASP7(D198A) by mutating the responsible aspartate residues in the linker peptides (Fig. EV1A). Sanger sequencing of the founder population identified animals harbouring the specific knock-in mutation. Founder mice (*Casp3*^*D175A/WT*^ and *Casp7*^*D198A/WT*^) were backcrossed to C57BL/6 N wild-type mice to eliminate possible CRISPR/Cas9-mediated genetic mosaicism. Interbreeding of heterozygous mice produced *Casp3*^*D175A/D175A*^ (*Casp3*^*DA/DA*^) and *Casp7*^*D198A/D198A*^ (*Casp7*^*DA/DA*^) mice that were born at normal Mendelian frequencies and did not show obvious phenotypic alteration (Fig. 1A). Western blot (WB) analysis of the respective spleen lysates (3 mice per genotype) showed that both cleavage-resistant CASP3(D175A) and CASP7(D198A) variants were expressed. Cleaved CASP3 (cl. CASP3) was detected in wild-type (WT) and *Casp7*^*DA/DA*^ mice, but not in spleen sections or lysates derived from *Casp3*^*DA/DA*^ mice (Figs. 1B and EV1B). Similarly, cleaved CASP7 (cl. CASP7) was only detected in *WT* and *Casp3*^*DA/DA*^ mice, but not in spleens of *Casp7*^*DA/DA*^ mice (Fig. 1B). These observations indicated that mutation of the cleavage site aspartate successfully blocks executioner caspase processing in mice.

Next, we examined whether CASP3(D175A) and CASP7(D198A) variants can be proteolytically activated by initiator caspases. Therefore, we isolated cytosolic extracts from mouse embryonic fibroblasts (MEFs) and treated them with recombinant active CASP8 (rec. CASP8) or CASP9 (rec. CASP9) (Andree et al, 2014; Gunther et al, 2020; Kashkar et al, 2003). WB analysis showed that in contrast to *WT* cells, proteolytic processing of CASP3 and CASP7, yielding the active p20/17 fragments, was efficiently blocked in cytosolic extracts derived from cells expressing cleavage-resistant CASP3(D175A) or CASP7(D198A), respectively (Fig. 1C). A 33 kDa fragment of CASP7 was, however, detected in the cytosolic extracts from *Casp7*^*DA/DA*^ cells after incubation with rec. CASP8 or rec. CASP9, likely as a result of an additional proteolytic processing of CASP7 either by initiator caspases or active CASP3. Measurement of executioner caspase activity using DEVD-AFC revealed a complete lack of DEVDase activity in the cytosolic extracts of *Casp3*^*DA/DA*^ cells (Fig. 1D). *Casp7*^*DA/DA*^ cells showed only reduced DEVDase activity indicating that CASP3 is the main executioner caspase responsible for the DEVD-AFC hydrolysis. The major role of CASP3 in DEVD-AFC hydrolysis was also confirmed in cytosolic extracts isolated from mouse dermal fibroblast (MDFs) lacking either *Casp3* (*Casp3*^*−/−*^) or *Casp7* (*Casp7*^*−/−*^) (Saavedra et al, 2018) (Fig. EV1C,D).

During embryogenesis, apoptosis is considered to counteract cell proliferation and to ensure tissue development (Voss and Strasser, 2020). Yet, both *Casp3*^*DA/DA*^ and *Casp7*^*DA/DA*^ mice developed normally suggesting that CASP3 and CASP7 provide redundant functions during embryonic development. In order to examine this notion, we sought to establish *Casp3*^*DA/DA*^*Casp7*^*DA/DA*^ mice. Intercrossing heterozygous *Casp3*^*DA/WT*^*Casp7*^*DA/WT*^ mice, however, failed to produce progeny simultaneously carrying homozygous CASP3 and CASP7 cleavage site mutations, as we did not observe any *Casp3*^*DA/DA*^*Casp7*^*DA/DA*^ mice at early postnatal P0 (Fig. EV1E). *Casp3*^*DA/DA*^*Casp7*^*DA/DA*^ embryos were present at the expected Mendelian frequencies at embryonic day 13.5 (E13.5) and E18.5 (Figs. 1E,F and EV1E), indicating that the lack of proteolytic activation of CASP3 and CASP7 likely interferes with the prenatal development of mice. Importantly, lack of CASP8 or its enzymatic activity leads to embryonic lethality in mice at around E11.5 and is associated with hyperaemia in the abdominal areas of the embryos, owing to defects in vascular development (Fritsch et al, 2019; Varfolomeev et al, 1998). CASP9 deficiency in mice leads to perinatal lethality which is associated with defects in neuronal tube closure, presented already at E10.5 and progresses to an expansion of the midbrain region underneath the foetal skin tissue at E13.5 (Kuida et al, 1998). None of these alterations could be observed in *Casp3*^*DA/DA*^*Casp7*^*DA/DA*^ embryos at E13.5-E18.5 (Fig. 1E,F), yet they died shortly before or after birth (Fig. EV1E). These observations are in line with a previous report analysing *Casp3*^*−/−*^*Casp7*^*−/−*^ double-knockout mice (DKO) (Lakhani et al, 2006). *Casp3*^*−/−*^*Casp7*^*−/−*^ mice were present at normal Mendelian numbers at E20, but died rapidly after birth suggesting that, unlike CASP9, neither CASP3 nor CASP7 is important for neuronal development. Independently, our data indicated that CASP3 and CASP7 provide redundant functions during embryonic development. Whether apoptosis represents the decisive process to be controlled by active CASP3 and CASP7 during embryogenesis requires further in-depth investigations by also involving *Casp9*- and *Casp8*-deficient mice.

**Figure EV2:**
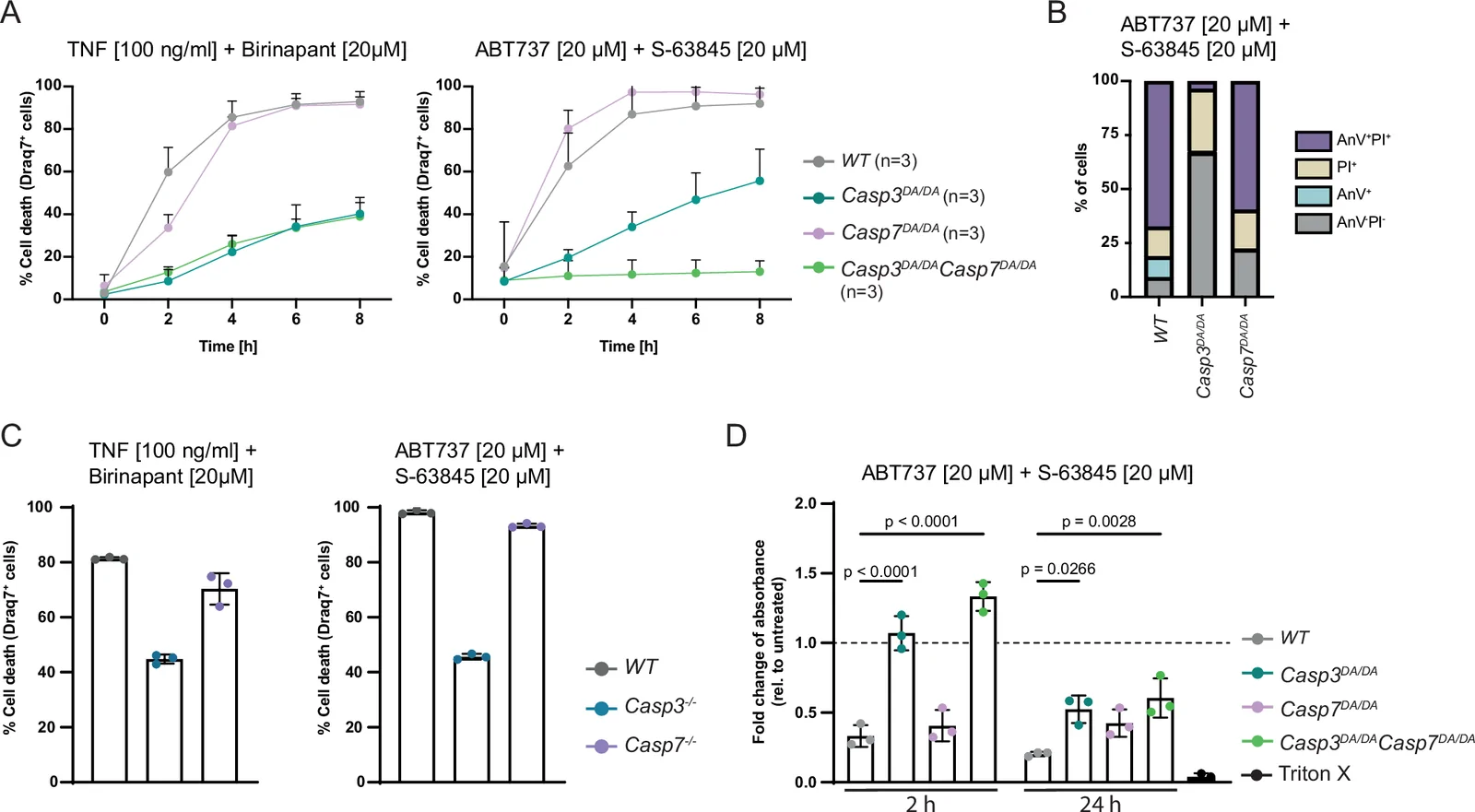
CASP3 is the major driver of apoptosis. Related to Fig. 2. (**A**) IncuCyte analysis to determine % cell death of MEFs with the indicated genotypes treated with TNF (100 ng/ml) and birinapant (20 µM) or S-63845 (20 µM) and ABT737 (20 µM) using DRAQ7 dye for the indicated time points. Birinapant was added 1 h prior to treatment. Data are displayed as mean of three independent experiments ± s.d. (**B**) Flow cytometry analysis of MEFs from *WT*, *Casp3*^*DA/DA*^, and *Casp7*^*DA/DA*^ mice stained with Annexin V and PI after 1 h treatment with ABT737 and S-63845 in the indicated concentrations. (**C**) IncuCyte analysis to determine % cell death of MDFs using DRAQ7 dye with the indicated genotypes treated with TNF (100 ng/ml) and birinapant (20 µM) or S-63845 (20 µM) and ABT737 (20 µM) for 4 h. Birinapant was added 1 h prior to treatment. Data is displayed as mean of three technical replicates ± s.d. Results representative of three independent experiments. (**D**) Measurement of metabolic activity by CellTiter 96® AQueous One Solution (MTS) of *WT*, *Casp3*^*DA/DA*^, and *Casp7*^*DA/DA*^ MEFs, treated with S-63845 (20 µM) and ABT737 (20 µM) for 2 h and 24 h. Fold change of absorbance at 490 nm relative to untreated cells of the same genotype. Dots represent independent experiments (*n* = 3). Cells treated with 0.1% Triton X were used as negative control. Data is represented as mean ± s.d. One-way ANOVA followed by Dunnett’s post-analysis compared to the corresponding *WT* values. Exact *P* values: 0.000000943 (*WT* vs *Casp3*^*DA/DA*^) and 0.000000008 (*WT* vs *Casp3*^*DA/DA*^ *Casp7*^*DA/DA*^).

In line with the data obtained in our cell-free studies, analysis of cell death revealed CASP3 as the major driver of apoptosis in response to extrinsic and intrinsic pro-apoptotic stimuli. Reduced cell death was observed in *Casp3*^*DA/DA*^ MEFs when the cells were exposed to TNF/birinapant involving the initiator CASP8, or to BH3 mimetics (ABT737/S-63845) involving the initiator CASP9 for up to 8 h (Fig. EV2A,B). Cells expressing CASP7(D198A) did not show any alteration of cell death in response to extrinsic or intrinsic apoptotic stimuli. Similar results were obtained using *Casp3*^*−/−*^ and *Casp7*^*−/−*^ cells (Fig. EV2C).

**Figure 2:**
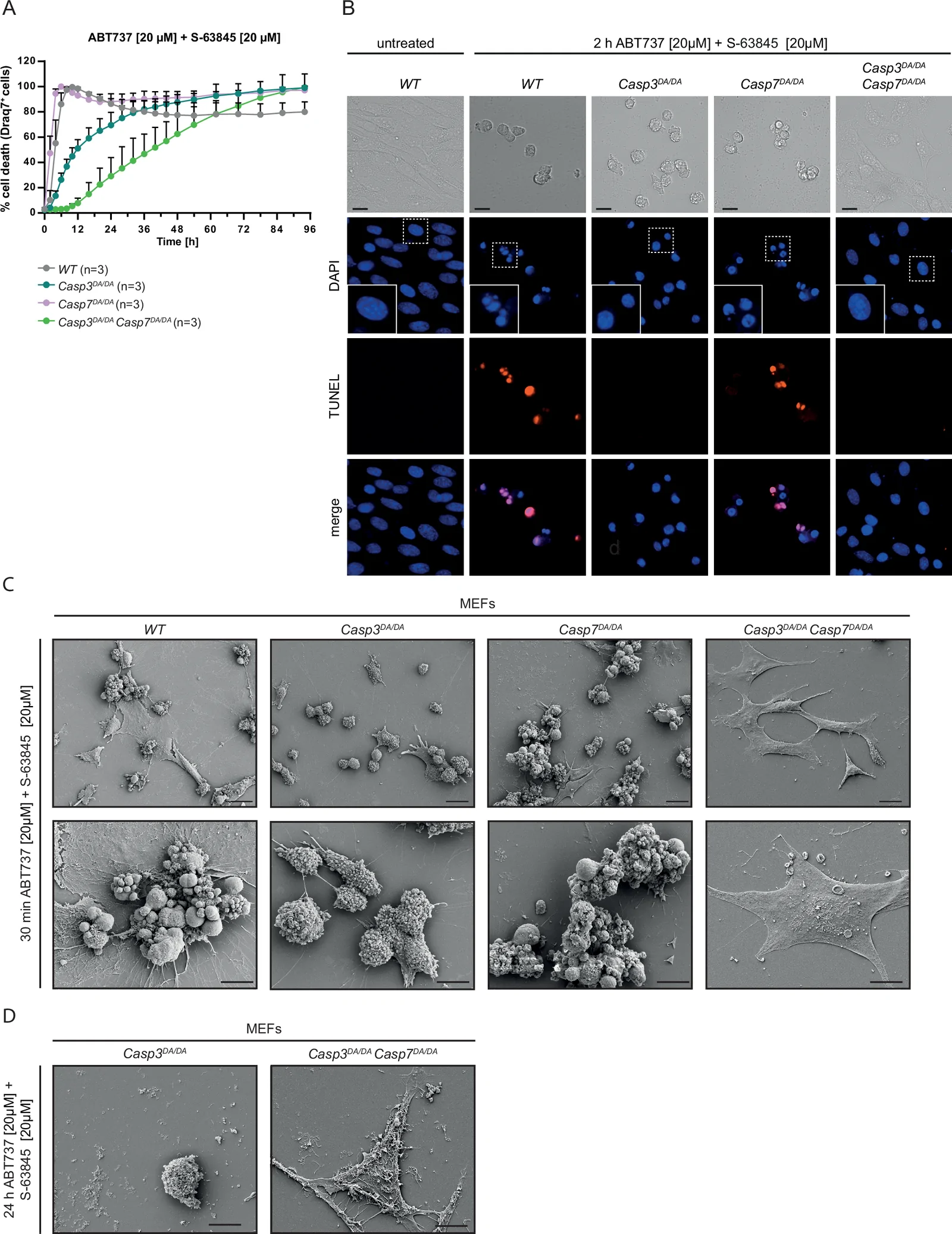
CASP3 is the major driver of apoptosis. (**A**) IncuCyte analysis to determine % cell death of MEFs with the indicated genotypes using DRAQ7 dye and treated with S-63845 (20 µM) and ABT737 (20 µM) for the indicated time points. Data are displayed as mean of three independent experiments ± s.d. (**B**) TUNEL and DAPI staining of *WT*, *Casp3*^*DA/DA*^, *Casp7*^*DA/DA*^, and *Casp3*^*DA/DA*^*Casp7*^*DA/DA*^ MEFs treated with S-63845 (20 µM) and ABT737 (20 µM) for 2 h. Scale bars = 20 µm. (**C**) SEM images of *WT*, *Casp3*^*DA/DA*^, *Casp7*^*DA/DA*^, and *Casp3*^*DA/DA*^*Casp7*^*DA/DA*^ MEFs treated with S-63845 (20 µM) and ABT737 (20 µM) for 30 min. Scale bars: 20 µm (top) and 10 µm (bottom). (**D**) SEM images of *Casp3*^*DA/DA*^ and *Casp3*^*DA/DA*^*Casp7*^*DA/DA*^ MEFs treated with S-63845 (20 µM) and ABT737 (20 µM) for 24 h. Scale bars: 10 µm. Source data are available online for this figure.

Analysis of cell death in MEFs isolated from *Casp3*^*DA/DA*^*Casp7*^*DA/DA*^ embryos showed that the concomitant expression of CASP7(D198A) barely interfered with TNF/birinapant-induced cytotoxicity. However, combined expression of CASP3(D175A) and CASP7(D198A) almost completely blocked BH3 mimetics-induced cell death at early time points up to 8 h post-treatment (Fig. EV2A). However, at later time points, BH3 mimetics were still able to efficiently induce cell death in both *Casp3*^*DA/DA*^ (24 h post-treatment) and *Casp3*^*DA/DA*^*Casp7*^*DA/DA*^ (60 h post-treatment) MEFs (Fig. 2A), likely due to the toxicity of BH3 mimetics by inducing mitochondrial membrane damage and non-apoptotic cell death, independent of caspase activity (Tait and Green, 2008). Microscopic investigations showed membrane blebbing, nuclear fragmentation and TUNEL positivity (DNA fragmentation) only in *WT* and *Casp7*^*DA/DA*^ cells expressing functional CASP3 (Fig. 2B). These observations suggested that CASP3 serves as the major determinant of apoptosis which was further confirmed by detailed scanning electron microscopy (SEM) (Fig. 2C,D). SEM microscopy revealed specific characteristics of apoptotic cell death only in cells expressing wild-type CASP3 (Fig. 2C). No membrane blebbing or cellular fragmentation was observed in *Casp3*^*DA/DA*^*Casp7*^*DA/DA*^ MEFs. However, distinct membrane blebbing leading to cellular shrinkage without progressing to nuclear fragmentation was visible when only wild-type CASP7 was expressed in *Casp3*^*DA/DA*^ MEFs (Fig. 2B,C). These observations indicate that CASP7 is less effective in promoting features of the apoptotic phenotype likely by failing to induce nuclear fragmentation and complete apoptotic demise of cells. Importantly, microscopic examination of *Casp3*^*DA/DA*^*Casp7*^*DA/DA*^ MEFs after 24 h post-treatment did not show cellular damage (Fig. 2D). Cell viability assays based on metabolic activity of cells, however, revealed marked reduction in cellular viability upon BH3 mimetics treatment of *Casp3*^*DA/DA*^*Casp7*^*DA/DA*^ MEFs (Fig. EV2D). These data collectively indicate that CASP3 is mainly responsible for the apoptotic death in response to BH3 mimetics. Lack of executioner caspase activation, in particular, activation of CASP3 results in delayed kinetics of cell death but does not completely diminish the cytotoxic activity of BH3 mimetics.

**Figure 3:**
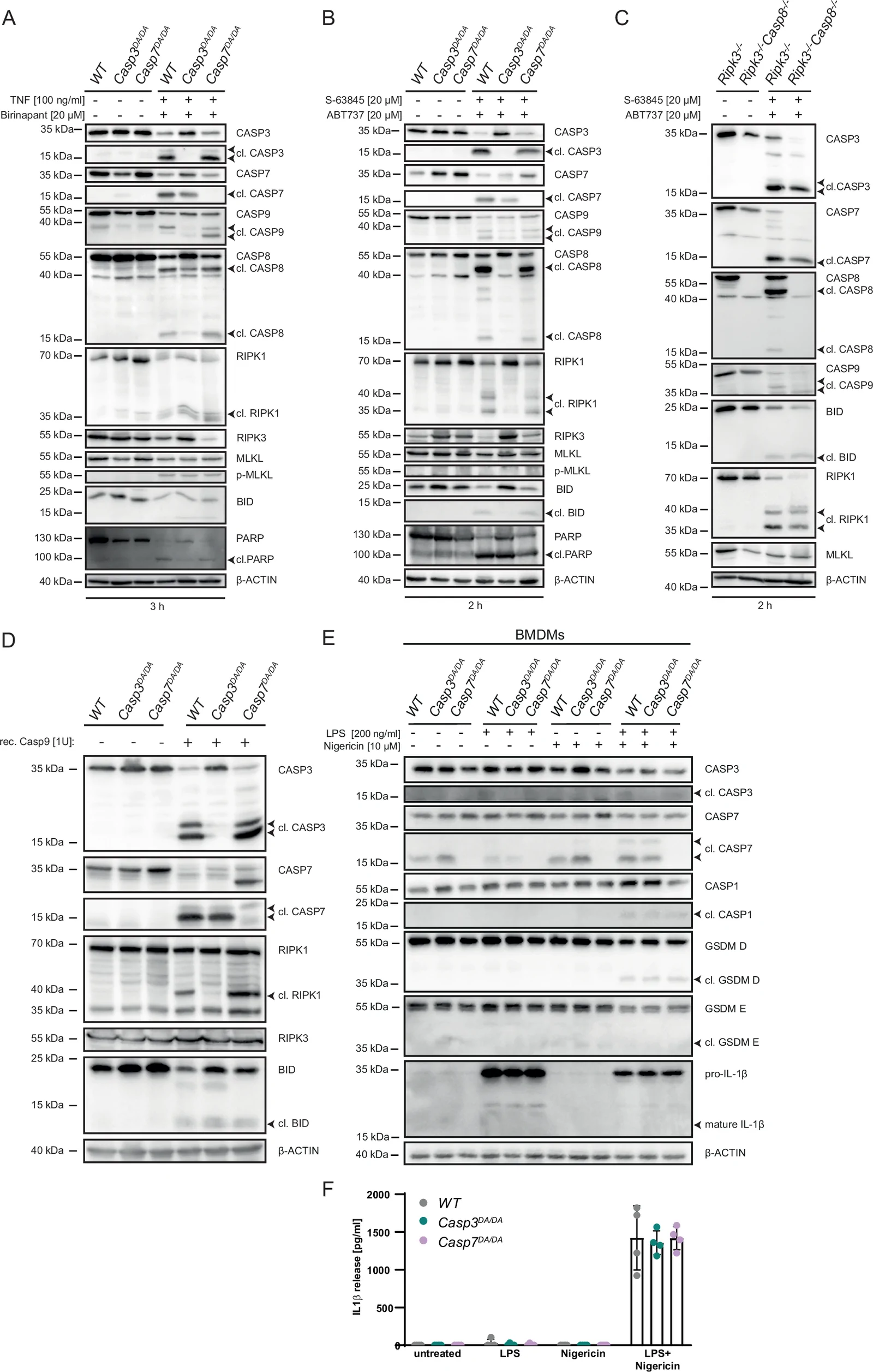
Activation of CASP3 is important for proteolytic processing of the majority of apoptotic substrates. (**A**) WB analysis of MEFs with the indicated genotypes treated with TNF (100 ng/ml) and birinapant (20 µM) for 3 h. Birinapant was added 1 h prior to treatment. Results are representative of three independent experiments. (**B**) WB analysis of MEFs with the indicated genotypes treated with S-63845 (20 µM) and ABT737 (20 µM) for 2 h. Results are representative of three independent experiments. (**C**) WB analysis of *Ripk3*^−/−^ and *Ripk3*^−*/*−^*Casp8*^−*/*−^ MDFs treated with S-63845 (20 µM) and ABT737 (20 µM) for 2 h. (**D**) WB analysis of cytosolic extracts from *WT*, *Casp3*^*DA/DA*^, and *Casp7*^*DA/DA*^ MEFs treated with 1 U recombinant active CASP9. Lysates were analysed for cleavage of the indicated proteins. Results are representative of three independent experiments. (**E**) WB analysis of BMDMs with the indicated genotypes treated with LPS (200 ng/ml) and nigericin (10 µM) for 2 h. LPS was added 4 h prior to treatment. Results representative of three independent experiments. (**F**) IL-1β release measured by ELISA in cell culture supernatant of BMDMs from *WT* (*n* = 3), *Casp3*^*DA/DA*^ (*n* = 3), and *Casp7*^*DA/DA*^ (*n* = 3) mice as treated in (**E**). Dots represent biological replicates. Data are represented as mean ± s.d. Source data are available online for this figure.

**Figure EV3:**
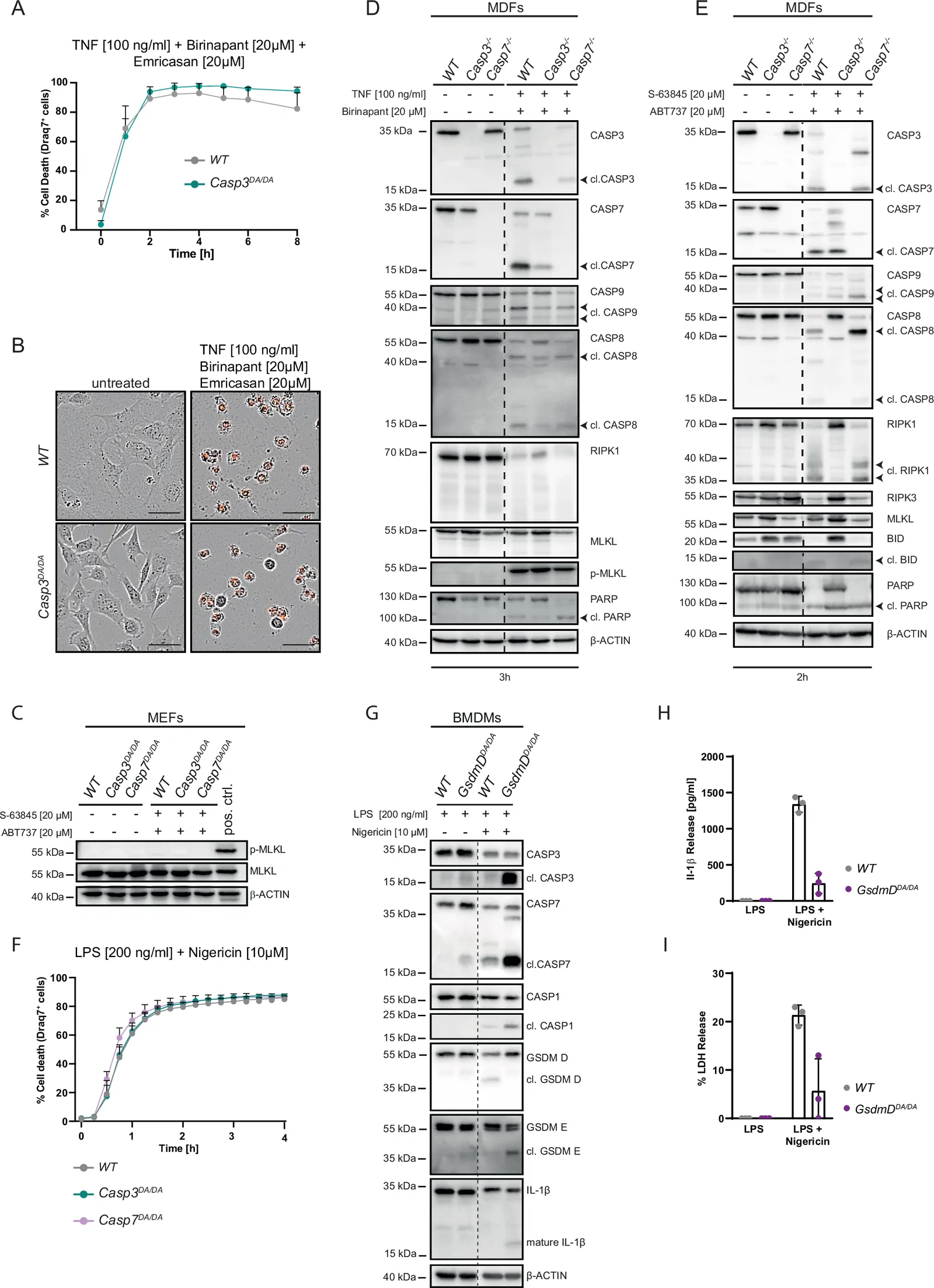
Activation of CASP3 is important for proteolytic processing of the majority of apoptotic substrates. Related to Fig. 3. (**A**) IncuCyte analysis to determine % cell death of MEFs using DRAQ7 dye with the indicated genotypes treated with TNF (100 ng/ml), birinapant (20 µM), and emricasan (20 µM) at the indicated time points. Birinapant was added 1 h prior to treatment. Data is displayed as mean of three technical replicates ± s.d. Results representative of three independent experiments. (**B**) Representative images of MEFs with the indicated genotype treated with TNF (100 ng/ml), birinapant (20 µM), and emricasan (20 µM) for 1 h obtained by IncuCyte. Results representative of three independent experiments. Scale bars: 100 µm. (**C**) WB analysis of MLKL phosphorylation of lysates in Fig. 3B including a positive control: *WT* MEFs treated with TNF (100 ng/ml), birinapant (20 µM), and emricasan (20 µM). (**D**) WB analysis of MDFs with the indicated genotypes treated with TNF (100 ng/ml) and birinapant (20 µM) for 3 h. Birinapant was added 1 h prior to treatment. Lysates were analysed for the expression of the indicated proteins. Results representative of three independent experiments. (**E**) WB analysis of MDFs treated with S-63845 (20 µM) and ABT737 (20 µM) for 2 h. Lysates were analysed for the expression of the indicated proteins. Results representative of three independent experiments. (**F**) IncuCyte analysis to determine % cell death of bone marrow derived macrophages (BMDMs) using DRAQ7 dye with the indicated genotypes treated with LPS (200 ng/ml) and nigericin (10 µM) at the indicated time points. LPS was added 4 h prior to treatment. Data is displayed as mean of three technical replicates ± s.d. (**G**) WB analysis of *WT* BMDMs and BMDMs expressing cleavage-resistant Gasdermin D(D276A) treated with LPS (200 ng/ml) alone or together with nigericin (10 µM) for 2 h. LPS was added 4 h prior to treatment. Lysates were analysed for the expression of the indicated proteins. Results representative of three independent experiments. (**H**) IL-1β release measured by ELISA in cell culture supernatant of BMDMs from *WT* (*n* = 3) and *GsdmD*^*DA/DA*^ (*n* = 3) mice as treated in (**G**). Dots represent biological replicates. Data are represented as mean ± s.d. (**I**) LDH release of BMDMs in cell culture supernatant of BMDMs from *WT* (*n* = 3) and *GsdmD*^*DA/DA*^ (*n* = 3) mice as treated in (**G**). Dots represent technical replicates. Data are represented as mean ± s.d. Source data are available online for this figure.

In line with our cell death analyses, WB examination of PARP cleavage as a sign of apoptotic death in response to TNF/birinapant further confirmed CASP3 as the main driver of apoptosis (Fig. 3A). Lack of CASP3 activation was also associated with reduced processing of CASP8 and CASP9, in line with previous studies showing that active CASP3 can proteolytically process initiator caspases (Slee et al, 1999). Independently, activation of the extrinsic apoptotic pathway has been recently shown to induce CASP3-mediated cleavage of RIPK3 (Newton et al, 2024). Consistently, TNF/birinapant treatment reduced the amount of RIPK3 in *WT* cells, which was completely restored in *Casp3*^*DA/DA*^ cells lacking CASP3 activity (Fig. 3A). Although full-length RIPK3 levels were higher in *Casp3*^*DA/DA*^ cells following TNF/birinapant treatment, MLKL phosphorylation appeared unaltered, indicating that inactivation of CASP3 unlikely interferes with necroptosis in response to TNF/birinapant. This notion was further confirmed by exposing cells to TNF/birinapant/emricasan inducing necroptosis (Fig. EV3A,B). Furthermore, TNF/birinapant treatment induced the cleavage of BID and RIPK1 (cl. RIPK1) which was independent of CASP3 proteolytic activation. In contrast to *Casp3*^*DA/DA*^ cells, the TNF/birinapant-induced apoptotic cascade was not altered in *Casp7*^*DA/DA*^ cells. Collectively, these observations indicated that CASP3 processing and activation is the decisive step in the execution of extrinsic apoptosis.

Similar to extrinsic apoptosis, PARP cleavage induced by the BH3 mimetics ABT737/S-63845 was reduced only in *Casp3*^*DA/DA*^ cells (Fig. 3B). Failure of CASP3 activation was also associated with reduced CASP9 processing and lack of CASP8 processing in response to ABT737/S-63845 as previously described (Slee et al, 1999). Similar to extrinsic apoptosis, the activation of the intrinsic apoptotic pathway reduced the level of RIPK3, which was restored in *Casp3*^*DA/DA*^ cells. Furthermore, ABT737/S-63845 treatment also led to the cleavage of RIPK1, yielding two RIPK1 fragments, p37 and p35, which was completely blocked in *Casp3*^*DA/DA*^ cells. RIPK1 processing in response to ABT737/S-63845 treatment was CASP8-independent, as the respective cleavage products were still detectable in *Casp8*^*−/−*^*Ripk3*^*−/−*^ cells (Fig. 3C) suggesting that CASP9-mediated CASP3 activation leads to proteolytic processing of RIPK1. Accordingly, we could detect RIPK1 cleavage in the cytosolic extracts of cells treated with rec. CASP9 in *WT* and *Casp7*^*DA/DA*^, but not in *Casp3*^*DA/DA*^ cells (Fig. 3D). These observations indicated that the intrinsic apoptotic pathway interferes with necroptotic signalling by inducing CASP3-dependent cleavage of RIPK1 and RIPK3. However, ABT737/S-63845 treatment did not lead to necroptosis, as phosphorylated MLKL was neither detected in *WT* nor in *Casp3*^*DA/DA*^ cells (Figs. 3B and EV3C). Finally, ABT737/S-63845 treatment also led to BID cleavage in *WT* and *Casp7*^*DA/DA*^ cells, but not in *Casp3*^*DA/DA*^ cells (Fig. 3B). Thus, activation of CASP3 downstream of mitochondria induces BID processing, which might amplify mitochondrial apoptosis as shown in two independent previous studies (Lakhani et al, 2006; Slee et al, 2000). BID cleavage in response to ABT737/S-63845 treatment was independent of CASP8, as it was also detectable in *Casp8*^*−/−*^*Ripk3*^*−/−*^ cells after ABT737/S-63845 treatment (Fig. 3C). The more central role of CASP3 in executing cell death, particularly the cleavage of CASP8, CASP9, RIPK3, RIPK1, and BID was further supported in our studies using *Casp3*^*−/−*^ and *Casp7*^*−/−*^ cells (Fig. EV3D,E).

Similar to initiator CASP8 and CASP9, CASP1 has also been shown to process CASP3 and CASP7 during the course of macrophage-mediated anti-bacterial immunity (de Vasconcelos et al, 2020; Lamkanfi et al, 2008). In order to examine the role of executioner caspase processing in pyroptosis, we isolated bone marrow-derived macrophages (BMDMs) from mice and exposed them to LPS/nigericin which activates the NLRP3 inflammasome, subsequently inducing GSDMD-dependent pyroptosis and IL-1β secretion (de Vasconcelos et al, 2020; Simpson et al, 2022). Although both CASP3 and CASP7 were activated in response to LPS/nigericin treatment, neither cell death nor IL-1β secretion was affected in BMDMs isolated from *Casp3*^*DA/DA*^ and *Casp7*^*DA/DA*^ mice (Figs. 3E,F and EV3F). These results indicated that the proteolytic activation of executioner caspases is unlikely decisive for the execution of pyroptosis in response to LPS/nigericin 2 h post-treatment, but does not generally exclude the possible involvement of executioner caspases in pyroptosis under other experimental conditions. Importantly, activation of CASP3/7 can lead to the processing/activation of GSDME and lytic cell death (Wang et al, 2017). However, we could not detect any GSDME cleavage in BMDMs upon LPS/nigericin likely due to rapid GSDMD-mediated pyroptosis (Fig. 3E). Accordingly, GSDME cleavage could be detected in response to LPS/nigericin, only when GSDMD cleavage was blocked in BMDMs expressing cleavage-resistant GSDMD(D276) (Theobald et al, 2021) (Fig. EV3G). Upon inhibition of GSDMD-mediated pyroptosis, both LDH release and IL-1β secretion were grossly diminished (Fig. EV3H,I). Activated CASP3 and CASP7 were accumulated in *Gsdmd*^*DA/DA*^ BMDMs leading to processing of GSDME (Fig. EV3G). Intriguingly, mature IL-1β was detected in cellular lysates but not in the supernatants of *Gsdmd*^*DA/DA*^ BMDMs after LPS/nigericin treatment (Fig. EV3G,H) indicating that CASP3/7 activation and, in particular, GSDME-mediated pores are less efficient for pyroptosis and IL-1β release in BMDMs after 2 h LPS/nigericin treatment.

In summary, our data showed that the mutation of the linker peptides of CASP3 at D175 or CASP7 at D198 efficiently diminishes the capability of initiator caspases to induce executioner caspase activity. After activation, CASP3 and CASP7 appeared to target different cellular substrates and varied in their potency to induce apoptosis. However, they acted redundantly during embryonic development.

### Casp7^DA/DA^ male mice are infertile and show altered spermatogenesis

**Figure EV4:**
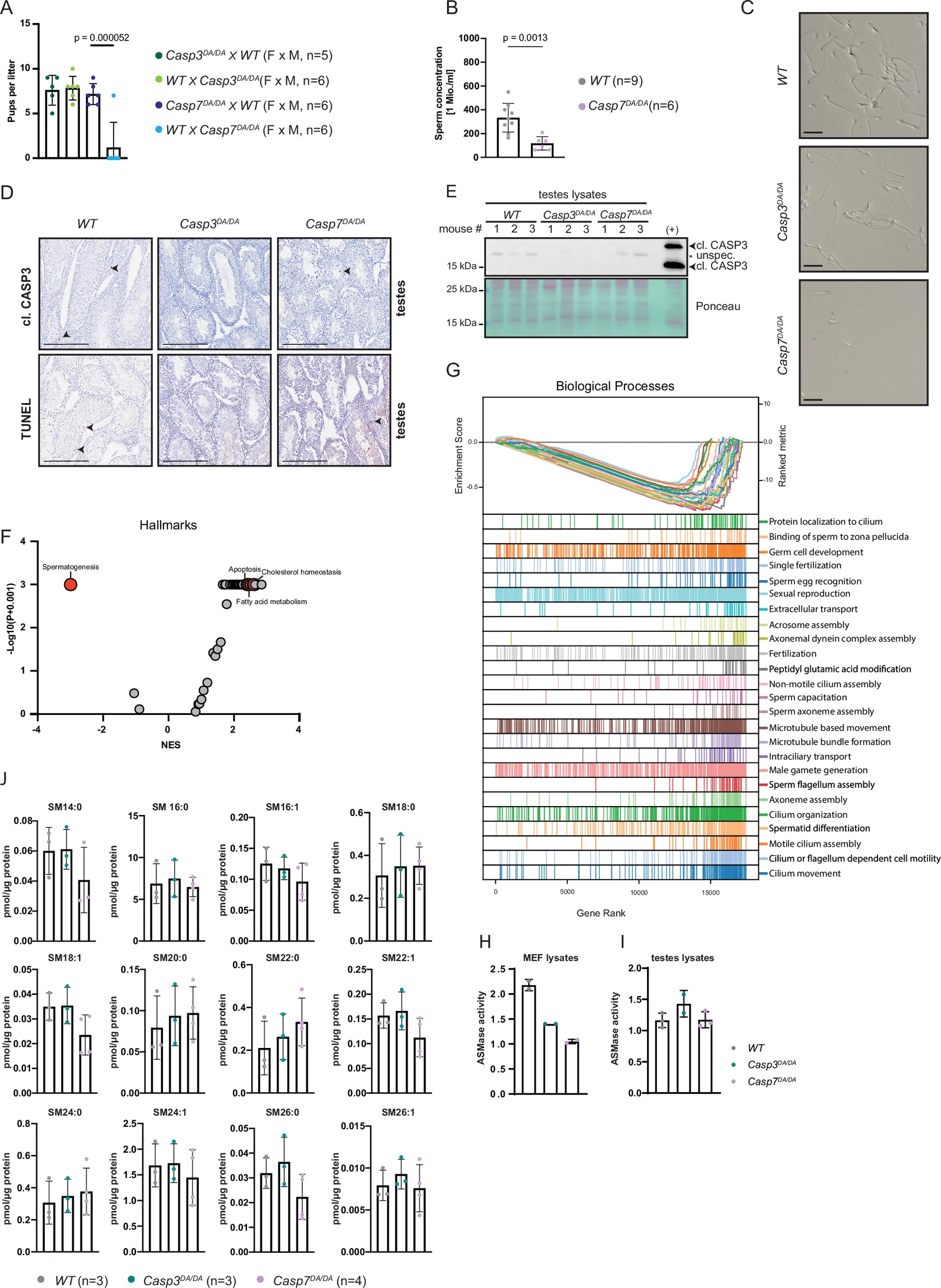
*Casp7*^*DA/DA*^ male mice are infertile and show altered spermatogenesis. Related to Fig. 4. (**A**) Observed pups per litter obtained from the indicated crossings. Data are mean ± s.d. One-way ANOVA with Sidak’s post-analysis test. (**B**) Quantification of sperm concentration [1 Mio/ml] in *WT* (*n* = 9) and *Casp7*^*DA/DA*^ (*n* = 6) mice at 12–30 weeks of age, measured using a Neubauer chamber. Data are mean ± s.d. Unpaired *t* test was performed. (**C**) Representative images of sperm from *WT*, *Casp3*^*DA/DA*^, and *Casp7*^*DA/DA*^ mice at 12–30 weeks of age. Scale bars = 10 µm. (**D**) Representative images of testes of *WT*, *Casp3*^*DA/DA*^, and *Casp7*^*DA/DA*^ mice at 30 weeks of age immunostained for cl. CASP3 and stained for DNA fragmentation by TUNEL assay. Scale bars: 0.25 mm. Arrows indicate positively stained cells. (**E**) Control WB for testes lysates from Fig. 4F, showing specific bands for cl. CASP3 in MEFs treated with S-63845 (20 µM) and ABT737 (20 µM) for 2 h (+). Asterix indicates unspecific band. (**F**) NES derived from GSEA comparing testes from *WT* (*n* = 3) and *Casp7*^*DA/DA*^ mice (*n* = 3), using the hallmark gene set database. Significance was tested using default settings of the gseapy Python package (version 1.1.0), based on random permutation. (**G**) Enrichment plots of pathways (biological processes) involved in the male reproductive system, significantly downregulated in *Casp7*^*DA/DA*^ (*n* = 3) vs *WT* mice (*n* = 3). (**H**) ASMase activity, measured in lysates from *WT*, *Casp3*^*DA/DA*^, and *Casp7*^*DA/DA*^ MEFs. Dots represent independent experiments. Data are mean ± s.d. (**I**) ASMase activity, measured in lysates from *WT* (*n* = 3), *Casp3*^*DA/DA*^ (*n* = 2), and *Casp7*^*DA/DA*^ (*n* = 3) testes. Dots represent individual mice. Data are mean ± s.d. (**J**) Concentration of different sphingomyelins [pmol/µg protein] in testes from *WT* (*n* = 3), *Casp3*^*DA/DA*^ (*n* = 3), and *Casp7*^*DA/DA*^ (*n* = 4) mice at 30 weeks of age. Data are mean ± s.d. One-way analysis of variance (ANOVA) followed by Dunnett’s post-analysis. Source data are available online for this figure.

**Figure 4:**
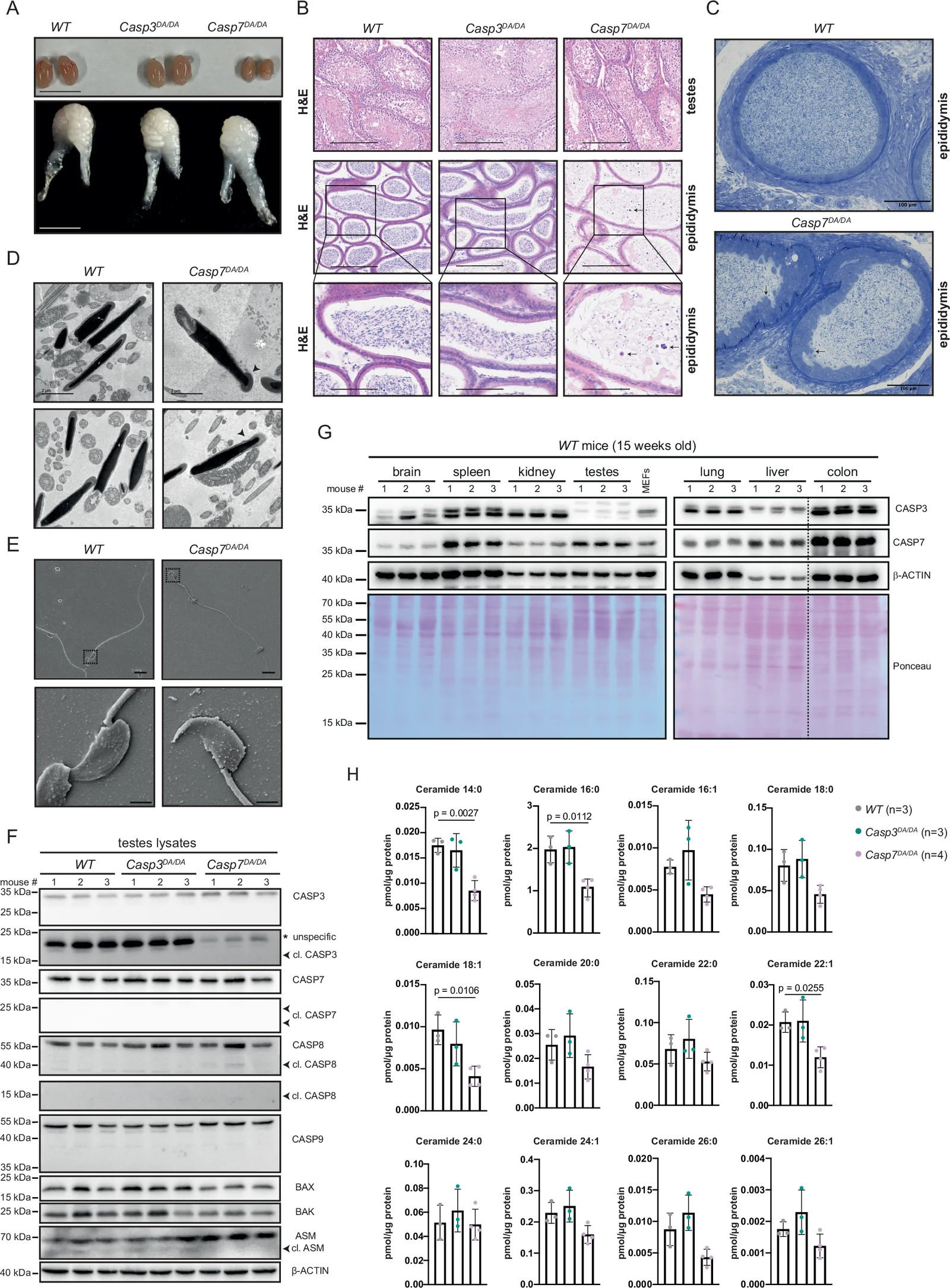
*Casp7*^*DA/DA*^ male mice are infertile and show altered spermatogenesis. (**A**) Representative images of testes (top) and epididymides (bottom) from *WT*, *Casp3*^*DA/DA*^, and *Casp7*^*DA/DA*^ mice at 30 weeks of age. Scale bars: 1.0 cm (top) and 0.3 cm (bottom). (**B**) Representative H&E-stained sections of testes and epididymides from 30-week-old mice. Arrows indicate sloughing phenomenon. Scale bars: 0.25 mm (top and middle) and 0.1 mm (bottom). (**C**) Transmission electron microscopy (TEM) analysis of epididymis sections from 30-week-old mice. Arrows indicate apical snouts. Scale bars: 100 µm. (**D**) TEM analysis of sperm from *WT* and *Casp7*^*DA/DA*^ mice. Arrows indicate deformed acrosomes. Scale bars: 2 µm. (**E**) SEM analysis of sperm from *WT* and *Casp7*^*DA/DA*^ mice. Scale bars: 10 µm (top) and 2 µm (bottom). (**F**) WB analysis of testes lysates from 30-week-old *WT* (*n* = 3), *Casp3*^*DA/DA*^ (*n* = 3), and *Casp7*^*DA/DA*^ (*n* = 3) mice. Lanes represent individual mice. Lysates were analysed for the expression of the indicated proteins. Positive control for cl. CASP3 is included in Fig. EV4E. (**G**) WB analysis of lysates from indicated tissues from 15-week-old *WT* mice (*n* = 3). Lanes represent individual mice. Lysates were analysed for the expression of the indicated proteins. (**H**) Concentration of different ceramides [pmol/µg protein] in testes from *WT* (*n* = 3), *Casp3*^*DA/DA*^ (*n* = 3), and *Casp7*^*DA/DA*^ (*n* = 4) mice. Dots represent individual mice. Data are mean ± s.d. One-way analysis of variance (ANOVA) followed by Dunnett’s post-analysis compared to the corresponding *WT* values. Source data are available online for this figure.

Although *Casp7*^*DA/DA*^ mice appeared healthy, male mice proved infertile (Fig. EV4A). Morphologically, testes isolated from *Casp7*^*DA/DA*^ mice appeared slightly smaller (Fig. 4A), but histologic analysis of adult testes did not reveal gross abnormalities in the tissue architecture (Fig. 4B). However, in the epididymis of *Casp7*^*DA/DA*^ mice, an increased sloughing phenomenon could be observed which might indicate alteration in the spermatid maturation process (Fig. 4B). Furthermore, transmission electron microscopy (TEM) revealed an irregular luminal epithelial surface with prominent apical snouts in the epididymis of *Casp7*^*DA/DA*^ mice, in contrast to the smooth luminal surface observed in wild-type tissue. The epithelial layer in *Casp7*^*DA/DA*^ mice appeared also thicker compared to wild-type (Fig. 4C).

In order to investigate the functionality of sperm, we sampled mouse sperm from the cauda epididymis of sexually mature mice. We could, however, barely isolate sperm from *Casp7*^*DA/DA*^ mice and the total numbers of isolated sperm appeared very low (Fig. EV4B,C). TEM did not show a gross morphological alteration in sperm isolated from *Casp7*^*DA/DA*^ mice, except from mild alterations of the plasma membrane and acrosome shape (2 mice per genotype) (Fig. 4D). Detailed microscopic analysis of sperm (head and neck) using high-resolution 3D images by SEM, however, did not reveal obvious morphological changes of sperm isolated from *Casp7*^*DA/DA*^ mice (Fig. 4E). Importantly, similar to *Casp7*^*DA/DA*^ mice, in *Bax*-deficient male mice, the testes display a disruption in germ cell development and increased cell death (apoptosis), leading to testicular atrophy and infertility (Knudson et al, 1995). Accordingly, our data suggested that the activation of CASP7, likely downstream of BAX-mediated mitochondrial apoptosis, represents the decisive event for spermatogenesis. Yet, histological and WB analyses of testes isolated from *Casp7*^*DA/DA*^ mice revealed no changes in apoptosis (Figs. 4F and EV4D,E) indicating that CASP7 activation controls spermatogenesis independent of apoptosis.

Spermatogenesis represents a complex series of events involving the establishment of a stem cell population, mitosis, meiosis, and the morphogenesis of the haploid germ cell, which collectively require the coordinated expression of more than 2300 different genes (Schultz et al, 2003). Therefore, we decided to perform a transcriptome analysis of mouse testes to monitor transcriptional alterations appearing in *Casp7*^*DA/DA*^ mice (Datasets EV1 and EV2). Hallmark gene set enrichment analysis (GSEA) identified upregulation of several gene sets involved in biological processes such as cholesterol homeostasis, fatty acid metabolism, and apoptosis (including pro- and anti-apoptotic genes) (normalised enrichment score (NES) of 2.5) (Fig. EV4F and Table EV1). The top gene ontology term which was downregulated in *Casp7*^*DA/DA*^ mice was spermatogenesis (NES of below 2.5), with a significant downregulation of beyond 60% genes in the gene set. GSEA using gene sets from biological processes further revealed that the lack of CASP7 activation in testicular tissues dramatically reduces the expression of genes involved in several processes coordinating spermatogenesis (Fig. EV4G; Dataset EV3). These data collectively indicated that CASP7 activation controls spermatogenesis, unlikely by involving apoptosis.

Analysis of CASP3 and CASP7 expression levels in different mouse tissues (3 mice) revealed that both CASP3 and CASP7 were strongly expressed in tissues such as spleen and colon (Fig. 4G). Strikingly, CASP3 was barely detectable in testes. In contrast, CASP7 was strongly expressed in testes, but its expression was less evident in brain or kidney. This is completely in line with the data derived from the tissue-specific atlas of mouse protein expression (Huttlin et al, 2010) and indicate that the dependency of testicular tissue on CASP7 is due to the lack of CASP3. It is important to note that the terminal differentiation of sperm shares many morphological and biochemical features with apoptosis such as membrane blebbing and cytoplasmic fragmentation/extrusion without leading to cell death (Blanco-Rodriguez and Martinez-Garcia, 1999; Said et al, 2004). In *Drosophila*, caspase activity has been implicated in terminal differentiation of sperm by promoting cytoplasmic extrusion leading to spermatid individualization independent of cell death (Arama et al, 2003; Kaplan et al, 2010). Although the role of caspases in mammalian spermatogenesis remains to be established, several studies involving mice, rats, and human tissues provided some evidence that caspase activation is required for testicular germ cell development, cytoplasmic shedding, spermatogenesis, and spermatid maturation (Blanco-Rodriguez and Martinez-Garcia, 1999; Sinha Hikim et al, 2003). Furthermore, CASP7 activation has been lately shown to be involved in spermatogenesis and male fertility by providing nonapoptotic functions (Lei et al, 2017). Our data collectively showed that the activation of CASP7 is essential for male fertility in mice unlikely by involving apoptosis as cells expressing CASP7(D198A) were proved competent in apoptosis (Fig. 2). In particular, cells expressing CASP3(D175A) respond to BH3 mimetics just by activating CASP7, which in turn induced membrane blebbing leading to cellular shrinkage, but not apoptotic cell death (Fig. 2). Thus, proapoptotic signalling in tissues lacking CASP3 activity, such as testes, leads to activation of CASP7, which is unable to induce apoptotic death but mediates morphological changes and cellular shrinkage, both important for terminal differentiation of sperm. This notion is completely in line with the data obtained in *Bax*-deficient mice (Knudson et al, 1995), which also developed male infertility. Collectively, these observations indicate that the mitochondrial apoptotic pathway in testes leads to activation of CASP7 which is required for successful spermatogenesis.

The endolysosomal enzyme SMPD1 (also called acid sphingomyelinase (ASM)) is activated during intrinsic and extrinsic apoptosis (Edelmann et al, 2011; Kashkar et al, 2005) and cleaves the phosphorylcholine head group of sphingomyelins generating ceramide, a lipid that tends to coalesce on membranes forming budding microdomains (Andrews et al, 2014). CASP7 is required for the proteolytic activation of ASM in response to TNF or anthrax toxins (FlaTox) (Andrews et al, 2014; Edelmann et al, 2011; Nozaki et al, 2022). These studies showed that CASP7 cleaves the 72-kDa pro-ASM zymogen (p72) at the non-canonical cleavage site after aspartate 253, yielding an active 57 kDa ASM (p57) (Edelmann et al, 2011). In contrast to previous studies demonstrating ASM cleavage after acute induction of caspase activity in cultured cells or intestinal organoids by TNF or FlaTox, respectively (Edelmann et al, 2011; Nozaki et al, 2022), our WB analysis of testicular lysates did not reveal marked ASM processing in testes as the cleaved ASM (p57) was only barely detectable in *WT* and *Casp3*^*DA/DA*^ mice (Fig. 4F). The ASM zymogen (p72) was, however, slightly accumulated in *Casp7*^*DA/DA*^ mice possibly due to lack of proteolytic processing of ASM by CASP7(D198A). Although ASM enzymatic activity was reduced in *Casp3*^*DA/DA*^ and, in particular, in *Casp7*^*DA/DA*^ MEFs (Fig. EV4H), we were not able to detect any alteration of ASM enzymatic activity in crude testicular lysates derived from *Casp3*^*DA/DA*^ or *Casp7*^*DA/DA*^ mice (Fig. EV4I). Independently, it is important to note that similar to *Casp7*^*DA/DA*^ mice, *Asm*^*−/−*^ male mice are sub-fertile and show abnormalities in sperm maturation (Butler et al, 2007; Butler et al, 2002). Interestingly, male reproductive impairment has also been documented in patients and relevant mouse models with altered sphingolipid storage (Adamali et al, 1999; Rabionet et al, 2015; Rabionet et al, 2008; Trasler et al, 1998). Thus, activation of CASP7 in testicular tissue may control spermatogenesis by interfering with ceramide production. Therefore, we conducted an extensive mass spectrometry analysis of sphingolipid composition in mouse testes to monitor possible changes in testicular sphingolipid storage (Schull et al, 2015). The obtained data showed that several short- and long-chain ceramide species were significantly less abundant in *Casp7*^*DA/DA*^ mice (Figs. 4H and EV4J). Collectively, these data suggest that the failure of CASP7(D198A) to activate ASM could represent one of the underlying molecular mechanisms which are controlled by CASP7 and ensures male fertility. Our data, however, do not formally prove the involvement of ASM in this process.

### Casp3^DA/DA^ mice develop selective hyperplasia of lymphoid tissues

**Figure 5:**
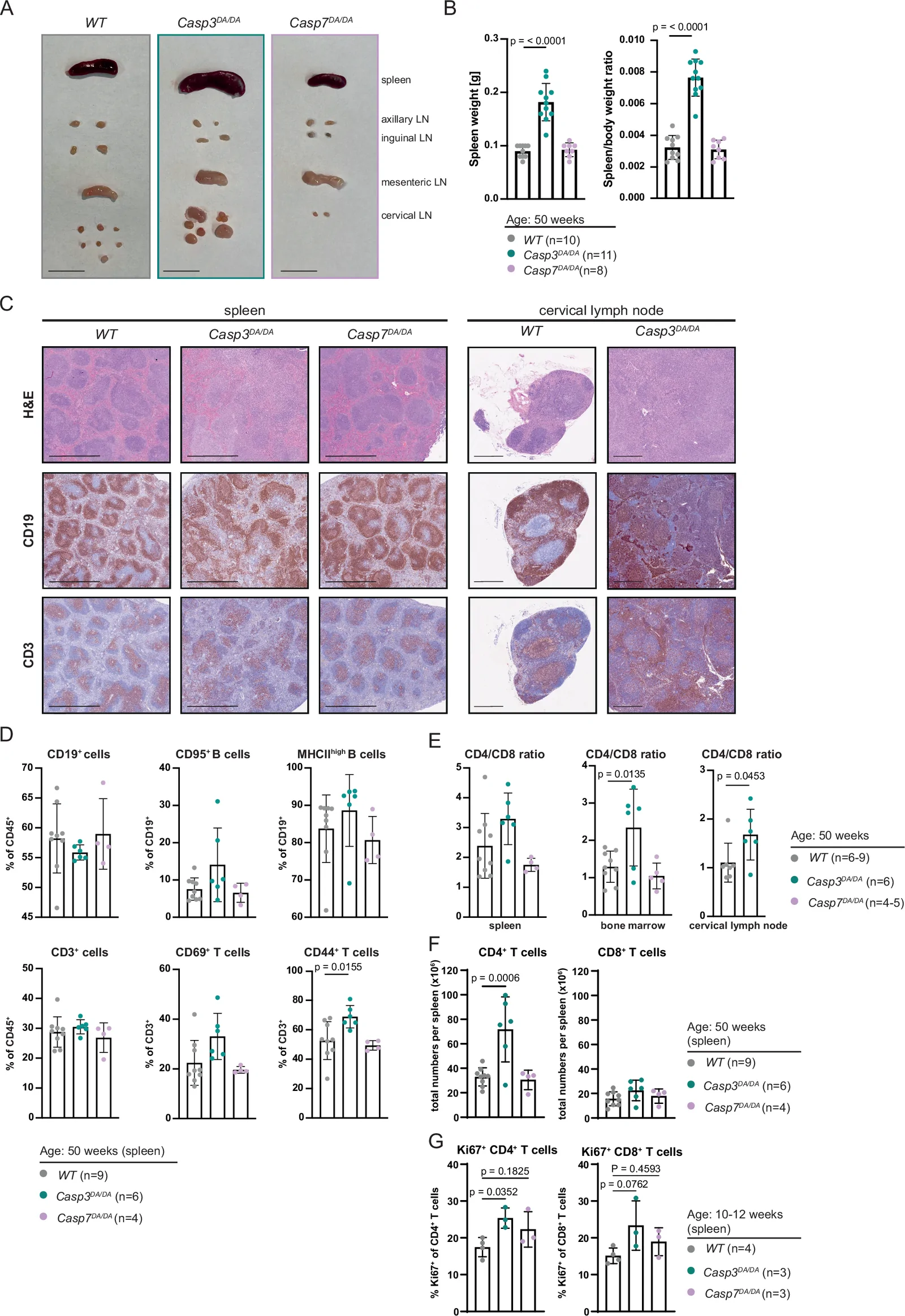
*Casp3*^*DA/DA*^ mice develop selective hyperplasia of lymphoid tissues. (**A**) Representative images of spleens and axillary, inguinal, mesenteric, and cervical lymph nodes (LN) from *WT*, *Casp3*^*DA/DA*^, and *Casp7*^*DA/DA*^ mice at 50 weeks of age. Scale bars: 1 cm. (**B**) Spleen weight and spleen/body weight ratio of 50-week-old mice. Dots represent individual mice. Data are mean ± s.d. One-way ANOVA with Dunnett’s post-analysis test compared to the corresponding *WT* values. Exact *P* values: 0.00000000521 (left) and 0.00000000004 (right). (**C**) Representative spleen sections from 50-week-old *WT* (*n* = 3), *Casp3*^*DA/DA*^ (*n* = 3), and *Casp7*^*DA/DA*^ (*n* = 3) mice and representative cervical lymph node sections from 50-week-old *WT* (*n* = 3) and *Casp3*^*DA/DA*^ (*n* = 3) mice stained with H&E and immunostained for CD19 and CD3. Scale bars: 1.0 mm (spleen) and 0.5 mm (cervical lymph nodes). (**D**) Flow cytometry analysis of single cell suspensions from spleens of 50-week-old *WT* (*n* = 9), *Casp3*^*DA/DA*^ (*n* = 6), and *Casp7*^*DA/DA*^ (*n* = 4) mice for CD19, CD95, MHCII, CD3, CD69, and CD44 surface expression. Dots represent individual mice. Data are mean ± s.d. One-way ANOVA with Dunnett’s post-analysis test compared to the corresponding *WT* values. (**E**) Flow cytometry analysis of T cell CD4/CD8 ratio from spleens, bone marrow, and cervical lymph nodes of 50-week-old mice. Dots represent individual mice. Data are mean ± s.d. One-way ANOVA with Dunnett’s post-analysis test compared to the corresponding *WT* values for data from spleen and bone marrow. Unpaired *t* test was performed for data from cervical lymph nodes. (**F**) Flow cytometry analysis of total numbers of CD4^+^ and CD8^+^ T cells in the spleen of 50-week-old *WT* (*n* = 9), *Casp3*^*DA/DA*^ (*n* = 6), and *Casp7*^*DA/DA*^ (*n* = 4) mice. Dots represent individual mice. Data are mean ± s.d. One-way ANOVA with Dunnett’s post-analysis test compared to the corresponding *WT* values. (**G**) Flow cytometry analysis of % Ki67 positive CD4^+^ and CD8^+^ T cells in the spleen of 10-12-week-old *WT* (*n* = 4), *Casp3*^*DA/DA*^ (*n* = 3), and *Casp7*^*DA/DA*^ (*n* = 3) mice. Dots represent individual mice. Data are mean ± s.d. One-way ANOVA with Dunnett’s post-analysis test compared to the corresponding *WT* values. Source data are available online for this figure.

**Figure EV5:**
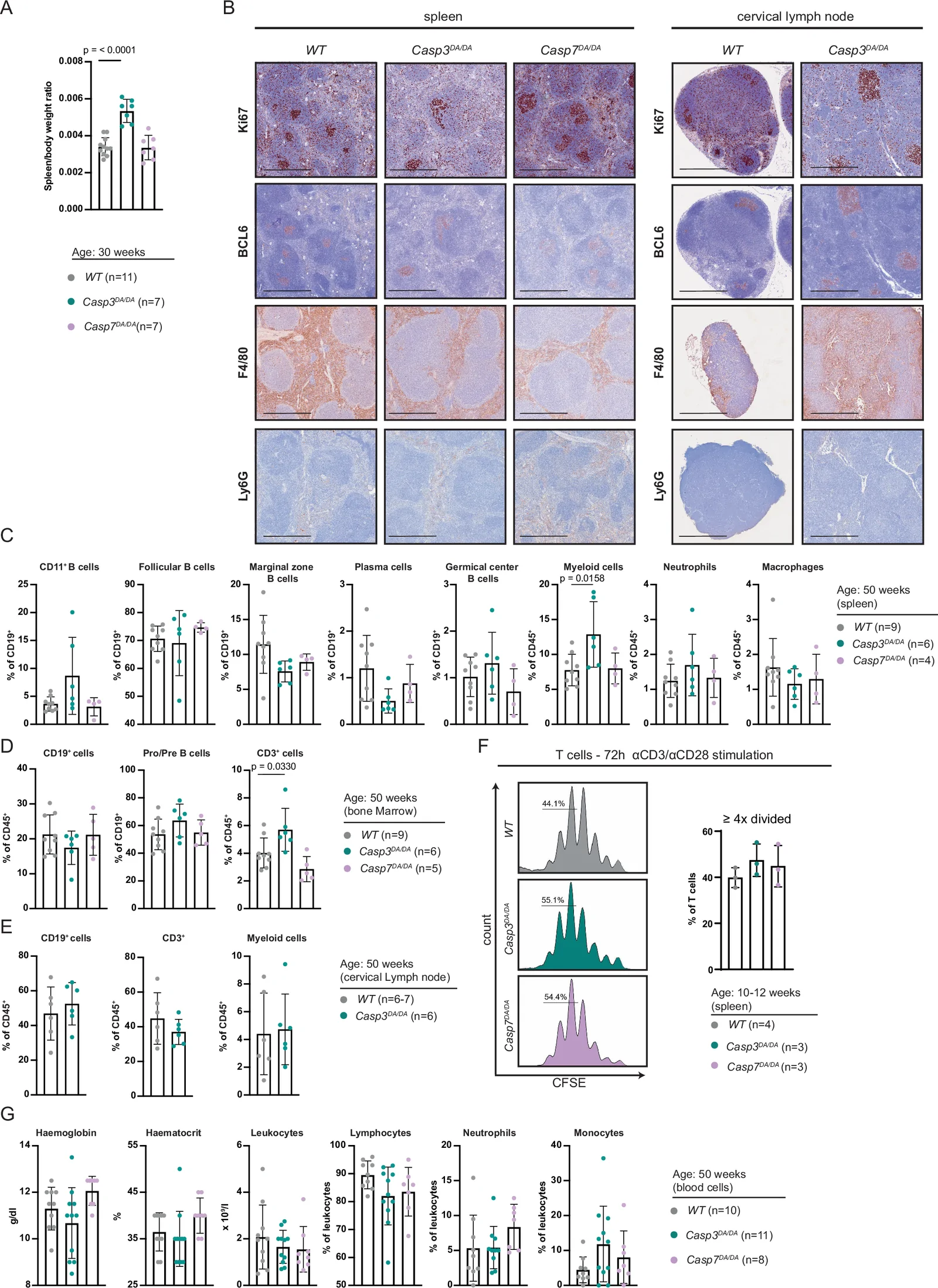
*Casp3*^*DA/DA*^ mice develop selective hyperplasia of lymphoid tissues. Related to Fig. 5. (**A**) Spleen/body weight ratio of 30-week-old mice. Dots represent individual mice. Data are mean ± s.d. One-way ANOVA with Dunnett’s post-analysis test compared to the corresponding *WT* values. (**B**) Representative spleen sections from 50-week-old *WT*, *Casp3*^*DA/DA*^, and *Casp7*^*DA/DA*^ mice and representative cervical lymph node sections from 50-week-old *WT* and *Casp3*^*DA/DA*^ mice immunostained for Ki67, BCL6, F4/80, and Ly6G. Scale bars: 1.0 mm (spleen) and 0.5 mm (cervical lymph node). (**C**) Flow cytometry analysis of immune cell populations in spleens from 50-week-old *WT* (*n* = 9), *Casp3*^*DA/DA*^ (*n* = 6), and *Casp7*^*DA/DA*^ (*n* = 4) mice. Dots represent individual mice. Data are mean ± s.d. One-way ANOVA with Dunnett’s post-analysis test compared to the corresponding *WT* values. (**D**) Flow cytometry analysis of immune cell populations in bone marrows from 50-week-old *WT* (*n* = 9), *Casp3*^*DA/DA*^ (*n* = 6), and *Casp7*^*DA/DA*^ (*n* = 5) mice. Data are mean ± s.d. One-way ANOVA with Dunnett’s post-analysis test compared to the corresponding *WT* values. (**E**) Flow cytometry analysis of immune cell populations in cervical lymph nodes from 50-week-old *WT* (*n* = 6) and *Casp3*^*DA/DA*^ (*n* = 6) mice. Data are mean ± s.d. Significance was tested by unpaired *t* test. (**F**) T cells, isolated from spleens of 10-12-week-old *WT* (*n* = 3), *Casp3*^*DA/DA*^ (*n* = 3), and *Casp7*^*DA/DA*^ (*n* = 3) mice, were labelled with CFSE proliferation dye and stimulated with anti-CD3/CD28 antibodies for 72 h. CFSE signal was measured on a Gallios flow cytometer. Top: representative histograms of CFSE signal, bottom: percent of T cells that divided ≥4 times. Dots represent individual mice. Data is presented as mean ± s.d. One-way ANOVA with Dunnett’s post-analysis test compared to the *WT* value. (**G**) Analysis of cardiac blood from 50-week-old *WT* (*n* = 10), *Casp3*^*DA/DA*^ (*n* = 11), and *Casp7*^*DA/DA*^ (*n* = 6) for haemoglobin concentration, haematocrit, and immune cell numbers. Dots, individual mice. Data are mean ±  s.d. One-way ANOVA with Dunnett’s post-analysis test compared to the corresponding *WT* values.

In contrast to *Casp7*^*DA/DA*^ mice, *Casp3*^*DA/DA*^ mice were fertile without any obvious signs of altered reproductivity (Fig. EV4A). They, however, showed hyperplasia of lymphoid tissues including spleen and cervical lymph nodes, presented at 6 months after birth (Figs. 5A,B and EV5A). Histological assessment revealed a diffuse, nodal hyperplastic enlargement and effacement of the splenic white pulp in *Casp3*^*DA/DA*^ mice (Figs. 5C and EV5B). Immunohistochemical staining for B- and T-cell markers showed especially a diffuse enlargement of the mantle zone as well as the central white pulp. The germinal centre architecture remained widely intact (BCL6 expression) (Figs. 5C and EV5B). In contrast to *Casp3*^*DA/DA*^ mice, the effacement of the white pulp was not evident in *Casp7*^*DA/DA*^ mice. In cervical lymph nodes of *Casp3*^*DA/DA*^ mice, extensive lymphofollicular hyperplasia was observed, characterized by enlarged lymph follicles and effacement of the lymphatic architecture (Fig. 5C). CD19- and CD3-staining revealed an expansion particularly of the mantle zone, as well as a slight enrichment of interfollicular T cells. Staining for Ki67 showed a regular proliferative activity, mainly limited to the germinal centres. Moreover, staining for BCL6 displayed an intact germinal centre architecture (Fig. EV5B). These data together indicated that the lack of CASP3 activation leads to lymphoid hyperplasia without altering a distinct cellular fraction within the secondary lymphatic tissues. In line with these observations, flow cytometric analysis examining the proportion of different cellular composition in spleens, cervical lymph nodes, and bone marrows, did not reveal major differences in the percentages of B and T cells (Fig. EV5C–E). The absolute counts of splenocytes in *Casp3*^*DA/DA*^ mice increased quite linear to the weight of the spleen and absolute numbers of nearly all immune cells were significantly higher in *Casp3*^*DA/DA*^ mice (Appendix Fig. S1). CD95^+^ and MHC class II^+^ B cells and CD69^+^ and CD44^+^ memory T cells appeared slightly increased in *Casp3*^*DA/DA*^ mice (Fig. 5D). Furthermore, the CD4 to CD8 T cell ratio was slightly increased in spleens, cervical lymph nodes, and bone marrow. In spleens, this was associated with elevated total CD4^+^ T cell numbers in *Casp3*^*DA/DA*^ mice (Fig. 5E–G). In vitro activation of T-cell receptor signalling by anti-CD3/anti-CD8 antibodies, however, failed to induce aberrant T cell proliferation, suggesting a TCR-independent induction of proliferation of T cells in *Casp3*^*DA/DA*^ mice (Fig. EV5F). Analysis of blood cells did not show any gross alteration of circulating cells (Fig. EV5G).

### Casp3^DA/DA^ mice develop chronic perivascular inflammation resembling STING-associated inflammatory disorder

**Figure 6:**
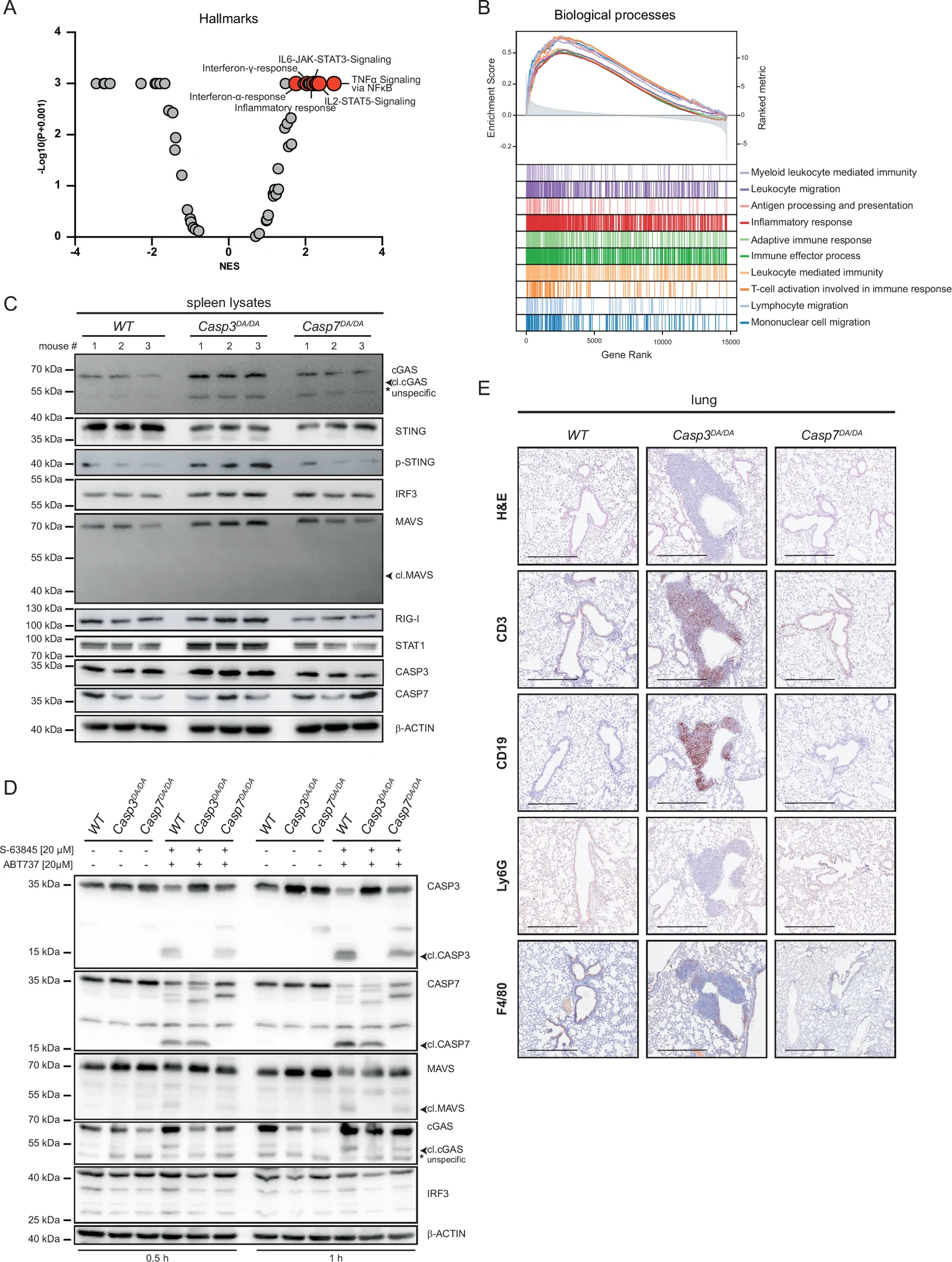
*Casp3*^*DA/DA*^ mice develop a chronic perivascular inflammation resembling STING-associated inflammatory disorder. (**A**) NES derived from GSEA comparing spleens from 30-week-old *WT* (*n* = 3) and *Casp3*^*DA/DA*^ mice (*n* = 3), using the hallmark gene set database. Significance was tested using default settings of the gseapy Python package (version 1.1.0), based on random permutation. (**B**) Enrichment plots of pathways (biological processes) involved in immune signalling, significantly upregulated in *Casp3*^*DA/DA*^ (*n* = 3) vs *WT* mice (*n* = 3). (**C**) WB analysis of spleen lysates from 50-week-old *WT* (*n* = 3), *Casp3*^*DA/DA*^ (*n* = 3), and *Casp7*^*DA/DA*^ (*n* = 3) mice. Lanes represent individual mice. Lysates were analysed for the expression of the indicated proteins. (**D**) WB analysis of MEFs with the indicated genotypes treated with S-63845 (20 µM) and ABT737 (20 µM) for 0.5 h and 1 h. (**E**) Representative lung sections from 50-week-old *WT*, *Casp3*^*DA/DA*^, and *Casp7*^*DA/DA*^ mice stained with H&E and immunostained for CD19, CD3, Ly6G, and F4/80. Scale bars: 0.5 mm. Source data are available online for this figure.

To gain more insight into the mechanisms involved in lymphoid hyperplasia, we performed a transcriptome analysis of total spleens from *WT* vs *Casp3*^*DA/DA*^ mice (Dataset EV4 and Table EV2). Hallmarks and biological processes GSEA identified upregulation of a number of gene sets associated with inflammation (Fig. 6A,B; Appendix Fig. S2, Table EV3 and Dataset EV5). In particular, a number of genes (beyond 50% in gene set) that are normally regulated *via* IFN signalling were significantly upregulated in *Casp3*^*DA/DA*^ mice (Fig. 6A,B; Appendix Fig. S2).

It is increasingly evident that caspases counteract IFN production (Xiong et al, 2021) and a previous in vitro study showed that apoptotic caspases proteolytically inactivate different components of inflammatory signalling that are involved in type I IFN production, including cyclic guanosine monophosphate‒adenosine monophosphate synthase (cGAS), mitochondrial antiviral-signalling protein (MAVS), and IFN regulatory factor 3 (IRF3) (Ning et al, 2019). Whereas cGAS-induced IFN production involves the adaptor protein stimulator of IFN genes (STING), MAVS serves as an adaptor for the cytosolic RNA sensors RNA helicases retinoic acid-inducible gene I (RIG-I)-induced IFN production (McNab et al, 2015). In line with the elevated IFN signalling detected in our transcriptome analysis, we found elevated expression of STAT1 as an IFN-responsive transcription factor in crude splenic lysates derived from *Casp3*^*DA/DA*^ mice (Fig. 6C). We could also detect increased protein levels of cGAS and MAVS in *Casp3*^*DA/DA*^ mice, possibly due to the lack of CASP3-mediated processing. However, cleaved cGAS, MAVS, or IRF3 was not detectable in crude splenic lysates derived from *WT* or *Casp7*^*DA/DA*^ mice. Importantly, in contrast to previous studies describing caspase-mediated cleavage of cGAS, MAVS, and IRF3 after acute caspase activation or ectopic caspase expression, in healthy spleens, apoptosis and CASP3 activation are not abundant (Figs. 1B and EV1B). Accordingly, cleaved cGAS or MAVS may not be sufficiently presented in crude splenic lysates for WB. In order to examine this notion, we exposed MEFs to BH3 mimetics to induce caspase activity (Fig. 6D). These analyses revealed that cGAS and MAVS were only cleaved in cells expressing wild-type CASP3. We could not detect IRF3 processing. Thus, CASP3(D175A) was not able to process cGAS and MAVS and thereby failed to control IFN production in *Casp3*^*DA/DA*^ mice.

Our data collectively indicated that the activation of CASP3 is required to dampen aberrant IFN production likely by inactivating/processing cGAS and/or MAVS. Upon sensing cytosolic DNA, cGAS binds and activates STING which subsequently recruits tank-binding kinase 1 (TBK1). TBK1, in turn, phosphorylates STING leading to IRF3-induced type-I IFN production (Zhang et al, 2019). Whereas the expression level of STING was not altered in the splenic lysates derived from mice, STING phosphorylation (p-STING) was evident only in *Casp3*^*DA/DA*^ mice (Fig. 6C), indicating that *Casp3*^*DA/DA*^ mice are exposed to aberrant/uncontrolled STING activation. Several autosomal dominant de novo or inherited mutations in STING, most frequently gain-of-function STING(N154S) mutation, induce IFN production and cause STING–associated vasculopathy with onset in infancy (SAVI) (Liu et al, 2014). SAVI patients develop systemic inflammation characterised by vasculopathy, interstitial lung disease associated with lung lymphocytic inflammatory infiltrates, and die prematurely. Homozygous gain-of-function STING(N153S) mutation in mice causes embryonic lethality. Heterozygous mice survive embryonal development but develop spontaneous disease, characterised by splenomegaly and pleural effusions which is associated with chronic perivascular inflammation in lung with a heterogeneous immune cell infiltration (CD19^+^ B cells, CD11b^+^ myeloid cells, CD4^+^ and CD8^+^ T cells) and organised thrombosis in pulmonary blood vessels (Warner et al, 2017). Similar to mice expressing STING(N153S), *Casp3*^*DA/DA*^ mice developed splenomegaly (Fig. 5B). Histological analysis of lungs derived from *Casp3*^*DA/DA*^ mice revealed subpleural accumulation of inflammatory cells, predominantly round cells, around a few vessels. The infiltrates were mainly composed of B cells, T cells, and macrophages resembling the majority of the inflammatory disorder described in mice expressing STING(N153S) (Fig. 6E). These data indicated that the lack of CASP3 activation leads to the manifestation of an inflammatory disorder which very closely resembles the inflammatory pathologies associated with the gain-of-function STING mutation in human and mouse. Thus, CASP3 activation is required to dampen STING-mediated inflammatory signalling and its anti-inflammatory function cannot be replaced by CASP7.

## Discussion

A key discovery in the field of programmed cell death (PCD) is the realisation that apoptosis is executed by an evolutionary conserved family of proteases called caspases (Alnemri et al, 1996; Yuan et al, 1993). During apoptosis, the C-terminal catalytic unit of caspases is cleaved into two chains which has long been considered as the decisive step for caspase maturation/activation. To investigate the biological significance of executioner caspase processing and to explore the physiological role of CASP3 and CASP7 activation, we established *Casp3*^*DA/DA*^ and *Casp7*^*DA/DA*^ mice. Our data demonstrated that proteolytic processing of the linker peptide of executioner caspases is required for their activation and cells expressing cleavage-resistant CASP3(D175A) or CASP7(D198A) react to apoptotic stimuli very similarly to cells lacking *Casp3* or *Casp7*, respectively. Similar to *Casp3*^*−/−*^*Casp7*^*−/−*^ mice (Lakhani et al, 2006), *Casp3*^*DA/DA*^*Casp7*^*DA/DA*^ mice died before or shortly after birth, providing additional evidence that the proteolytic processing of executioner caspases is essential for their activation during embryonic development of mice. However, unlike CASP9 deficiency in mice, which leads to defects in neuronal tube closure during embryonic development (Kuida et al, 1998), *Casp3*^*DA/DA*^*Casp7*^*DA/DA*^ mice lacking the CASP9-specific cleavage sites, did not manifest neural alterations, indicating that neither CASP3 nor CASP7 is decisive for embryonic neuronal development. These observations are in line with previous data obtained in *Casp3*^*−/−*^*Casp7*^*−/−*^ mice (Lakhani et al, 2006) and raise the question whether CASP9 and the formation of the apoptosome lead to processing of substrates other than executioner caspases which are involved in neuronal development. Alternatively, whereas CASP9 deficiency selectively disables the mitochondrial apoptotic cascade, lack of executioner caspase activation in *Casp3*^*DA/DA*^*Casp7*^*DA/DA*^ mice blocks the extrinsic apoptotic pathway as well, which may potentially interfere with the embryonic development of mice. Collectively, these observations suggest that further extensive investigations are required to monitor the role of caspases in neuronal tissues during embryonic development.

CASP3 and CASP7 represent the two major executioner caspases that are closely related and have long been considered to have a virtually indistinguishable substrate specificity during apoptosis (Stennicke et al, 2000). On the one hand, during embryonic development the activation of CASP3 or CASP7 seemed to act redundantly, either by controlling apoptosis or other cellular processes, which needs further in-depth investigations. On the other hand, in adult animals, CASP3 and CASP7 appeared to provide non-redundant functions in different tissues, unlikely as drivers of apoptosis. Our data showed that the proteolytic activation of CASP3 is important to control inflammatory signalling and lymphoid tissue homeostasis, whereas activation of CASP7 appeared to ensure spermatogenesis and male fertility. It is important to note that CASP3 was barely detected in testes, but both CASP3 and CASP7 were comparably expressed in the spleen. This indicates that the dependency on either CASP3 or CASP7 activation was not only due to the lack of the second executioner caspase. A previous study using human cell lines and cell-free systems already indicated that CASP3 and CASP7 exhibit differential activity toward multiple cellular substrates, not exclusively involved in apoptosis (Walsh et al, 2008). Furthermore, the major structural difference between CASP3 and CASP7 seems to be in their N-terminal peptides which directs targeting of different cellular processes (Fuentes-Prior and Salvesen, 2004). Importantly, previous studies using caspase gene knockout models failed to provide robust information, in particular, in a comparable manner due to the strain-specific genetic background effects of mice deficient in either caspase. Accordingly, CASP3-deficient 129/SvJ mice died during the perinatal period due to neuronal defects, whereas CASP3-deficient C57BL/6 mice, which were established by backcrossing of *Casp3*^*−/−*^ 129/SvJ mice onto the C57BL/6 background (10 generations), were viable and did not exhibit a severe phenotype (Leonard et al, 2002). Furthermore, 129/SvJ mice with CASP7-deficiency were initially reported to die during embryonic development (Zheng et al, 1999) and were later described to have a normal appearance (Lakhani et al, 2006). Defects in spermatogenesis have not been reported in earlier studies describing CASP7-deficient mice. These mice were backcrossed onto the C57BL/6 background (6 generations) in order to perform comparable physiological analysis between the two executioner caspases (Lakhani et al, 2006). In contrast, we established *Casp3*^*DA/DA*^ and *Casp7*^*DA/DA*^ mice on a clean C57BL/6 N background, providing a robust and reliable comparison of the physiological functions of these two executioner caspases.

A constantly growing body of research indicates that caspase activation may not necessarily lead to cell death (Aram et al, 2017; Maekawa et al, 2023). Our observation that the activation of CASP7 is required for terminal differentiation of sperm sheds new light on the physiologic roles of caspases. This is in line with a number of independent observations in mice and human, implicating caspases in the pathogenesis of multiple andrological pathologies such as impaired spermatogenesis, decreased sperm motility, testicular torsion, varicocele, and immunological infertility (Said et al, 2004). Unlike CASP7, CASP3 activation was responsible for the regulation of inflammation and lymphoid tissue homeostasis. This is consistent with accumulating evidence highlighting the central regulatory role of caspases in IFN signalling and provides solid evidence that mainly CASP3, and not CASP7, controls IFN production. Strikingly, *Casp3*^*DA/DA*^ mice developed a phenotype which was very similar to a disease observed in patients or mice carrying a gain-of-function STING mutation. Together, our study explored two major functions of executioner caspases that unlikely involve apoptosis.

Finally, aberrant caspase activity has been linked to the pathogenesis of various diseases such as inflammatory and metabolic diseases, neurological disorders, and cancer (Dhani et al, 2021). Consequently, a number of different caspase inhibitors have been designed and developed as a potential therapeutic tool. However, their application is limited to preclinical studies due to numerous challenges. In particular, our knowledge of the physiological role of caspases is fragmentary and the use of knockout mouse models provides a rather inadequate condition to monitor the physiological outcomes of caspase inhibition. This was recently demonstrated in mice expressing enzymatically inactive CASP8 which developed a very different phenotype compared to those with a *Casp8* gene knockout (Fritsch et al, 2019; Newton et al, 2019). Mice expressing cleavage-resistant executioner caspases resemble conditions when cells are exposed to caspase inhibitors and can serve as a valuable tool for drug testing and for treatment predictions.

## Methods

**Table:** Reagents and tools table

Reagent/resource	Reference or source	Identifier or catalog number
**Experimental models**		
C57BL/6 N M. musculus, indicated genotypes	This study	–
Immortalized murine fibroblasts, indicated genotypes	This study	–
**Recombinant DNA**		
**Antibodies**		
CASP3	Cell Signaling	14220
cl. CASP3	Cell Signaling	9661
CASP6	Cell Signaling	9762
cl. CASP6	Cell Signaling	9761
CASP7	Cell Signaling	12827
cl. CASP7	Cell Signaling	9491
CASP8	Cell Signaling	4927
cl. CASP8	Cell Signaling	9429
CASP9	Cell Signaling	9504
cl. CASP9	Cell Signaling	9509
RIPK1	Cell Signaling	3493
RIPK3	ENZO	ADI-905-242-100
MLKL	Sigma-Aldrich	MABC604
p-MLKL	Abcam	Ab196436
BID	R&D	AF860
PARP	BD Pharmingen	556362
MAVS	Cell Signaling	4983
RIG-I	Cell Signaling	3743T
cGAS	Cell Signaling	31659
STING	Cell Signaling	50494
p-STING	Cell Signaling	72971S
STAT1	Cell Signaling	14994
CASP1	Adipogen	AG-20B-0042-C100
GSDM D	Abcam	Ab219800
GSDM E	Abcam	Ab215191
IL-1ß	R&D Systems	AB-401-NA
IRF3	Cell Signaling	4302
BAX	Cell Signaling	2772
BAK	Cell Signaling	12105
ASM	Proteintech	14609-1-AP
ß-Actin	Santa Cruz	Sc47778
Anti-rabbit IgG	Cell Signaling	7074
Anti-mouse IgG (Light chain)	Cell Signaling	58802
Anti-rat IgG	Invitrogen	31470
Anti-goat IgG	Jackson ImmunoResearch	305035003
CD19	Invitrogen	14-0194-82
CD3	Epredia	RM-9107
Ki67	Cell Signaling	12202
BCL-6	Cell Signaling	5650
Ly6G	BD Pharmingen	551459
F4/80	Immunostep	MOF4PU
Anti-Rabbit	Medac Diagnostika	414341 F
Anti-Rat	Medac Diagnostika	414331 F
Ig λ Light Chain, anti-mouse, FITC	Miltenyi	130-098-415
PE anti-mouse CD23	BioLegend	101608
PerCP/Cyanine5.5 anti-mouse IgM	BioLegend	406512
PE/Cyanine7 anti-mouse CD19	BioLegend	152418
APC anti-mouse CD21/CD35	BioLegend	123412
Alexa Fluor® 700 anti-mouse Ig light chain κ	Biolegend	409508
CD93 Antibody, anti-mouse, APC-Vio® 770	Miltenyi	130-120-807
Pacific Blue™ anti-mouse IgD	BioLegend	405712
Brilliant Violet 510™ anti-mouse CD45	Biolegend	103138
FITC anti-mouse CD45	BioLegend	103108
PE anti-mouse CD95	BioLegend	152608
PerCP/Cyanine5.5 anti-mouse CD5	BioLegend	100624
APC anti-mouse CD138	BioLegend	142506
Alexa Fluor® 700 anti-mouse I-A/I-E	Biolegend	107622
Pacific Blue™ anti-mouse/human GL7 Antigen	BioLegend	144614
FITC anti-mouse TCR β chain	BioLegend	109206
PE anti-mouse/human CD11b	BioLegend	101208
PerCP/Cyanine5.5 anti-mouse/human CD44	BioLegend	103032
PE/Cyanine7 anti-mouse CD25	BioLegend	101916
APC anti-mouse CD69	BioLegend	104514
Alexa Fluor® 700 anti-mouse CD8a	BioLegend	100730
APC/Cyanine7 anti-mouse CD4	BioLegend	100414
Pacific Blue™ anti-mouse CD3	BioLegend	100214
FITC anti-mouse CD11c	BioLegend	117306
PerCP anti-mouse NK-1.1	BioLegend	108728
PE/Cyanine7 anti-mouse Ly-6C	BioLegend	128018
APC anti-mouse F4/80	BioLegend	123116
APC/Fire™ 750 anti-mouse Ly-6G	BioLegend	127652
FITC anti-mouse CD3	BioLegend	100204
FITC anti-mouse CD93	BioLegend	136508
PerCP/Cyanine5.5 anti-mouse CD117	BioLegend	135134
Alexa Fluor® 647 anti-mouse IgM	BioLegend	406526
Alexa Fluor® 700 anti-mouse CD45	BioLegend	103128
APC/Fire™ 750 anti-mouse CD34	BioLegend	128614
Alexa Fluor® 647 anti-mouse Ki-67	BioLegend	652407
Ultra-LEAF™ Purified anti-mouse CD3ε Antibody	BioLegend	100340
Ultra-LEAF™ Purified anti-mouse CD28 Antibody	BioLegend	102116
**Oligonucleotides and other sequence-based reagents**		
PCR primers	This study, synthesized at Eurofins	
**Chemicals, enzymes and other reagents**		
Birinapant	Biozol	TGM-T6007
TNF	R&D Systems	8230-CL-050
S-63845	CHEMGOOD	C-1370
ABT-737	Cell Signaling	93584S
LPS *E. coli* O111:B4	Sigma-Aldrich	L2630
Nigericin	Sigma-Aldrich	SML1779
Recombinant active caspase-8	Enzo Life Sciences	ALX-201-062?
Recombinant active caspase-9	Enzo Life Sciences	BML-SE240
DRAQ7^TM^	Biostatus	DR710HC
DNAse I recombinant	Roche	04536282001
Murine IL2	PeproTech	212-12-5UG
4% Formaldehyde	Walter CMP GmbH	WAL60622 5000
Citrate Buffer	Sigma-Aldrich	C9999-1000ML
Proteinase K	Thermo Fisher Scientific	EO0492
Cellpack	Sysmex	83400116
RPMI	Pan Biotech	P04-18500
DMEM	Bio&sell	BS.FG0435
RBC lysis buffer	BioLegend	420301
FBS	Biowest	S1810-500
ibidi µ-slide 8 Well^high^ Glass Bottom	ibidi	80807
L929	This study	
Osmiumtetroxid	Science Services	E19190
Potassium hexacyanoferrate	Sigma-Aldrich,	P8131
Cacodylate buffer	Applichem	A2140,0100
Hexamethyldisilazane	Sigma-Aldrich	440191-100 ML
Epon	Science Services,	E14120
TAAB capsules	Agar Scientific	G3744
uranyl acetate	Agar Scientific	R1260A
**Software**		
MultiQuant 3.0.3 software	AB SCIEX	
GraphPad Prism for macOS (version 8.2.1)	GraphPad Software	
Microsoft® Excel for Mac (version 16.78)	Microsoft	
Evolution-Capt	Vilber	
NDP.view2	Hamamatsu	
Kaluza Analysis 2.2	Beckman Coulter	
Sperm analyzer IVOS®	Hamilton Thorne	
IncuCyte® Live-Cell Analysis System	Sartorius	
Cutadpat tool (version 4.1)	https://doi.org/10.14806/ej.17.1.200	
STAR aligner (version 2.7.0e)	https://doi.org/10.1093/bioinformatics/bts635	
RSEM (version 1.3.1)	https://doi.org/10.1186/1471-2105-12-323	
gseapy Python package (version 1.1.0)	https://doi.org/10.1093/bioinformatics/btac757	
EVOS^TM^ M7000 Imaging System (Software 2.4.1468.172)	Thermo Fisher Scientific	
SEM SmartSEM Version 5.07	Zeiss	
**Other**		
RNeasy® Mini Kit	Qiagen	74106
Illumina Ribo-Zero Plus rRNA Depletion Kit	Illumina	
Illumina® Stranded Total RNA Sample Preparation Kit	Illumina	
Peqlab KAPA Library Quantification Kit	Roche	
Avidin/Biotin Blocking Kit	Vector Laboratories	VEC-SP-2001
ABC Kit Vectastain Elite	Vector Laboratories	VEC-PK-6100
DAB Staining Kit	Vector Laboratories	VEC-SK-4105
Click-iT™ TUNEL Colorimetric IHC Detection Kit	Invitrogen	C10625
In Situ Cell Death Detection Kit, TMR red	Roche	12156792910
DC Protein Assay Reagents Package	Bio Rad	5000116
Mouse IL-1 beta/IL-1F2 DuoSet ELISA	R&D Systems	DY401
True-Nuclear™ Transcription Factor Buffer Set	BioLegend	424401
Zombie Yellow™ Fixable Viability Kit	BioLegend	423103
CellTiter 96® AQueous One Solution Cell Proliferation Assay (MTS)	Promega	G3582
CD3ε MicroBead Kit, mouse	Mitenyi Biotec	130-094-973
CellTrace™ CFSE Cell Proliferation Kit	Thermo Fisher Scientific	C34554
Cytotoxicity Detection Kit (LDH)	Roche	11644793001
MSigDB (version 2023.1.Mm)	Broad Institute	
Precelly 24 Homogenisator	Peqlab	

### Mice

*Casp3*^*D175A/D175A*^ and *Casp7*^*D198A/D198A*^ mice were generated by CRISPR/Cas9 genome editing as described (Fritsch et al, 2019; Schorn et al, 2023; Theobald et al, 2021; Troder et al, 2018). 4 µM Alt-R^TM^ guide RNA (*Casp3*: 5’-GTGGCATTGAGACAGACAGT-3’ and *Casp*^*7*^: 5’-ATGGAATCCAGGCTGACTCG-3’) and 10 µM standard desalted Ultramer^TM^ single-stranded DNA repair template (*Casp3*^*175A*^: 5’-ATGTCTTCCGACACCCAGGCCTGCCGGGGTACGGAGCTGGACTGTGGCATTGAGACAGCTAGCGGTACTGATGAGGAGATGGCTTGCCAGAAGATACCGGTGGAGGCTGACTTCCTGTAT-3‘ and *Casp7*^*D198A*^: 5’- TACTTTGATATGCTTTAGGCATGCCGAGGGACGGAGCTCGATGATGGAATCCAGGCTGCTAGCGGTCCCATCAACGACATTGACGCTAATCCCCGCAACAAGATCCCGGTGGAAGCCGAC-3’) were electroporated with 4 µM Alt-R^TM^ SpCas9 Protein in C57BL/6 NRj zygotes. CRISPR reagents were purchased from Integrated DNA Technologies (USA) and animals obtained from Janvier Labs (France). Embryos were cultured in vitro and transferred to recipients for delivery. Correct integration of the mutation in the resulting F_0_ mice was validated by Sanger sequencing of purified PCR amplicon in the founder as well as the F_1_ mice. Genome editing was performed at the in vivo Research Facility of the CECAD Research Center University of Cologne, Germany.

Genotyping of DNA from ear biopsies was performed using Sanger sequencing (Microsynth SEQLAB) with PCR Primer for Casp3^D175A^ (5’-GCTTACTGGCTTGCTTCTCC-3’ and 5’-TGCAGTAGACAGGTGTAGGC-3’) and for Casp7^D198A^ (5’-GACCGATGCAAAACCCTGTT-3’ and 5’-TGCAGTAGACAGGTGTAGGC-3’).

Founder mice carrying the intended D175A or D198A mutation were backcrossed to C57BL/6N wild-type mice.

All animal protocols were performed in compliance with the European, national, and institutional guidelines and approved by the State Office of North Rhine-Westphalia, Department of Nature, Environment and Consumer Protection (LAVE NRW, Germany; animal study protocol AZ 2021.A123, AZ 2019.A335 and AZ 2025-1098).

All animals were housed under standard pathogen-free conditions with a 12-h light/dark cycle at the CECAD animal facility (University of Cologne, Germany). Food and water were provided *ad libitum*. There was no performance of study specific calculations regarding simple size, randomization, and blinding. Animals were grouped in accordance to their genotypes in mixed sexes.

### Embryology

For timed breeding, plug positivity was checked daily. Embryonic day count started at E0.5 when a positive plug was found. Isolation of embryos was conducted at E13.5 and E18.5. Photos of the isolated embryos were taken using an Olympus SZX16 microscope.

### Immunohistochemistry

Tissues from spleen, lymph node, testis, epididymis, liver, and lung were fixed in 4% formaldehyde solution (WALTER-CMP) for 24 h, embedded in paraffin and cut in 5 µm sections (Andree et al, 2014; Gunther et al, 2020).

Sections were stained with H&E or with antibodies against CD19, CD3, BCL6, Ki67, Ly6G, and F4/80 after rehydration and heat-induced antigen retrieval in 10 mM citrate buffer or by proteinase K treatment. Blocking was performed for 1 h using the Avidin/Biotin Blocking Kit (Vector Laboratories). The ABC Kit Vectastain Elite (Vector Laboratories) and the DAB staining kit (DAKO) were used for visualisation of the staining. Counterstaining was performed with hematoxylin (Carl Roth).

The detection of apoptotic cells in 5 µm testes sections was done using the Click-iT™ TUNEL Colorimetric IHC Detection Kit (Invitrogen). The staining was performed according to the manufacturer’s instructions. Stained sections were scanned with a NanoZoomer S360 Digital slide scanner (Hamamatsu) and analysed with the imaging software NDP.view2 (Hamamatsu).

### Blood parameter analysis

Following cervical dislocation of mice, blood from the heart was collected by an ETDA-coated syringe. Afterwards, blood was diluted with Cellpack (sysmex) in a ration of 1:5 and analysed at the Institute for Clinical Chemistry of the University Hospital Cologne.

### Flow cytometric analyses

Single cells suspensions from spleens and lymph nodes were collected by meshing the tissue through a 70 µm cell strainer. Bone marrow was isolated via flushing the femur and tibia with RPMI. After lysis of erythrocytes (red lysis buffer, BioLegend), cells were resuspended in cell staining buffer and stained with the indicated antibodies (Dataset EV6) for 15 min at RT. For Ki67 staining, dead cells were first labelled with Zombie Yellow™ Fixable Viability Kit (Biolegend). After extracellular staining, Ki67 was stained using the True-Nuclear™ Transcription Factor Buffer Set (Biolegend) according to manufacturer’s instruction. Flow cytometric analysis was performed on a Gallios flow cytometer (Beckman Coulter) and data was analysed by Kaluza Analysis 2.2 (Beckman Coulter).

### Sperm analysis

Mouse sperm were recovered according to standard procedures by removing vasa deferentia and cauda epididymides from male mice as described (Wigger et al, 2023). For counting, 10 µl of sperm was immobilised by adding 90 µl sterile H_2_O (Martinez, 2022). Sperm heads were counted using a Neubauer chamber and a light microscope, which was additionally used to take pictures of the sperm.

### MEFs and MDFs cell culture

Mouse embryonic fibroblasts (MEFs) were generated from embryos of the intended genotypes at E13.5. Mouse dermal fibroblasts (MDFs) were generated from tails of mice with the intended genotypes. *Casp3*^*−/−*^ and *Casp7*^*−/−*^ cells were generated by treating *Casp3*^*fl/fl*^ and *Casp7*^*fl/fl*^ cells (Ghazavi et al, 2022) with a cell-permeable active Cre protein (HTNCre) (Fritsch et al, 2019; Schiffmann et al, 2020). Cells were cultured in Dulbecco’s Modified Eagle Medium (DMEM) (Bio&Sell) supplemented with 10% heat-inactivated fetal bovine serum (Biowest), 10 mM HEPES (pH 7.2) (Fisher BioReagents), 1 mM sodium pyruvate (Bio&Sell), 1x nonessential amino acids (SclenCell), 100 U ml^−1^ penicillin, and 100 μg ml^−1^ streptomycin (Bio&Sell). Immortalisation of cells was performed by using simian virus 40 (SV40) transduction. Cells were regularly tested for mycoplasma contamination.

### TUNEL staining of MEFs

TUNEL assay was performed using In Situ Cell Death Detection Kit, TMR red (Roche, cat. no. 12 156 792 910). Cells were seeded in ibidi µ-slide 8 Well^high^ Glass Bottom (ibidi, cat. no. 80807) coated with 0,2% gelatine. After treatment, cells were fixed in 4% PFA for 60 min at room temperature followed by washing with PBS. Permeabilisation was performed by incubating samples with freshly prepared 0.1% Triton X-100 in 0.1% sodium citrate for 2 min on ice. Afterwards, the samples were washed with PBS and the positive control was generated by incubation with 100 µl recombinant DNAse I (2500 U/ml, Roche, cat. no. 04536282001) for 10 min at room temperature. Afterwards, TUNEL reaction mixture (1 part enzyme, 9 parts labelling solution) was added to samples and incubated for 60 min at 37 °C in humidified atmosphere in the dark. Finally, cells were washed with PBS containing DAPI and analysed with an EVOS^TM^ M7000 (Thermo Fischer Scientific).

### Metabolic activity

Per 96-well, 1 × 10^4^ MEFs were plated. On the following day, cells were treated as indicated in 100 µl total volume. After 2 and 24 h, 20 µl of CellTiter 96® AQueous One Solution (Promega) was added and absorbance at 490 nm was measured after 1 h. Metabolic activity was calculated relative to the untreated control. Cells treated with 0.1% of Triton X were used as negative control.

### Macrophage differentiation and treatment

Mouse bone marrow derived macrophages (BMDMs) were differentiated by culturing isolated bone marrow in RPMI supplemented with 15% L929-conditioned medium, 10% heat-inactivated fetal bovine serum (Biowest), 10 mM Hepes (pH 7.2) (Fisher BioReagents), 1 mM sodium pyruvate (Bio&Sell), 1× nonessential amino acids (SclenCell), 100 U ml^−1^ penicillin, and 100 μg ml^−1^ streptomycin (Bio&Sell) for seven days. For WB analysis, 2 × 10^6^ cells were plated per 6-well. On the following day, cells were pre-treated with 200 ng/ml LPS for 4 h, followed by additional nigericin treatment (10 µM) for 2 h. Supernatants were collected for IL-1β ELISA and LDH release.

### IL-1β ELISA

Il-1β release was measured by ELISA (R&D Systems). Procedure was performed according to manufacturer’s protocol. Absorbance at 450 nm with reference wavelength at 540 nm was measured on a Tecan GENious Pro plate reader.

### LDH release

LDH Release was measured using Cytotoxicity Detection Kit (LDH) (Roche). Procedure was performed according to manufacturer’s protocol. Supernatant were transferred to a 96-well plate and mixed with freshly prepared LDH reaction solution (1:46) and incubated for 20 min at RT. Absorbance at 490 nm was measured on a Tecan GENious Pro plate reader. To normalise LDH release, the percentage was calculated as followed %= (sample − untreated sample)/(Triton control − untreated sample).

### Cell death measurements

Per 96-well, 1 × 10^4^ cells were plated overnight. The next day, cells were treated in the presence of 0.15 µM DRAQ7^TM^ (biostatus) with the indicated stimuli: birinapant (Biozol, cat. no. TGM-T6007), TNF (R&D Systems, cat. no. 8230-CL-050), emricasan (Biozol, cat. No. SEL-S7775), S-63845 (CHEMGOOD, cat. no. C-1370), ABT-737 (Cell Signaling, cat. no. 93584S), LPS (*E. coli* O111:B4; Sigma-Aldrich, cat. no. L2630), and nigericin (Sigma-Aldrich, cat. no. SML1779) as indicated. For birinapant treatment, inhibitor was added 1 h before in order to induce cIAP1/2 depletion. Cell death was measured using the IncuCyte® Live-Cell Analysis System (Sartorius) using the basic or cell-by-cell analysis (IncuCyte 2021 C). Percent cell death was calculated by normalising Draq7 positive cells by the total number of cells.

### Proliferation analysis of T cells

T cells were isolated from single cell suspensions of spleens from 10-12-week-old mice using the CD3ε MicroBead Kit (Miltenyi) according to manufacturer’s instructions. T cells were then labelled with the CellTrace™ CFSE Cell Proliferation Kit (Invitrogen) according to manufacturer’s instructions using 1 µM of CFSE. For stimulation, wells of a 6-well plate were coated with Ultra-LEAF™ Purified anti-mouse CD3ε Antibody (BioLegend) (5 µg/ml anti-CD3 in 800 µl PBS per well) for 2 h at 37 °C. Per well, 5 × 10^6^ (2 × 10^6^/ml) T cells were added, cultured in RPMI (Bio&Sell), supplemented with 10% heat-inactivated fetal bovine serum (Biowest), 2 mM sodium pyruvate (Bio&Sell), 100 U ml^−1^ penicillin, and 100 μg ml^−1^ streptomycin (Bio&Sell), 50 µM 2-mercaptoethanol (Gibco), 2 µg/ml Ultra-LEAF™ Purified anti-mouse CD28 Antibody (BioLegend), and 10 ng/ml IL-2 (PeproTech). After 72 h incubation, wells were harvested and CFSE signal was measured on a Gallios flow cytometer (Beckman Coulter) and data was analysed by Kaluza Analysis 2.2 (Beckman Coulter).

### Cytosolic extracts

For the generation of cytosolic extracts, lysis of cells was conducted on ice in Hypotonic Lysis Buffer (HEB; 20 mM PIPES pH 7.4, 50 mM KCl, 2 mM MgCl_2_, 5 mM EDTA pH 8.0, 1 mM DTT). Cells were passaged through a 26 G x ½ 0,45 ×13 mm needle (BD) to disrupt the plasma membrane and centrifuged for 20 min at 20,000×*g* at 4 °C. Following, supernatants were collected as the cytosolic protein fraction (Kashkar et al, 2002; Schull et al, 2015).

### Caspase activity assay

Measurement of caspase activity was described previously (Gunther et al, 2020; Kashkar et al, 2003). Briefly, cytosolic extracts were treated with recombinant active caspase-8 or-9 (Enzo Life Sciences) for 1 h at 30 °C and 300 rpm. Subsequently, 2 µl of the reaction were transferred in a 96-well black microtiter plate into 50 µl of caspase activation buffer (10 mM HEPES pH 7.2, 100 mM NaCl, 0.1 mM EDTA, 0.1% CHAPS, 10% sucrose, 10 mM DTT). To measure caspase-3/-7 activity, the reaction was started by adding 100 µM fluorometric substrates (Ac-DEVD-AFC). A Tecan GENious Pro was used to measure fluorescence intensity at 400/505 nm.

### Western blot (WB) analysis

Lysis of cells and tissues was conducted on ice in 20 mM Tris-HCL pH 7.5, 135 mM NaCl, 1.5 mM MgCl_2_, 1 mM EGTA, 1% Triton X-100, 10% glycerol, protease inhibitor (Roche), and phosphatase inhibitor (Roche) for 20 min. Afterwards, cells were centrifuged for 20 min at 20,000×*g* at 4 °C. After collection of the soluble fraction, mechanical disruption of the insoluble fraction was performed in 6 M urea, 3% SDS, 10% glycerin, and 50 mM Tris pH 6.8. The protein concentration of cell- and tissue lysates (soluble fraction) was determined using a DC protein assay (Bio Rad). Proteins were transferred to a nitrocellulose or PVDF membrane after SDS-PAGE separation.

Protein staining was performed with the antibodies listed in Reagents and tool table.

Proteins were visualised using the FUSION SOLO S Imaging System together with the analysis software Evolution-Capt (Vilber).

### RNA isolation

RNA isolation was performed using the RNeasy® Mini Kit (QIAGEN) according to the manufacturer’s protocol. RNA concentration was measured with a NanoDrop (Thermo Fisher Scientific) (Daoud et al, 2022).

Libraries were prepared from 500 ng of total RNA. Enzymatic depletion of ribosomal RNA was conducted using the Illumina Ribo-Zero Plus rRNA Depletion Kit. Subsequently, the Illumina® Stranded Total RNA Sample Preparation Kit was used for library preparation.

The depleted RNA was fragmented and reverse-transcribed using random hexamer primers. Second-strand synthesis with dUTPs was followed by A-tailing, adapter ligation, and library amplification (12 cycles). Next, library validation and quantification were performed with the Agilent Tape Station, followed by pooling equimolar amounts of the library. The pool was then quantified using the Peqlab KAPA Library Quantification Kit and the Applied Biosystems 7900HT Sequence Detection System. Sequencing was performed on an Illumina NovaSeq6000 sequencing instrument using a PE100 protocol, aiming for 100 million clusters per sample.

### RNA sequencing data processing and analysis

Sequencing reads were first trimmed using the cutadapt tool (version 4.1), followed by alignment to the murine reference transcriptome GRCm38/mm10 with the STAR aligner (version 2.7.0e). Gene expression was quantified with RSEM (version 1.3.1), and gene-level counts were used as expression values. Protein-coding genes with a total count greater than ten across all samples were retained for analysis. Counts were normalised to a scale of 10^4 across all genes and subsequently log-transformed Ln(TPM + 1)) for use in heatmaps and principal component analysis (PCA). For heatmaps, the expression values were further transformed into z-scores.

Differential gene expression analysis was performed using the pyDESeq2 package (version 0.4.3) in Python with default parameters. The integer part of raw RSEM gene-level counts served as input for the pyDESeq2 analysis. Gene set enrichment analysis was conducted using the gseapy Python package (version 1.1.0), focusing on the 50 hallmark gene sets from MSigDB (version 2023.1.Mm). The preranked module was used for gene set enrichment analysis and ranked gene lists were generated based on DESeq2 “stat” values, with all settings kept at their defaults.

### Lipidomics

Levels of sphingolipids (ceramides and sphingomyelins) in mouse testes were determined by Liquid Chromatography coupled to Electrospray Ionization Tandem Mass Spectrometry (LC-ESI-MS/MS) using a procedure previously described (Schull et al, 2015; Schwamb et al, 2012; Shaner et al, 2009) with several modifications:

Approximately 20 mg of mouse testes tissue were homogenised in 300 µl of water using the Precellys 24 Homogenisator (Peqlab) at 6500 rpm for 30 s. The protein content of the homogenate was routinely determined using bicinchoninic acid. To 100 µl of homogenate 500 µl of methanol, 250 µl of chloroform and internal standards (100 pmol d7-Ceramide 15:0 and 124 pmol Sphingomyelin 12:0, Avanti Polar Lipids) were added. The mixture was sonicated for 5 min, and lipids were extracted overnight shaking water bath at 48 °C. Interfering glycerolipids were degraded by alkaline hydrolysis adding 75 µl of 1 M potassium hydroxide in methanol. After 5 min of sonication, the extract was incubated for 2 h at 37 °C, and then neutralised with 6 µl of glacial acetic acid. 2 ml of chloroform and 4 ml of water were added to the extract which was vortexed vigorously for 30 s and then centrifuged (4000×*g*, 5 min, 4 °C) to separate layers. The lower (organic) phase was transferred to a new tube, and the upper phase extracted with additional 2 ml of chloroform. The combined organic phases were dried under a stream of nitrogen. The residues were resolved in 300 µl of mobile phase solvent A (see below) and sonicated for 5 min. After centrifugation (12,000×*g*, 5 min, 4 °C), 50 µl of the clear supernatants were transferred to autoinjector vials.

LC-MS/MS analysis was performed by injecting 20 µl sample onto a normal phase Nucleosil NH_2_ column (50 mm × 2 mm ID, 3 µm particle size, 120 Å pore size, Macherey-Nagel) at 20 °C and with detection using a QTRAP 6500 triple quadrupole/linear ion trap mass spectrometer (SCIEX). The LC (1260 Infinity Binary LC System, Agilent) was operated at a flow rate of 0.75 ml/min with a mobile phase of acetonitrile/methanol/acetic acid 97:2:1 (v/v/v), with 5 mM ammonium acetate (solvent A) and methanol/acetic acid 99:1 (v/v/v) with 5 mM ammonium acetate (solvent B). Sphingolipid species were eluted with the following gradient: initial, 0% B; 0.5 min, 0% B; 0.7 min, 10% B; 1.2 min, 10% B; 1.6 min, 18% B; 2.2 min, 18% B; 2.6 min, 100% B; 4.5 min, 100% B; 4.9 min, 0% B; 6.5 min, 0% B. Sphingolipid species were monitored in the positive ion mode with their specific Multiple Reaction Monitoring (MRM) transitions (Ref. 1). The instrument settings for nebulizer gas (Gas 1), turbogas (Gas 2), curtain gas, and collision gas were 60 psi, 80 psi, 40 psi, and medium, respectively. The interface heater was on, the Turbo V ESI source temperature was 300 °C, and the ionspray voltage was 5.5 kV. For the MRM transitions of ceramides, the values for declustering potential, entrance potential, and cell exit potential were 80 V, 10 V, and 8 V, and for the sphingomyelins 110 V, 10 V, and 6 V. The collision energies ranged from 35 to 45 V (sphingomyelins) and 39 to 47 V (sphingomyelins), respectively. The LC chromatogram peaks of endogenous and standard sphingolipids were integrated using the MultiQuant 3.0.3 software (AB SCIEX). Ceramide and sphingomyelin species were quantified on the basis of calibration curves which were calculated from LC-MS/MS measurements of serially diluted synthetic ceramide and sphingomyelins standards (Avanti Polar Lipids) in solvent A. To each dilution fixed amounts of the internal standards were added. The standard calibration curves were plotted based on molar concentration versus peak area ratio of the endogenous sphingolipid to the respective internal standard. The calculated amounts of endogenous ceramides and sphingomyelins were normalised to the protein content of the sample.

### ASM enzymatic assay

ASM enzymatic activity was assessed by using [N-methyl-14C]Sphingomyelin (56 mCi mmol-1) and counting released [14 C]phosphorylcholine using a β-counter as previously described (Haubert et al, 2007; Kashkar et al, 2005).

### Transmission electron microscopy (TEM)

Epididymides were bisected and immersion-fixed in 2% formaldehyde (Science Services) and 2% glutaraldehyde (Sigma) in 0.1 M sodium cacodylate buffer (Applichem) for 48 h. Samples were washed four times with 0.1 M sodium cacodylate buffer. Post-fixation was performed with 2% OsO4 (Science Services) in 0.1 M cacodylate buffer for 2 h at 4 °C. Samples were washed four times with 0.1 M cacodylate buffer and dehydrated using an ascending ethanol series (50%, 70%, 90%, 3×100%) for 20 min each. After incubation with a mix of 50% (v/v) ethanol/propylenoxide and two times with pure propylenoxide for 20 min each step, samples were infiltrated with a mixture of 50% (v/v) epon/propylenoxide for 5 h and 70% (v/v) epon/propylenoxide overnight each at 4 °C. The next day samples were transferred into fresh pure epon (Science Services) and incubated 8 h at 4 °C before adding another pure epon step overnight at 4 °C. The next day, epon was exchanged and samples were incubated for 2 h at room temperature, placed into PELCO 21-cavity embedding mold (Plano, 10505) and cured for 72 h at 60 °C. Ultrathin sections (70 nm) were cut from the position where the seminal duct is connected to the epididymis using an ultramicrotome (Leica Microsystems, UC6) and a 45° diamond knife (Diatome, Biel, Switzerland). Sections were stained with 1.5% uranyl acetate (Agar Scientific) for 15 min at 37 °C and with 3% Reynolds lead citrate solution made from Lead (II), nitrate (Roth), and tri-Sodium citrate dehydrate (Roth) for 4 min. Images were acquired using a JEM-2100Plus Transmission Electron Microscope (JEOL) operating at 80 kV equipped with a OneView 4 K camera (Gatan).

### Scanning electron microscopy (SEM)

For SEM analysis of sperm, extracted sperm was fixed in 2% formaldehyde (Science Services), 2% glutaraldehyde (Sigma) in 0.1 M sodium cacodylate buffer (Applichem) for 24 h. A drop of sample was put onto a wafer piece (Plano) which were glow discharged before using a Leica EM ACE 200 (5 s, 10 mA). Samples were dehydrated using an ascending ethanol series (50%, 70%, 90%, 100%) for 3 min and incubated with Hexamethyldisilazane (HMDS) for 3 min each. HMDS was removed and samples were dried under the hood. Samples were sputter coated with gold and imaged using a Zeiss Neon 40 Cross Beam operating at 5 kV.

For SEM analysis of MEFs, cells were seeded on glass coverslips. On the following day, cells were treated with 20 µM of S-63845 and ABT737. After treatment of the indicated time points, cells were fixed for 1 h in 2% Glutaraldehyde (Sigma) with 2.5% Sucrose (Roth) and 3 mM CaCl_2_ (Sigma-Aldrich) in 0.1 M HEPES buffer (Sigma-Aldrich) pH 7.4. Samples were washed 3 times with 0.1 M HEPES buffer and incubated with 1% Osmium tetroxide (Science Services) and 1% potassium hexacyanoferrate (Sigma-Aldrich) for 1 h at 4 °C. After three times 5 min wash with 0.1 M Cacodylate buffer (Applichem), samples were dehydrated at 4 °C using ascending ethanol series (50%, 70%, 90%, 3 × 100%) for 7 min each. Samples were incubated with Hexamethyldisilazane (HMDS) (Sigma-Aldrich) for 3 min. HMDS was removed and samples were dried under the hood. SEM images were obtained using a Zeiss Neon 40 microscope with a Schottky field emission gun. The samples were attached on a metal sample holder with conductive carbon adhesive tabs. To avoid electrostatic charging, a 10 nm thick gold layer was deposited via DC-sputtering. The measurements were performed at a chamber pressure ≤1 × 10^−7 ^mbar with an acceleration voltage of 5 kV and a working distance of about 8 mm. SE2 and Inlens detector were used.

### Quantification and statistical analysis

GraphPad Prism for macOS (version 8.2.1; GraphPad Software, LLC) and Microsoft® Excel for Mac (version 16.78; Microsoft) were used for statistical analysis. Data are shown as mean ± s.d. Sample sizes (animals, replicates) are displayed as individual data points in each figure. Experimental results shown are representative of at least three biologically independent experiments.

## Supplementary information


Table EV1
Table EV2
Table EV3
Appendix
Peer Review File
Dataset EV1
Dataset EV2
Dataset EV3
Dataset EV4
Dataset EV5
Dataset EV6
Source data Fig. 1
Source data Fig. 2
Source data Fig. 3
Source data Fig. 4
Source data Fig. 5
Source data Fig. 6
EV Figure Source Data WBs
Expanded View Figures


## Data Availability

The datasets produced in this study are available in the following databases: RNA-Seq data: European Nucleotide Archive at EMBL-EBI PRJEB111758. Lipidomics data: Zenodo Data Repository https://doi.org/10.5281/zenodo.20607257. The source data of this paper are collected in the following database record: biostudies:S-SCDT-10_1038-S44318-026-00871-4.
