# Supplementary material for: Proteolytic activation of executioner caspase-3 and -7 regulates different physiological processes in mice
Source: EMBO J. 2026 Jul 21;45(17):6197–228. doi: 10.1038/s44318-026-00871-4 (PMC13534459; doi:10.1038/s44318-026-00871-4)
Supplement: Supplementary file 1 [file 44318_2026_871_MOESM19_ESM.pdf]

Expanded View Figures

Figure EV1. Mice expressing cleavage-resistant CASP3(D175A) or CASP7(D198A) do not show obvious phenotypic alterations.

Related to Fig. 1. (**A**) Schematic illustration of the *Casp3* and *Casp7* genes with the domain structure and the position of the cleavage side (arrow) with representative sequence analysis of mice with the respective genotypes. (**B**) Representative spleen sections from 50-week-old *WT*, $Casp3^{DA/DA}$, and $Casp7^{DA/DA}$ mice immunostained for cleaved CASP3. Scale bars: 50 µm. (**C**) WB analysis of cytosolic extracts from *WT*, $Casp3^{-/-}$, and $Casp7^{-/-}$ MDFs treated with 1 U recombinant active CASP8 and CASP9. Lysates were analysed for cleavage of the indicated caspases. Results representative of three independent experiments. (**D**) DEVD-AFC hydrolysis measurement using cytosolic extracts (100 µg) from *WT*, $Casp3^{-/-}$, and $Casp7^{-/-}$ MDFs treated with 1 U recombinant active CASP8 or CASP9. Dots represent technical replicates of one experiment, representative of three independent experiments. Data is represented as mean ± s.d. (**E**) Expected and observed numbers of mice per genotype at E13.5, E18.5, day of birth (P0) and weaned, obtained from the indicated crossings. Source data are available online for this figure.

 © The Author(s)

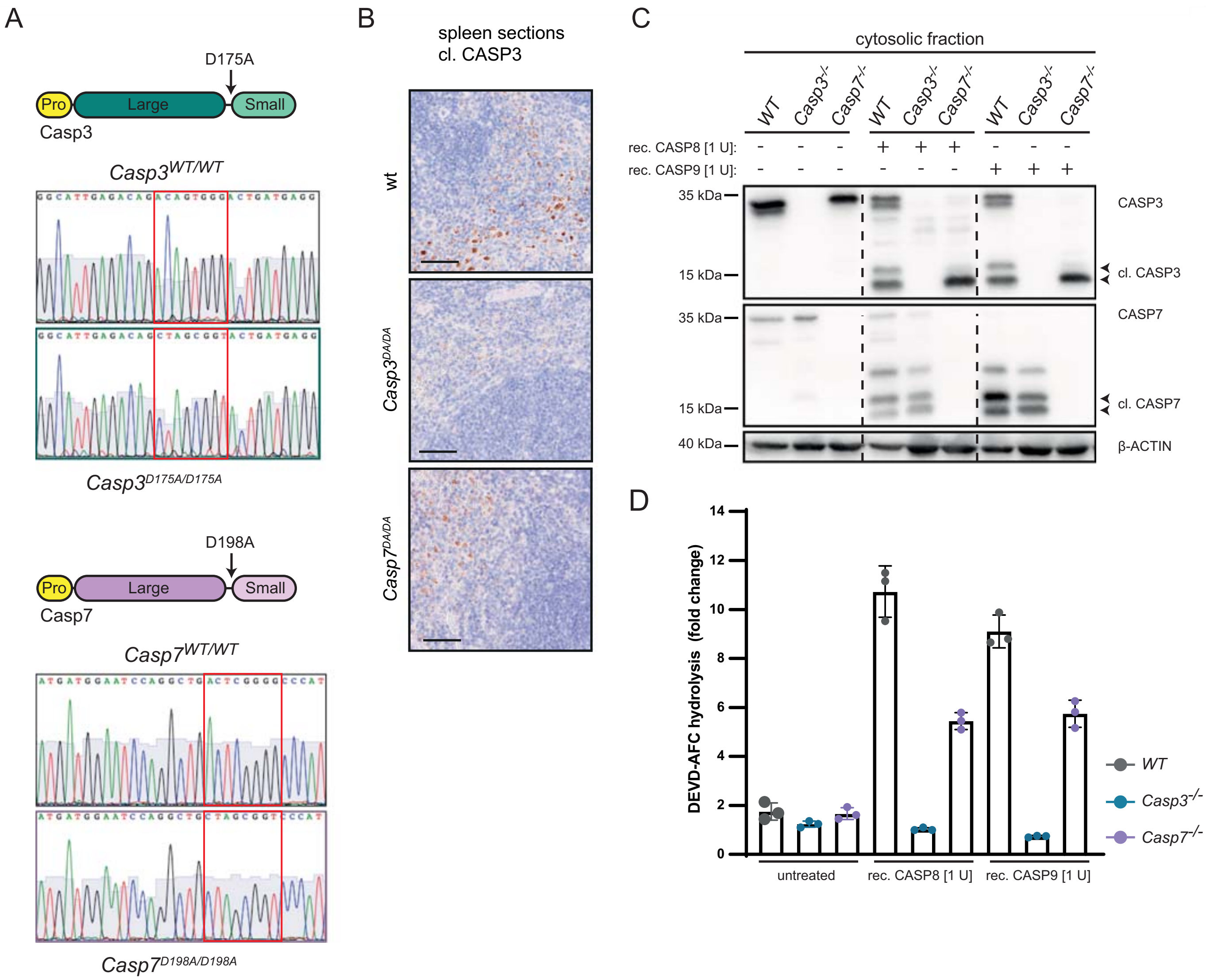

E

$Casp3^{DA/wt}$ $Casp7^{DA/wt}$ x $Casp3^{DA/wt}$ $Casp7^{DA/wt}$								
	E13.5		E18.5		P0		weaned	
**Genotype**	**expected**	**observed**	**expected**	**observed**	**expected**	**observed**	**expected**	**observed**
$Casp3^{wt/wt}$ $Casp7^{wt/wt}$	3.625	4	1.4375	0	2.75	5	5.0625	7
$Casp3^{wt/wt}$ $Casp7^{DA/wt}$	7.25	7	2.875	3	5.5	13	10.125	12
$Casp3^{wt/wt}$ $Casp7^{DA/DA}$	3.625	5	1.4375	2	2.75	4	5.0625	4
$Casp3^{DA/wt}$ $Casp7^{wt/wt}$	7.25	7	2.875	3	5.5	4	10.125	7
$Casp3^{DA/wt}$ $Casp7^{DA/wt}$	14.5	11	5.75	7	11	6	20.25	19
$Casp3^{DA/wt}$ $Casp7^{DA/DA}$	7.25	7	2.875	3	5.5	5	10.125	12
$Casp3^{DA/DA}$ $Casp7^{wt/wt}$	3.625	6	1.4375	1	2.75	5	5.0625	13
$Casp3^{DA/DA}$ $Casp7^{DA/wt}$	7.25	7	2.875	3	5.5	2	10.125	7
$Casp3^{DA/DA}$ $Casp7^{DA/DA}$	3.625	4	1.4375	1	2.75	0	5.0625	0
**Total**	**58**	**58**	**23**	**23**	**44**	**44**	**81**	**81**

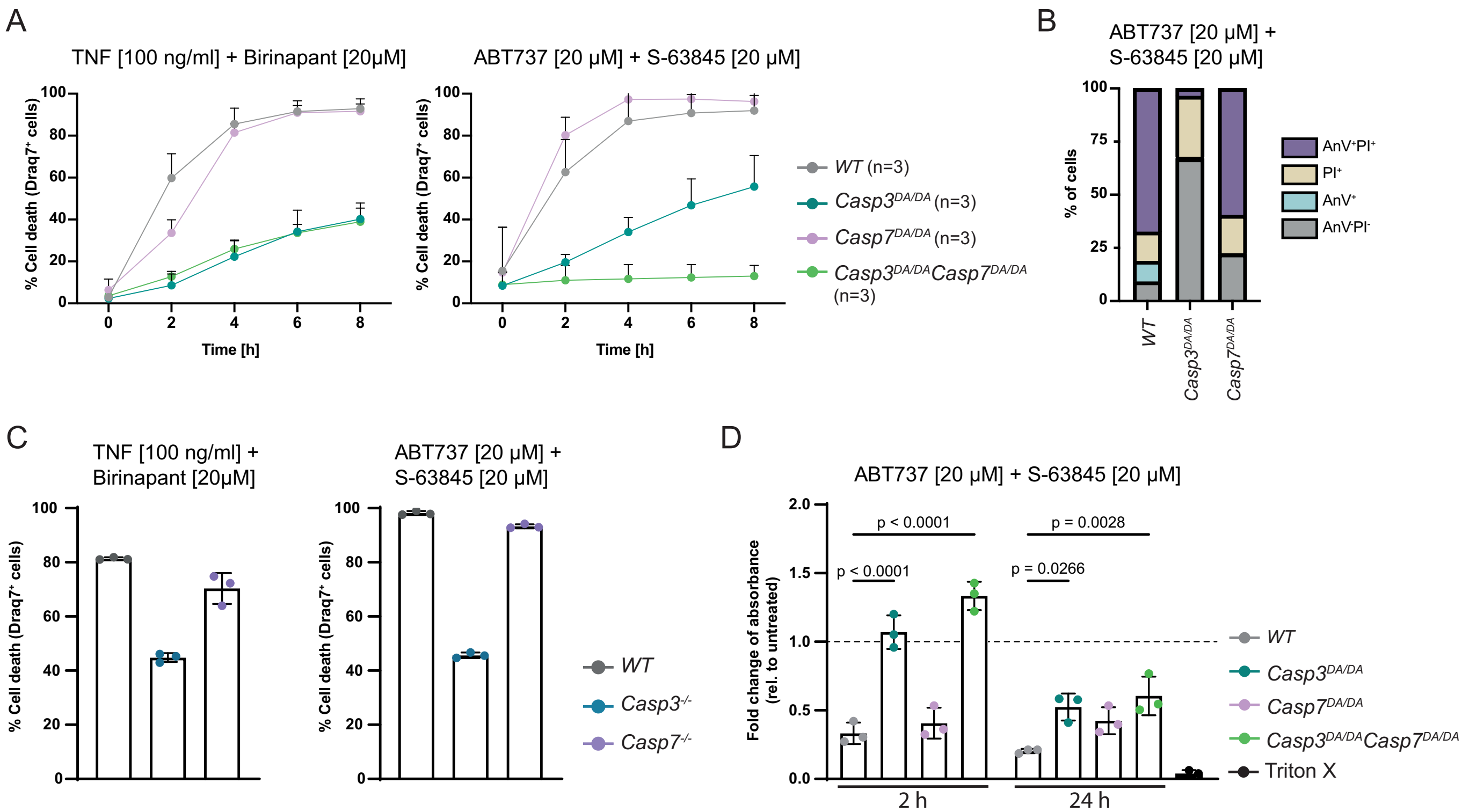

Figure EV2. CASP3 is the major driver of apoptosis.

Related to Fig. 2. (**A**) IncuCyte analysis to determine % cell death of MEFs with the indicated genotypes treated with TNF (100 ng/ml) and birinapant (20 μM) or S-63845 (20 μM) and ABT737 (20 μM) using DRAQ7 dye for the indicated time points. Birinapant was added 1 h prior to treatment. Data are displayed as mean of three independent experiments ± s.d. (**B**) Flow cytometry analysis of MEFs from *WT*, *Casp3*$^{DA/DA}$, and *Casp7*$^{DA/DA}$ mice stained with Annexin V and PI after 1 h treatment with ABT737 and S-63845 in the indicated concentrations. (**C**) IncuCyte analysis to determine % cell death of MDFs using DRAQ7 dye with the indicated genotypes treated with TNF (100 ng/ml) and birinapant (20 μM) or S-63845 (20 μM) and ABT737 (20 μM) for 4 h. Birinapant was added 1 h prior to treatment. Data is displayed as mean of three technical replicates ± s.d. Results representative of three independent experiments. (**D**) Measurement of metabolic activity by CellTiter 96® AQueous One Solution (MTS) of *WT*, *Casp3*$^{DA/DA}$, and *Casp7*$^{DA/DA}$ MEFs, treated with S-63845 (20 μM) and ABT737 (20 μM) for 2 h and 24 h. Fold change of absorbance at 490 nm relative to untreated cells of the same genotype. Dots represent independent experiments ($n = 3$). Cells treated with 0.1% Triton X were used as negative control. Data is represented as mean ± s.d. One-way ANOVA followed by Dunnett's post-analysis compared to the corresponding *WT* values. Exact *P* values: 0.000000943 (*WT* vs *Casp3*$^{DA/DA}$) and 0.000000008 (*WT* vs *Casp3*$^{DA/DA}$ *Casp7*$^{DA/DA}$).

 © The Author(s)

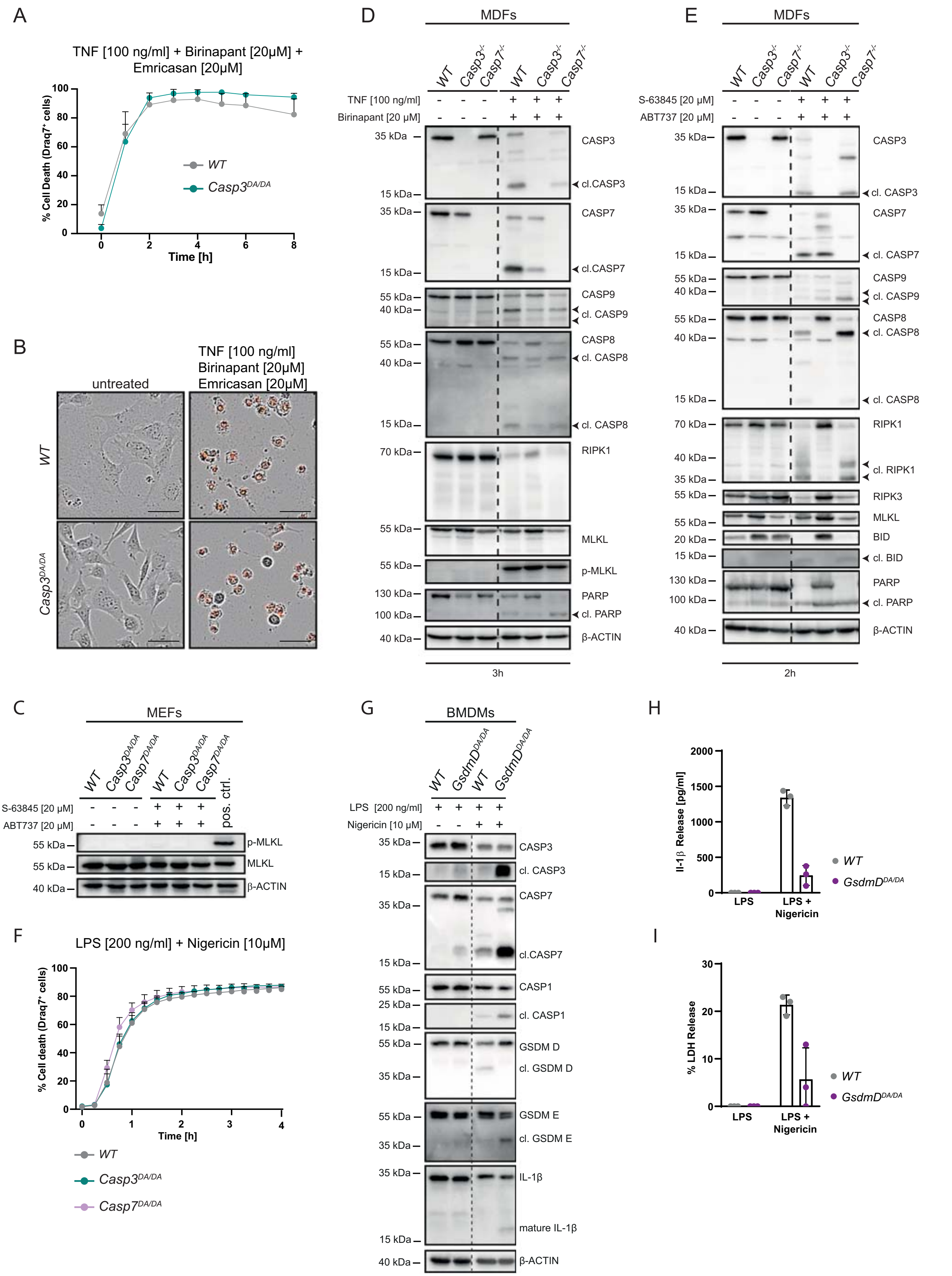
A
TNF [100 ng/ml] + Birinapant [20μM] + Emricasan [20μM]
% Cell Death (Draq7+ cells)
Time [h]
WT
Casp3DA/DA
B
untreated
TNF [100 ng/ml] Birinapant [20μM] Emricasan [20μM]
WT
Casp3DA/DA
C
MEFs
WT
Casp3DA/DA
Casp7DA/DA
pos. ctrl.
S-63845 [20 μM]
ABT737 [20 μM]
p-MLKL
MLKL
β-ACTIN
D
MDFs
WT
Casp3-/-
Casp7-/-
TNF [100 ng/ml]
Birinapant [20 μM]
CASP3
cl.CASP3
CASP7
cl.CASP7
CASP9
cl. CASP9
CASP8
cl. CASP8
RIPK1
MLKL
p-MLKL
PARP
cl. PARP
β-ACTIN
3h
E
MDFs
S-63845 [20 μM]
ABT737 [20 μM]
cl. CASP3
cl. CASP7
RIPK1
cl. RIPK1
RIPK3
BID
cl. BID
2h
F
LPS [200 ng/ml] + Nigericin [10μM]
% Cell death (Draq7+ cells)
Time [h]
Casp7DA/DA
G
BMDMs
GsdmDDA/DA
LPS [200 ng/ml]
Nigericin [10 μM]
CASP1
cl. CASP1
GSDM D
cl. GSDM D
GSDM E
cl. GSDM E
IL-1β
mature IL-1β
H
Il-1β Release [pg/ml]
LPS
LPS + Nigericin
I
% LDH Release

© The Author(s)

Figure EV3. Activation of CASP3 is important for proteolytic processing of the majority of apoptotic substrates.

Related to Fig. 3. (**A**) IncuCyte analysis to determine % cell death of MEFs using DRAQ7 dye with the indicated genotypes treated with TNF (100 ng/ml), birinapant (20 µM), and emricasan (20 µM) at the indicated time points. Birinapant was added 1 h prior to treatment. Data is displayed as mean of three technical replicates ± s.d. Results representative of three independent experiments. (**B**) Representative images of MEFs with the indicated genotype treated with TNF (100 ng/ml), birinapant (20 µM), and emricasan (20 µM) for 1 h obtained by IncuCyte. Results representative of three independent experiments. Scale bars: 100 µm. (**C**) WB analysis of MLKL phosphorylation of lysates in Fig. 3B including a positive control: *WT* MEFs treated with TNF (100 ng/ml), birinapant (20 µM), and emricasan (20 µM). (**D**) WB analysis of MDFs with the indicated genotypes treated with TNF (100 ng/ml) and birinapant (20 µM) for 3 h. Birinapant was added 1 h prior to treatment. Lysates were analysed for the expression of the indicated proteins. Results representative of three independent experiments. (**E**) WB analysis of MDFs treated with S-63845 (20 µM) and ABT737 (20 µM) for 2 h. Lysates were analysed for the expression of the indicated proteins. Results representative of three independent experiments. (**F**) IncuCyte analysis to determine % cell death of bone marrow derived macrophages (BMDMs) using DRAQ7 dye with the indicated genotypes treated with LPS (200 ng/ml) and nigericin (10 µM) at the indicated time points. LPS was added 4 h prior to treatment. Data is displayed as mean of three technical replicates ± s.d. (**G**) WB analysis of *WT* BMDMs and BMDMs expressing cleavage-resistant Gasdermin D(D276A) treated with LPS (200 ng/ml) alone or together with nigericin (10 µM) for 2 h. LPS was added 4 h prior to treatment. Lysates were analysed for the expression of the indicated proteins. Results representative of three independent experiments. (**H**) IL-1β release measured by ELISA in cell culture supernatant of BMDMs from *WT* ($n = 3$) and $GsdmD^{DA/DA}$ ($n = 3$) mice as treated in (**G**). Dots represent biological replicates. Data are represented as mean ± s.d. (**I**) LDH release of BMDMs in cell culture supernatant of BMDMs from *WT* ($n = 3$) and $GsdmD^{DA/DA}$ ($n = 3$) mice as treated in (**G**). Dots represent technical replicates. Data are represented as mean ± s.d. Source data are available online for this figure.

© The Author(s)

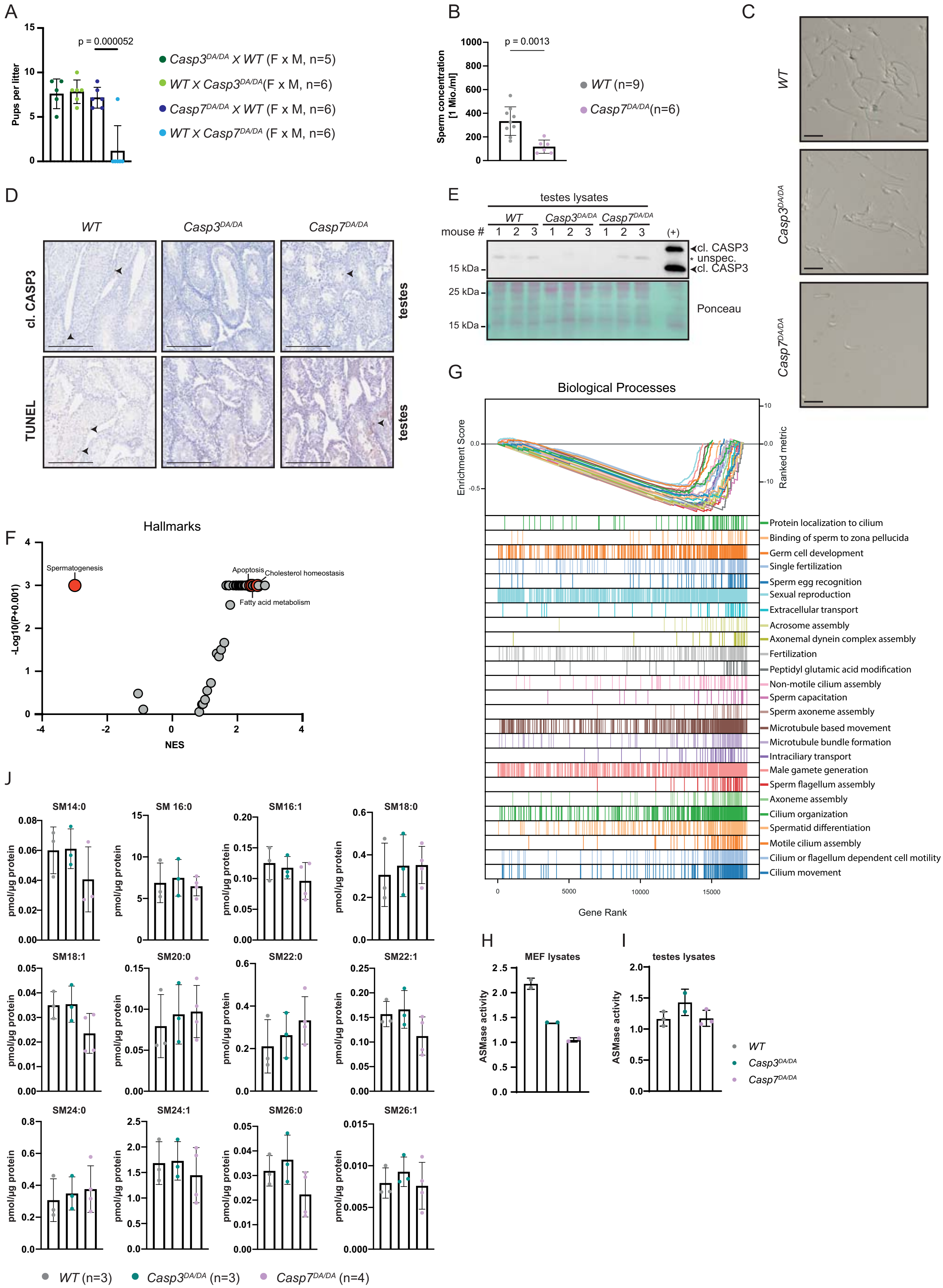
A
Pups per litter
p = 0.000052
Casp3DA/DA X WT (F x M, n=5)
WT X Casp3DA/DA(F x M, n=6)
Casp7DA/DA X WT (F x M, n=6)
WT X Casp7DA/DA (F x M, n=6)
B
Sperm concentration [1 Mio./ml]
p = 0.0013
WT (n=9)
Casp7DA/DA(n=6)
C
WT
Casp3DA/DA
Casp7DA/DA
D
WT
Casp3DA/DA
Casp7DA/DA
cl. CASP3
TUNEL
testes
E
testes lysates
WT
Casp3DA/DA
Casp7DA/DA
mouse #
1 2 3 1 2 3 1 2 3
(+)
cl. CASP3
* unspec.
cl. CASP3
15 kDa
25 kDa
15 kDa
Ponceau
F
Hallmarks
Spermatogenesis
Apoptosis
Cholesterol homeostasis
Fatty acid metabolism
-Log10(P+0.001)
NES
G
Biological Processes
Enrichment Score
Ranked metric
Protein localization to cilium
Binding of sperm to zona pellucida
Germ cell development
Single fertilization
Sperm egg recognition
Sexual reproduction
Extracellular transport
Acrosome assembly
Axonemal dynein complex assembly
Fertilization
Peptidyl glutamic acid modification
Non-motile cilium assembly
Sperm capacitation
Sperm axoneme assembly
Microtubule based movement
Microtubule bundle formation
Intraciliary transport
Male gamete generation
Sperm flagellum assembly
Axoneme assembly
Cilium organization
Spermatid differentiation
Motile cilium assembly
Cilium or flagellum dependent cell motility
Cilium movement
Gene Rank
H
MEF lysates
ASMase activity
I
testes lysates
ASMase activity
WT
Casp3DA/DA
Casp7DA/DA
J
SM14:0
SM 16:0
SM16:1
SM18:0
SM18:1
SM20:0
SM22:0
SM22:1
SM24:0
SM24:1
SM26:0
SM26:1
pmol/µg protein
WT (n=3)
Casp3DA/DA (n=3)
Casp7DA/DA (n=4)

© The Author(s)

Figure EV4. *Casp7$^{DA/DA}$* male mice are infertile and show altered spermatogenesis.

Related to Fig. 4. (**A**) Observed pups per litter obtained from the indicated crossings. Data are mean ± s.d. One-way ANOVA with Sidak's post-analysis test. (**B**) Quantification of sperm concentration [1 Mio/ml] in *WT* ($n = 9$) and *Casp7$^{DA/DA}$* ($n = 6$) mice at 12–30 weeks of age, measured using a Neubauer chamber. Data are mean ± s.d. Unpaired *t* test was performed. (**C**) Representative images of sperm from *WT*, *Casp3$^{DA/DA}$*, and *Casp7$^{DA/DA}$* mice at 12–30 weeks of age. Scale bars = 10 µm. (**D**) Representative images of testes of *WT*, *Casp3$^{DA/DA}$*, and *Casp7$^{DA/DA}$* mice at 30 weeks of age immunostained for cl. CASP3 and stained for DNA fragmentation by TUNEL assay. Scale bars: 0.25 mm. Arrows indicate positively stained cells. (**E**) Control WB for testes lysates from Fig. 4F, showing specific bands for cl. CASP3 in MEFs treated with S-63845 (20 µM) and ABT737 (20 µM) for 2 h (+). Asterix indicates unspecific band. (**F**) NES derived from GSEA comparing testes from *WT* ($n = 3$) and *Casp7$^{DA/DA}$* mice ($n = 3$), using the hallmark gene set database. Significance was tested using default settings of the gseapy Python package (version 1.1.0), based on random permutation. (**G**) Enrichment plots of pathways (biological processes) involved in the male reproductive system, significantly downregulated in *Casp7$^{DA/DA}$* ($n = 3$) vs *WT* mice ($n = 3$). (**H**) ASMase activity, measured in lysates from *WT*, *Casp3$^{DA/DA}$*, and *Casp7$^{DA/DA}$* MEFs. Dots represent independent experiments. Data are mean ± s.d. (**I**) ASMase activity, measured in lysates from *WT* ($n = 3$), *Casp3$^{DA/DA}$* ($n = 2$), and *Casp7$^{DA/DA}$* ($n = 3$) testes. Dots represent individual mice. Data are mean ± s.d. (**J**) Concentration of different sphingomyelins [pmol/µg protein] in testes from *WT* ($n = 3$), *Casp3$^{DA/DA}$* ($n = 3$), and *Casp7$^{DA/DA}$* ($n = 4$) mice at 30 weeks of age. Data are mean ± s.d. One-way analysis of variance (ANOVA) followed by Dunnett's post-analysis. Source data are available online for this figure.

 © The Author(s)

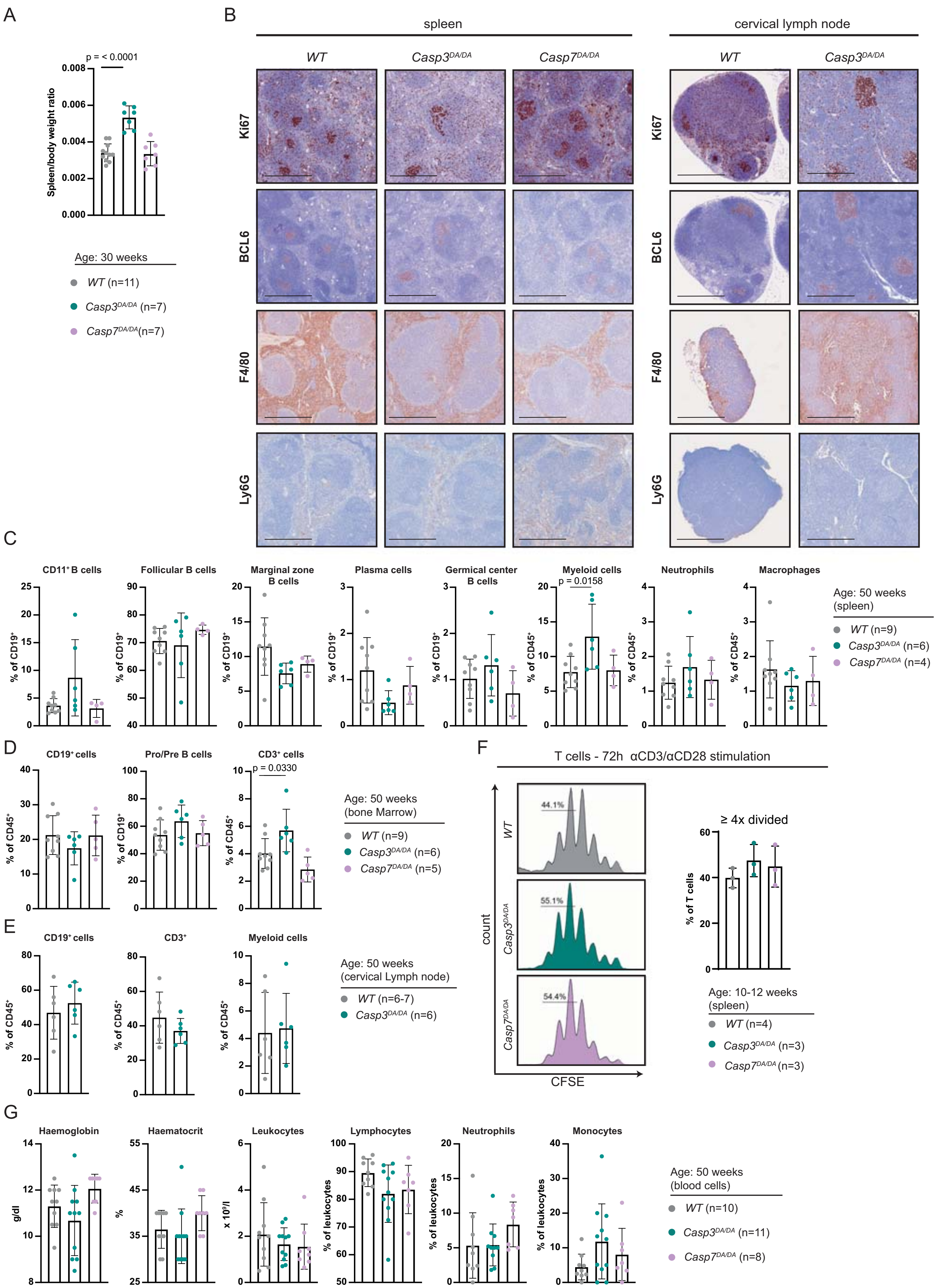
A
B
spleen
cervical lymph node
WT
Casp3DA/DA
Casp7DA/DA
Ki67
BCL6
F4/80
Ly6G
p = < 0.0001
Spleen/body weight ratio
Age: 30 weeks
WT (n=11)
Casp3DA/DA (n=7)
Casp7DA/DA(n=7)
C
CD11+ B cells
Follicular B cells
Marginal zone B cells
Plasma cells
Germical center B cells
Myeloid cells
Neutrophils
Macrophages
p = 0.0158
% of CD19+
% of CD45+
Age: 50 weeks (spleen)
WT (n=9)
Casp3DA/DA (n=6)
Casp7DA/DA (n=4)
D
CD19+ cells
Pro/Pre B cells
CD3+ cells
p = 0.0330
Age: 50 weeks (bone Marrow)
WT (n=9)
Casp3DA/DA (n=6)
Casp7DA/DA (n=5)
E
CD19+ cells
CD3+
Myeloid cells
Age: 50 weeks (cervical Lymph node)
WT (n=6-7)
Casp3DA/DA (n=6)
F
T cells - 72h αCD3/αCD28 stimulation
44.1%
55.1%
54.4%
count
CFSE
≥ 4x divided
% of T cells
Age: 10-12 weeks (spleen)
WT (n=4)
Casp3DA/DA (n=3)
Casp7DA/DA (n=3)
G
Haemoglobin
Haematocrit
Leukocytes
Lymphocytes
Neutrophils
Monocytes
g/dl
%
x 10⁹/l
% of leukocytes
Age: 50 weeks (blood cells)
WT (n=10)
Casp3DA/DA (n=11)
Casp7DA/DA (n=8)

Figure EV5. *Casp3$^{DA/DA}$* mice develop selective hyperplasia of lymphoid tissues.

Related to Fig. 5. (**A**) Spleen/body weight ratio of 30-week-old mice. Dots represent individual mice. Data are mean ± s.d. One-way ANOVA with Dunnett's post-analysis test compared to the corresponding *WT* values. (**B**) Representative spleen sections from 50-week-old *WT*, *Casp3$^{DA/DA}$*, and *Casp7$^{DA/DA}$* mice and representative cervical lymph node sections from 50-week-old *WT* and *Casp3$^{DA/DA}$* mice immunostained for Ki67, BCL6, F4/80, and Ly6G. Scale bars: 1.0 mm (spleen) and 0.5 mm (cervical lymph node). (**C**) Flow cytometry analysis of immune cell populations in spleens from 50-week-old *WT* ($n = 9$), *Casp3$^{DA/DA}$* ($n = 6$), and *Casp7$^{DA/DA}$* ($n = 4$) mice. Dots represent individual mice. Data are mean ± s.d. One-way ANOVA with Dunnett's post-analysis test compared to the corresponding *WT* values. (**D**) Flow cytometry analysis of immune cell populations in bone marrows from 50-week-old *WT* ($n = 9$), *Casp3$^{DA/DA}$* ($n = 6$), and *Casp7$^{DA/DA}$* ($n = 5$) mice. Data are mean ± s.d. One-way ANOVA with Dunnett's post-analysis test compared to the corresponding *WT* values. (**E**) Flow cytometry analysis of immune cell populations in cervical lymph nodes from 50-week-old *WT* ($n = 6$) and *Casp3$^{DA/DA}$* ($n = 6$) mice. Data are mean ± s.d. Significance was tested by unpaired *t* test. (**F**) T cells, isolated from spleens of 10-12-week-old *WT* ($n = 3$), *Casp3$^{DA/DA}$* ($n = 3$), and *Casp7$^{DA/DA}$* ($n = 3$) mice, were labelled with CFSE proliferation dye and stimulated with anti-CD3/CD28 antibodies for 72 h. CFSE signal was measured on a Gallios flow cytometer. Top: representative histograms of CFSE signal, bottom: percent of T cells that divided ≥4 times. Dots represent individual mice. Data is presented as mean ± s.d. One-way ANOVA with Dunnett's post-analysis test compared to the *WT* value. (**G**) Analysis of cardiac blood from 50-week-old *WT* ($n = 10$), *Casp3$^{DA/DA}$* ($n = 11$), and *Casp7$^{DA/DA}$* ($n = 6$) for haemoglobin concentration, haematocrit, and immune cell numbers. Dots, individual mice. Data are mean ± s.d. One-way ANOVA with Dunnett's post-analysis test compared to the corresponding *WT* values.

 © The Author(s)
