# Supplementary material for: Proteolytic activation of executioner caspase-3 and -7 regulates different physiological processes in mice
Source: EMBO J. 2026 Jul 21;45(17):6197–228. doi: 10.1038/s44318-026-00871-4 (PMC13534459; doi:10.1038/s44318-026-00871-4)
Supplement: Supplementary file 2 [file 44318_2026_871_MOESM4_ESM.pdf]

APPENDIX for:

Proteolytic activation of executioner caspase-3 and -7 regulates different physiological processes in mice

Table of contents

Appendix Figure S1 ………………………………………………………….. page 2

Appendix Figure S2 ………………………………………………………….. page 3

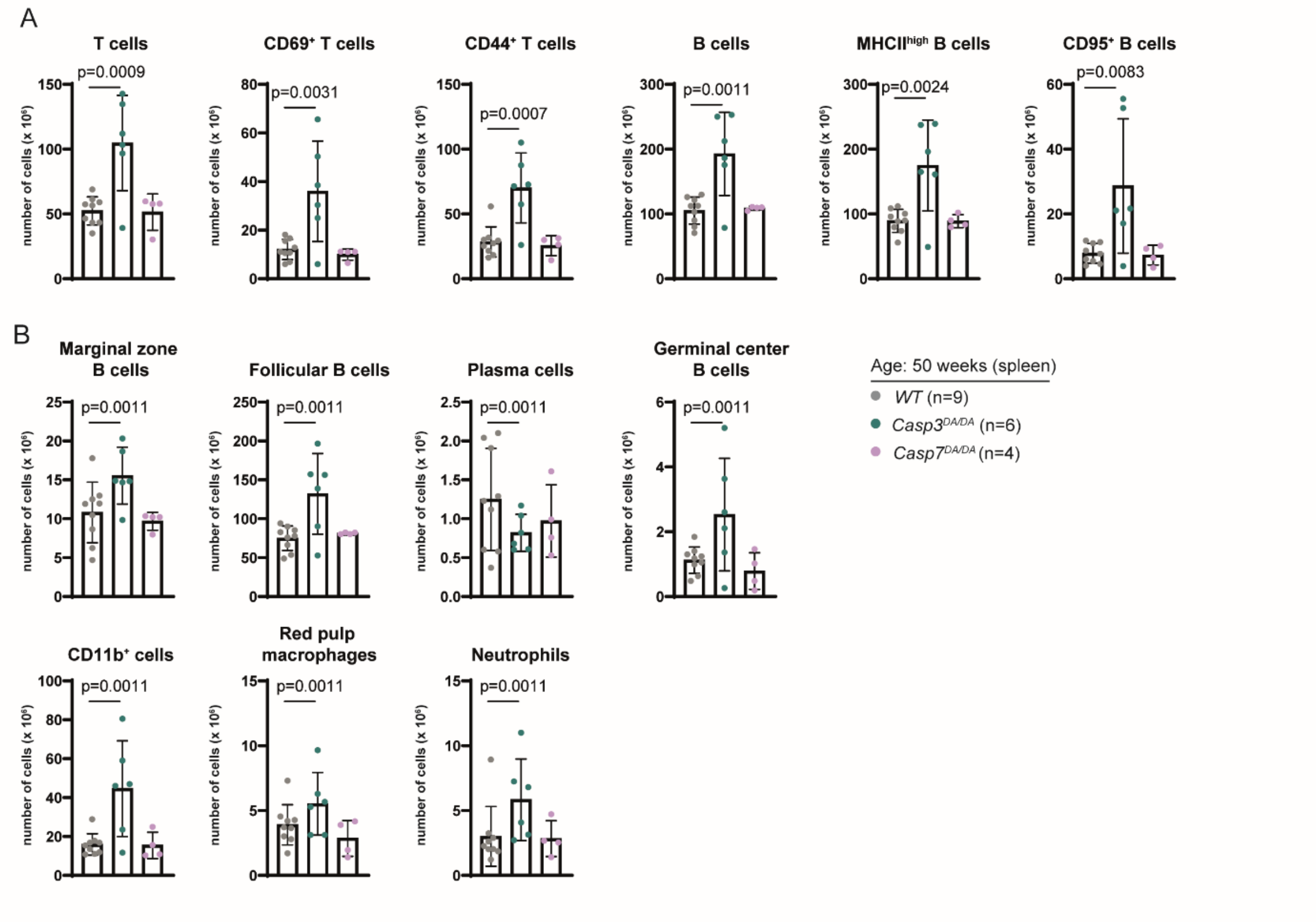

Appendix Figure S1: Absolute cell counts of leukocytes in spleens of *WT*, *Casp3*$^{DA/DA}$ and *Casp7*$^{DA/DA}$ mice.

(**A)** Absolute counts of flow cytometry analysis of Fig. 5D, analysing indicated immune cell populations in spleens from spleens of 50-week-old *WT* (n=9), *Casp3*$^{DA/DA}$ (n=6), and *Casp7*$^{DA/DA}$ (n=4) mice. Dots represent individual mice. Data are mean ± s.d. One-way ANOVA with Dunnett's post-analysis test compared to the corresponding *WT* values. **(B)** Absolute counts of flow cytometry analysis of Fig. EV5C, analysing indicated immune cell populations in spleens from 50-week-old *WT* (n=9), *Casp3*$^{DA/DA}$ (n=6), and *Casp7*$^{DA/DA}$ (n=4) mice. Dots represent individual mice. Data are mean ± s.d. One-way ANOVA with Dunnett's post-analysis test compared to the corresponding *WT* values.

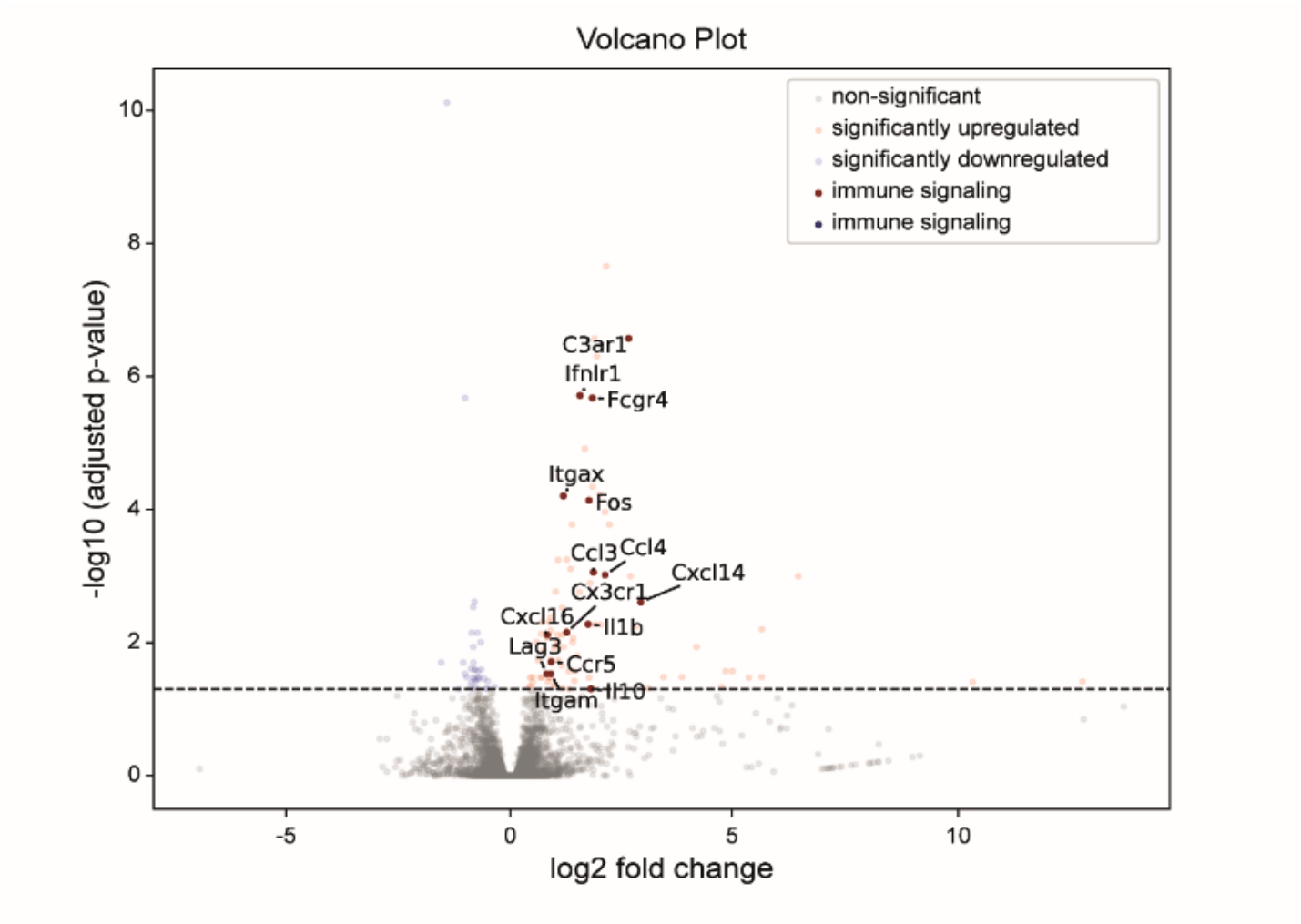

Appendix Figure S2. *Casp3$^{DA/DA}$* mice develop a chronic perivascular inflammation resembling STING-associated inflammatory disorder. Related to Figure 6

Volcano plots displaying significantly upregulated (red) and downregulated (blue) genes in *Casp3$^{DA/DA}$* (n=3) compared to *WT* mice (n=3) (adj. p-value ≥ 0.05, log2FoldChange ≤ -0.7 or ≥ 0.7). Selected genes involved in immune signalling are highlighted.
