# Supplementary material for: Proteolytic activation of executioner caspase-3 and -7 regulates different physiological processes in mice
Source: EMBO J. 2026 Jul 21;45(17):6197–228. doi: 10.1038/s44318-026-00871-4 (PMC13534459; doi:10.1038/s44318-026-00871-4)
Supplement: Supplementary file 3 [file 44318_2026_871_MOESM5_ESM.pdf]

THE EMBO JOURNAL

Proteolytic activation of executioner caspase-3 and -7 regulates different physiological processes in mice

Noëlle Sieg, Johanna Stachelscheid, Besarta Thaqi, Tanja Schwab, Fabian Schorn, Daniela Hahn, Adrian Simon, Yuri Tolkach, Nima Abedpour, Simon Tröder, Ingo Voigt, Felix Gaedke, Katja Wiegmann, Susanne Brodesser, Astrid Schauss, Branko Zevnik, Martin Krönke, Reinhard Buettner, Alexander Quaas, Michael Hallek, Melanie Fritsch, and Hamid Kashkar

Corresponding author(s): Hamid Kashkar (h.kashkar@uni-koeln.de)

Review Timeline:

Submission Date:	11th Aug 25
Editorial Decision:	14th Oct 25
Revision Received:	30th Apr 26
Editorial Decision:	28th May 26
Revision Received:	12th Jun 26
Accepted:	19th Jun 26

Editor: Ioannis Papaioannou

Transaction Report:

(Note: With the exception of the correction of typographical or spelling errors that could be a source of ambiguity, letters and reports are not edited. Depending on transfer agreements, referee reports obtained elsewhere may or may not be included in this compilation. Referee reports are anonymous unless the Referee chooses to sign their reports.)

Dear Prof. Kashkar,

Thank you again for the submission of your manuscript EMBOJ-2025-122134 for consideration by The EMBO Journal, and for your patience during peer review. I would like to sincerely apologize for the rather slow process -for the standards of our journal- on this occasion, but we have now received a complete set of detailed and constructive comments from four referees who reviewed your manuscript (included below).

As you will see, referee #3 recognizes that the manuscript provides a useful genetic resource and uncovers non-redundant roles of caspases-3 and 7 in vivo, but provides only limited mechanistic insight and is rather confirmatory. The other three referees are more positive, indicate interest in the study, and overall find the results novel and relevant, while they also recognize that the work has been well-performed and is for the most part convincing. Having said that, all referees identify a number of limitations and provide constructive suggestions for strengthening the manuscript further, providing further mechanistic insights, and increasing the impact of the work on the field.

On balance, and in light of the referees' comments and recommendations, I would like to invite you to submit a thoroughly revised version of your manuscript taking the referees' suggestions on board, along with a detailed point-by-point response addressing all referees' comments. Please note that it is The EMBO Journal policy to allow only a single round of major revision, and acceptance of your manuscript will therefore depend on the completeness of your responses in this revised version. Given the nature of the raised concerns, it would be useful to discuss your revision plan already during the initial phase of your revision. You are very welcome to share with me a draft point-by-point response letter/revision plan explaining if there are any points you do not agree with or cannot address, or alternatively we could arrange a video call, if you prefer.

We generally allow three months as standard revision time (January 13, 2025). As a matter of policy, competing manuscripts published during this period will not negatively impact our assessment of the conceptual advance presented by your study. However, we request that you contact us as soon as possible upon publication of any related work, to discuss how to proceed. Should you foresee a problem in meeting this three-month deadline, please let us know in advance and we will be able to grant an extension.

Please let me know if you have any questions or comments that you would like to discuss with me. Thank you for the opportunity to consider your work for publication in The EMBO Journal. I look forward to your revision.

Best regards,

Ioannis

Ioannis Papaioannou, PhD
Editor, The EMBO Journal
i.papaioannou@embojournal.org

*****
Instructions for preparing your revised manuscript
*****

1. When you are ready to submit the revision, please upload:

- A Word file of the manuscript text (including legends of main Figures, EV Figures and Tables). Please make sure that changes are highlighted (or "tracked") to be clearly visible.

- Individual production-quality figure files (one file per figure). When assembling your figures, please refer to our figure preparation guidelines in order to ensure proper formatting and readability in print as well as on screen:
https://bit.ly/EMBOPressFigurePreparationGuideline
If the data shown in a figure are obtained from n {less than or equal to} 2, please use scatter plots showing the individual data points.
Please also include scale bars in all microscopy images.
The following points must be specified in each figure legend:
i. the name of the statistical test used to generate error bars and P values
ii. the number (n) of independent experiments (please specify technical or biological replicates) underlying each data point (discussion of statistical methodology can be reported in the Materials and Methods section, but figure legends should contain a basic description of n, P, and the test applied)

iii. the nature of the bars and error bars (s.d., s.e.m.).
See also guidelines for figure legends: https://www.embopress.org/page/journal/14602075/authorguide#figureformat

- A point-by-point response to the referees' comments, with a detailed description of the changes made (as a word file). All referees' concerns must be fully addressed and their suggestions taken on board. When preparing your letter of response to the referees' comments, please bear in mind that this will form part of the Review Process File and will therefore be available online to the community. Please note that you have the possibility to opt out of the transparent process at any stage prior to publication by letting the editorial office know (contact@embojournal.org); if you do opt out, the Review Process File link will point to the following statement: "No Review Process File is available with this article, as the authors have chosen not to make the review process public in this case.". For more details on our Transparent Editorial Process, please visit our website: https://www.embopress.org/page/journal/14602075/authorguide#transparentprocess

- Expanded View (EV) files (replacing Supplementary Information) that are collapsible/expandable online. A maximum of 5 EV Figures can be typeset. EV Figures should be cited as "Figure EV1, Figure EV2" etc. in the text, and their respective legends should be included in the manuscript file after the legends of regular figures. See detailed instructions regarding Expanded View files here:
https://www.embopress.org/page/journal/14602075/authorguide#expandedview

- For the figures that you do NOT wish to display as Expanded View figures, they should be bundled together with their legends in a single PDF file called "Appendix", which should start with a short Table of Contents (including page numbers). Appendix figures should be referred to in the main text as: "Appendix Figure S1, Appendix Figure S2" etc. Please see detailed instructions here: https://www.embopress.org/page/journal/14602075/authorguide#expandedview

- A complete author checklist, which you can download from our author guidelines (https://www.embopress.org/page/journal/14602075/authorguide). Please note that the checklist will also be part of the Review Process File.

2. Please note that no statistics should be calculated and shown in Figures if n=2. Please also note that each p value should be reported as an exact value.

3. Before submitting your revision, primary datasets (and computer code, where appropriate) produced in this study need to be deposited in appropriate public databases (see https://www.embopress.org/page/journal/14602075/authorguide#dataavailability). In particular, all datasets such as DNA or RNA sequencing data, or mass spectrometry data generated in the study must be deposited in appropriate repositories. The accession numbers, database, and the specific URLs (links) should be listed in a formal "Data availability" section (placed after Methods), following the example below:

"The RNA-seq datasets produced in this study are available in the following database:
Gene Expression Omnibus GSE46843 (https://www.ncbi.nlm.nih.gov/geo/query/acc.cgi?acc=GSE46843)"

*** All links should resolve to a page where the data can be accessed. ***

*** Please remember to provide in the Data availability section of your revised manuscript reviewer passwords if the datasets are not yet public. ***

*** The Data Availability Section is restricted to new primary data that are part of this study. In case you have no data that require deposition in a public database, please state so instead of referring to the database: "Our study includes no data deposited in public repositories." under the heading "Data availability". ***

4. The materials and methods need to be described in the manuscript using our structured methods format, which is now required for all research articles. According to this format, the Methods section includes a single "Reagents and Tools Table" - listing key reagents, experimental models, software and relevant equipment including their sources and relevant identifiers- followed by a "Methods and Protocols" section describing the methods. Please download and fill our Reagents and Tools Table template (.docx), which you can find in our author guide: https://www.embopress.org/page/journal/14602075/authorguide#structuredmethods. When submitting your revised manuscript, please do not include the Reagents and Tools Table in the Methods section of the manuscript but instead upload it as a separate file choosing the file type "Reagent Table".

5. Please check that the title and the abstract of the manuscript are brief, yet explicit, even to non-specialists. The length of the title should not exceed 100 characters, and the abstract should be a single paragraph not exceeding 175 words.

6. Please also note our reference format: https://www.embopress.org/page/journal/14602075/authorguide#referencesformat.

7. At EMBO Press we ask authors to provide source data for the main manuscript figures. You will receive a separate email with instructions for providing source data with your revised manuscript, including how to upload and organize the files.

8. Please remember: digital image enhancement is acceptable practice, as long as it accurately represents the original data and conforms to community standards. If a figure has been subjected to significant electronic manipulation, this must be noted in the figure legend or in the "Materials and Methods" section. The editors reserve the right to request original versions of figures and the original images that were used to assemble the figure.

9. Our journal encourages inclusion of data citations in the reference list to directly cite datasets that were obtained from public databases. Data citations in the article text are distinct from normal bibliographical citations and should directly link to the database records from which the data can be accessed. In the main text, data citations are formatted as follows: "Data ref: Smith et al, 2001" or "Data ref: NCBI Sequence Read Archive PRJNA342805, 2017". In the Reference list, data citations must be labeled with "[DATASET]". A data reference must provide the database name, accession number/identifiers, and a resolvable link to the landing page from which the data can be accessed at the end of the reference. Further instructions are available at: https://www.embopress.org/page/journal/14602075/authorguide#referencesformat.

10. We request authors to consider both actual and perceived competing interests. Please review our policy (https://www.embopress.org/page/journal/14602075/authorguide#conflictsofinterest) and update your competing interests statement if necessary. Please name this section 'Disclosure and competing interests statement' and place it after the Acknowledgements section.

11. Please note that all corresponding authors are required to provide an ORCID ID upon submission of a revised manuscript (https://orcid.org/). Please find instructions on how to link your ORCID ID to your account in our manuscript tracking system in our Author guidelines (https://www.embopress.org/page/journal/14602075/authorguide#authorshipguidelines).

12. We use CRediT to specify the contributions of each author in the journal submission system. CRediT replaces the author contribution section, which should be removed from the manuscript. Please use the free text box to provide more detailed descriptions. See also guide to authors: https://www.embopress.org/page/journal/14602075/authorguide#authorshipguidelines.

13. Further information is available in our Guide For Authors: https://www.embopress.org/page/journal/14602075/authorguide

14. We would also welcome the submission of cover suggestions or motifs to be used by our Graphics Illustrator in designing a cover.

15. Please use the link below to submit your revision:
https://emboj.msubmit.net/cgi-bin/main.plex

Referee #1:

In this MS, Sieg et al. generated knock-in mice that express cleavage-resistant mutants of Caspase-3 (CASP3(D175A)) or Caspase-7 (CASP7(D198A)). While the two caspases have redundant functions during embryogenesis, in adult mice CASP7 is required for spermatogenesis and male fertility by controlling sphingolipid homeoastasis, while CASP3 is crucial for lymphoid tissue development by regulating interferon (IFN) signaling. Casp3DA/DA;Casp7DA/DA mice died between E13.5 and weaning, and cells from these mice were completed protected from BH3-mimetic induced apoptosis.

The authors first show that cleaved CASP3 was detected in the spleen of WT and Casp7DA/DA mice, but not in Casp3DA/DA mice. Conversely cleaved CASP7 was detected in the spleen of WT and Casp3DA/DA mice, but not in Casp7DA/DA mice. They then verified that the respective CASP mutants cannot be cleaved to their active fragments by the addition of recombinant CASP8 or CASP9 anymore. Interestingly CASP3 appears to be the major executioner caspase since apoptosis induced by TNF/birnipant or the BH3 mimetics (ABT737/S-63845) was only diminished in Casp3DA/DA but not in Casp7DA/DA mice. Lack of CASP3 processing was also associated with reduced CASP8 and CASP9 processing confirming that both initiator caspases cannot be activated downstream of CASP3 but not CASP8. RIPK1 and RIPK3 were also not cleaved in BH3-mimetic- or TNF/birinapant-treated Casp3DA/DA cells, but this was insufficient to activate pMLKL-mediated necroptosis.

The most interesting aspect of this MS is the different impact of the Casp7DA/DA and Casp3DA/DA mutants in adult mice. Male Casp7DA/DA mice were infertile. Sperms of these mice did not show gross morphological alteration but they exhibited reduced motility indicating that CASP7 activation was required for the terminal differentiation of sperms. However, since no changes in apoptosis were detected in the testes of the Casp7DA/DA mice, it was suggested that CASP7 activation controls

spermatogenesis independent to apoptosis. Interestingly transcriptome analysis shows that lack of CASP7 activation in testicular tissues reduces the expression of genes involved in several processes coordinating spermatogenesis. Mechanistically, CASP7 seems to proteolytically cleave acidic sphingomyelinase, thereby activating it leading to the production of ceramides which seem to be crucial for spermatogenesis. Indeed, while Casp3DA/DA showed ASM cleavage on Western blots, this was not the case in lysates of Casp7DA/DA mice. Asm-/- mice have also shown to be sub-fertile like Casp7DA/DA mice and lipidomics analysis shows reduced long-chain ceramides in these animals.
By contrast Casp3DA/DA mice showed extensive lymphofollicular hyperplasia characterizes by enlarged lymph follicles in cervical lymph nodes without altering a distinct cellular fraction within lymphatic tissues. However, both B and T cells showed a more activated phenotype. Transcriptome analysis of total spleen revealed that IFN signaling was significantly upregulated in Casp3DA/DA mice leading to a chronic perivascular inflammation. This role of CASP3 is not new as it has been previously published that activation of CASP3 is required to dampen aberrant IFN production by inactivating the cGAS/STING of MAVS. What is new is that the anti-inflammatory function of Casp3DA/DA cannot be replaced by Casp7DA/DA.

In general, the experiments have been concisely conducted and the data presented are convincing and for most parts novel. The MS therefore deserves publication in EMBO J. However, it is not clear how much and by what mechanism CASP7-mediated cleavage of ASM contributes to spermatogenesis.

Major points:

1. The Western blots in Fig. 3F showing ASM cleavage in Casp3DA/DA but not in Casp7DA/DA mice are not convincing. The majority of ASM is not cleaved and the cleavage fragments are barely visible. This is also true for the MAVS and cGAS cleavage fragments in the spleen in Fig. 5A.
2. Although less long-chain ceramides are produced in Casp7DA/DA mice, there is no evidence presented that CASP7-mediated ASM cleavage in fact activates the enzyme. What is the cleavage site? What happens with the ASM enzyme when it is cleaved. Some structural analysis into ASM and an ASM activity assay of testicular lysates may answer these questions.
3. A simple explanation why CASP3 cannot replace CASP7 for sperm differentiation is that CASP3 is expressed at a much lower lever than CASP7 (which is indeed the case based on Fig. 3F). Alternatively, CASP3 may not be activated in these lysates. Would a treatment of the lysate with BH3-mimetics activate CASP3? And could BH3-mimetics trigger ASM cleavage via CASP3 in Casp7DA/DA lysates?
4. Do Casp-7-/- mice show the same spermatogenesis defect?

Referee #2:

In this study, Sieg et al. generated elegant genetic mouse models with disabled proteolytic maturation of either caspase-3 or caspase-7, thereby showing that these apoptotic caspases exert non-overlapping physiological functions in mice. Whereas activated caspase-7 was non-redundantly needed for spermatogenesis and male fertility, caspase-3 non-redundantly prevented splenic and lymphoid inflammatory responses by restricting IFN signaling. These findings are novel and refine our knowledge on the complex interplay of caspases in health and disease. This study will be of particular interest to the cell death field. In general, I support publication of this study in EMBO Journal, provided that the authors address the questions below.

Major comments:

1. The authors show that Casp3DA/DACasp7DA/DA mice do not survive. E13.5 embryos were present, but none of these mice were present upon weaning. However, there is a too long time window in between E13.5 and weaning for the authors to conclude in the abstract and in the discussion that these caspases are 'needed for embryonic development'. Can the authors analyze more precisely when Casp3DA/DACasp7DA/DA mice succumb? Are these mice born as reported for Casp3-/-Casp7-/- mice by Lakhani et al? Additionally, histological analyses of these embryos/pups could shed some light on the causes of lethality. Do they show defects in cardiac development like Casp3-/-Casp7-/- mice do?

2. In Fig 2E-F, the authors show that caspase-7 cleavage is not involved in caspase-1 mediated pyroptosis based on LPS/Nigericin induced cell death and caspase-1 cleavage. These data would be stronger with additional quantitative analyses of mature IL-1b secretion. In addition, given the fact that caspase-7 deficient mice are resistant to LPS-induced endotoxic shock (https://doi.org/10.1182/blood-2008-09-178038), it might be interesting to evaluate the response of Casp7DA/DA BMDMs to non-canonical LPS-induced inflammasome responses, as well as the sensitivity of Casp7DA/DA mice to LPS-induced shock in vivo.

Minor comments:

1. In several figures the authors show the results of technical replicates, precluding statistical analyses. In most cases this is fine, but I feel that Fig 1E would benefit from biological replicates and statistics to support the combined involvement of casp3 and casp7 in intrinsic but not extrinsic apoptosis.

2. Fig 2C: use Ripk3 instead of RIP3.

3. In Fig 3B it seems as if the eosin staining of the Casp3DA sections did not work in comparison to the other genotypes. Can you provide better stainings?

4. In Fig 5E it is odd that the F4/80 staining does not show positive cells, as the lungs should have interstitial and alveolar macrophages. Did the staining really work?

5. Fig EV3: In the B panel what is M/ml? In C the pictures do not show anything, please provide better quality pictures. And in D, ideally please provide a positive control for clCasp3 since none of the pictures show positive cells.

6. Fig EV4B: it seems that the Casp3DA/DA cervical lymph node is full of neutrophils. This is not mentioned or discussed in the results. The same figure also shows a Bcl6 staining, but this is not clear. Please provide a better staining.

7. Based on Fig 4D, the authors claim 'increased fractions of CD95+ and MHC class II+ B cells and CD69+ and CD44+ memory T cells'. However, not all of these statements are supported by statistically significant differences.

8. Also in figure 4D it is a odd to only look at proportions of the immune cells and not absolute counts, especially considering the lymphoproliferative phenotype. With only proportion data it is hard to conclude that there are more activated cells when it could just be that the excessive proliferation is altering the proportions.

9. In Fig 4E, please provide separate data for CD4 and CD8 T cells to show whether the higher CD4/8 ratio is caused by increased CD4 or decreased CD8 T cell counts.

10. Fig EV4C: macrophages, not makrophages.

11. Since the claimed upregulations in Casp3DA/DA cells of RIG-I, STAT1 and IRF3 in Fig 5C are not super convincing, and since they are not accompanied by data showing their Casp3-dependent cleavage, I would argue to omit these data and to solely base the IFN signature on the more convincing cGAS, MAVS and P-STING data in these figures.

Referee #3:

Summary

Sieg and colleagues describe knock-in mice expressing cleavage-resistant CASP3 (D175A) and CASP7 (D198A), dubbed CASP3^DA/DA and CASP7^DA/DA. They confirm CASP3 as the dominant executioner in apoptosis and show that double mutants die between E13.5 and weaning, mirroring prior CASP3/7 DKO reports. Adult phenotypes include CASP7-linked spermatogenesis defects and CASP3-driven lymphoid hyperplasia. While the study provides a useful genetic resource and highlights some non-redundant roles, it is largely confirmatory. The absence of substrate identification or genetic rescue limits mechanistic insight, making this more of a descriptive dataset than a conceptual advance. Minor points follow.

Note to authors: The resolution of all images (western, H&E, etc.) have been downsampled in the PDF. This makes it difficult to interpret any histological images and appreciate more subtle cleavage patterns, such as the ASM blot in Fig. 3F. In particular, Fig. EV3C displays as a grey box. Please remove compression on images.

Minor points
• Authors should indicate where cleavage-specific antibodies to CASP3 (Asp175) and CASP7 (Asp198) were used. This would be helpful in demonstrating the specificity of the Casp3DA/DA/Casp7DA/DA point mutations (i.e. Fig. 1B).
• In EV1B, EV2B, and EV2C: Are different blot exposures being spliced together and presented on a single panel? On close inspection, the dashed lines seem to separate blots of different exposures. If so, this is misleading. Please use the same exposure for each antibody combination. As currently displayed, there seems to be an increase in CASP7 total protein in the left three lanes (recombinant CASP9 treated) versus unstimulated in EV1B.
• The authors do not formally show lack of cell death in Casp3DA/DA/ Casp7DA/DA embryos. A TUNEL stain in conjunction with cleaved CASP3 staining in WT vs Casp3DA/DA/Casp7DA/DA embryos would strengthen the claim that "CASP3 and CASP7 provide redundant functions during embryonic development, likely be executing apoptosis."
• In Fig. 3F, the unspecific band detected by CASP3 falls at roughly the same molecular weight as cleaved CASP3. It may be more informative to use the cleavage specific CASP3 antibody here to demonstrate that cleaved CASP3 is not being up-regulated in CASP7DA/DA animals
• A TUNEL stain in CASP7DA/DA testes sections would bolster the interpretation that the reduction in sperm is not due to apoptosis
• Fig. 3C is missing a scalebar
• In Fig. 4D and E, the authors note increased fractions of CD95+ B cells, MHCII high B cells, and CD69+ T cells. They also

note increased CD4 to CD8 Y cell ratios in spleen and cervical lymph node. However, none of these measurements seemingly reach statistical significance, based on the figure panel itself. The authors should clarify this interpretation.
• Fig. EV4D has a floating "h" on the y-axis in Pro/Pre cells panel
• Please indicate where drugs (Birinapant, TNF, s-63845, ABT-737, LPS, Nigericin, etc) were purchased from

Referee #4:

This is an interesting study that identifies distinct requirements for caspase-3 and caspase-7 in vivo for the suppression of Type I IFN signaling in the lymphoid compartment and spermatogenesis, respectively. Processing-defective Casp7DA mice exhibited spermatogenesis defects, whereas Casp3DA mice exhibited splenomegaly and perturbation of the ratios of CD4/CD8 T-cells and other lymphoid abnormalities, potentially related to imbalanced Type I IFN signaling.

1. In Figure 1E and 1F it is shown that BH3 mimetic-induced cell death is greatly suppressed in the Casp-3DA/Casp-7DA MEFs, at least at the endpoint of 8h that is shown. However, given that BAX/BAK channel opening is typically considered the point in commitment in cell death, with caspase-3/7 activation only necessary to produce the apoptotic phenotype, can the authors clarify whether the Casp-3DA/Casp-7DA cells survived BH3 mimetic treatment for several days, or whether these cells simply died with a kinetic delay. Furthermore, I feel that it's important to clarify what the phenotype of cell death was when Casp-3DA/Casp-7DA MEFs did die, presumably this was very different to the WT phenotype. Some high resolution confocal images would be useful here.
2. In Figure 2, there is no positive control for the pMLKL antibody used. This should be provided as a supplementary figure.
3. In Figure 2F GSDMD and possibly also GSDME proteolysis should be examined, as these are the key caspase substrate driving pyroptosis.
4. The ASM cleavage products depicted in Figure 3F (testicular lysates) is not convincing and could be improved. Perhaps the authors could probe the MEF lysates used in Figure 1 to determine ASM cleavage status in response TNF/Birnapant as well as ABT/S6 treatment in the various genotypes.
5. How caspase-7 may become activated during spermatogenesis is not discussed. Is it that caspase-3 cannot cleave ASM or that it is not activated during spermatogenesis? Can enough sperm be obtained from the various genotypes to promote apoptosis using BH3-mimetics to determine whether (a) caspase-3 and caspase-7 become proteolytically processed in sperm in response to a pro-apoptotic stimulus and (b) is ASM proteolytically processed in WT versus Casp-3DA or Casp-7DA sperm under these conditions?
6. Caspase activity has been implicated in T-cell activation in previous studies (e.g. Chun HJ et al., 2002; Salmena et al. 2003; Misra et al. 2008). As an alternative explanation for the lymphocyte hyperplasia observed in Casp-3DA animals, the authors should isolate T- and B-lymphocytes from the various genotypes and examine TCR- as well as BCR-induced proliferation, IL-2/IL-4 production as well as other markers of T- and B-cell activation. Caspase processing activation should also be monitored in response to TCR and BCR stimulation. It seems possible that the previously documented role for caspase-8 activity downstream of TCR activation may promote activation of caspase-3 in T-cells to promote a non-apoptotic endpoint. Do MAVS, cGAS and other components of this pathway undergo caspase-3-dependent proteolysis in response to TCR or BCR engagement in vitro?

Minor comments

1." In vertebrates, the apoptotic caspases can be further subdivided into initiator caspases
(caspases-8, -9 and -10) and executioner caspases (caspases-3, -6 and -7)."
Comment: I would suggest re-wording all references to 'apoptotic caspases' to 'pro-apoptotic caspases' or 'apoptosis-associated caspases'. 'Apoptotic' refers to a mode of cell death and a caspase cannot be 'apoptotic', rather it can promote apoptosis (i.e. pro-apoptotic caspase) or inflammation (pro-inflammatory caspase).

2. "Although apical caspases, including the initiator CASP8 and CASP9 and the inflammatory CASP1, respond to very different signals and are activated in distinct
activation platforms, they all share the capability to cleave the linker peptide of executioner CASP3 (at D175) and CASP7 (at D198), which is viewed as the point-of no- return in apoptosis (Fuentes-Prior & Salvesen, 2004)."
Comment: The point of no return in the intrinsic pathway to apoptosis is generally viewed as BAX/BAK-dependent release of cytochrome c, rather than activation of caspase-3 and/or caspase-7 (or indeed caspase-9). If the latter caspases are eliminated, cells typically die via necrosis resulting from release of mitochondrial intermembrane space constituents and ATP depletion. The authors should clarify this section to avoid confusion.

3. "Similar to extrinsic apoptosis, PARP cleavage induced by the BH3 mimetics ABT737/S-63845 was only reduced in Casp3DA/DA cells (Fig 2B). Failure of CASP3 activation was also associated with reduced CASP9 processing and lack of CASP8 processing in response to ABT737/S-63845."
Comment: This indicates a feedback loop for activation of caspase-9 via caspase-3. This has been demonstrated previously and should be cited here.

4. "Similar to initiator CASP8 and CASP9, CASP1 has also been shown to process CASP3 and CASP7 during the course of

macrophage-mediated anti-bacterial immunity (de Vasconcelos et al, 2020; Lamkanfi et al., 2008). In order to examine the role of executioner caspase processing in pyroptosis, we isolated bone marrowderived macrophages (BMDMs) from mice and exposed them to LPS/nigericin which activates the NLRP3 inflammasome subsequently inducing pyroptosis (Fritsch et al., 2019; Simpson et al, 2022). These results indicated that the proteolytic activation of executioner caspases is unlikely decisive for the execution of pyroptosis (Figs 2E and F)."
Comment: The authors should describe the observations here more explicitly.

5. Discussion: "CASP3 and CASP7 represent the two major executioner caspases that upon activation induce apoptotic cell death. They are closely related and are considered to have a virtually indistinguishable substrate specificity during apoptosis (Stennicke et al, 2000)"
Comment: This statement is not quite correct. Studies have shown that caspases-3 and -7 do exhibit distinct activity towards a range of specific substrates and that caspase-3 does much of the global proteolysis during apoptosis (Walsh et al., 2008 PNAS).

6. "CASP9 deficiency in mice leads to perinatal lethality which is associated with defects in neuronal tube closure, presented already at E10.5 and progresses to an expansion of the midbrain region underneath the fetal skin tissue at E13.5 (Kuida et al, 1998). None of these alterations could be observed in Casp3DA/DA/Casp7DA/DA embryos at E13.5 (Fig 1G), yet they died between E13.5 and before weaning (Figs EV1E and F). These observations are in line with a previous report analyzing Casp3-/-/Casp7-/- double-knockout mice (DKO) (Lakhani et al, 2006). Casp3-/-/Casp7-/- mice were present at normal Mendelian numbers at E20, but died rapidly after birth suggesting that unlike CASP9, neither CASP3 nor CASP7 is important for neuronal development. Independently, our data indicated that CASP3 and CASP7 provide redundant functions during embryonic development, likely by executing apoptosis."
Comment: The authors should discuss the implications of the differences observed between CASP9 deficiency in embryos and CASP3/CASP7 DKO embryos as well as Casp3DA/DA/Casp7DA/DA embryos. What are the implications of these differences? That caspase-9 cleaves additional substrates other than caspase-3 and caspase-7?

7.The authors repeatedly use the term 'sperms'. I think that 'sperm' is the plural.

POINT-BY-POINT

Referee #1:

In this MS, Sieg et al. generated knock-in mice that express cleavage-resistant mutants of Caspase-3 (CASP3(D175A)) or Caspase-7 (CASP7(D198A)). While the two caspases have redundant functions during embryogenesis, in adult mice CASP7 is required for spermatogenesis and male fertility by controlling sphingolipid homeoastasis, while CASP3 is crucial for lymphoid tissue development by regulating interferon (IFN) signaling. Casp3DA/DA;Casp7DA/DA mice died between E13.5 and weaning, and cells from these mice were completed protected from BH3-mimetic induced apoptosis.

The authors first show that cleaved CASP3 was detected in the spleen of WT and Casp7DA/DA mice, but not in Casp3DA/DA mice. Conversely cleaved CASP7 was detected in the spleen of WT and Casp3DA/DA mice, but not in Casp7DA/DA mice. They then verified that the respective CASP mutants cannot be cleaved to their active fragments by the addition of recombinant CASP8 or CASP9 anymore. Interestingly CASP3 appears to be the major executioner caspase since apoptosis induced by TNF/birnipant or the BH3 mimetics (ABT737/S-63845) was only diminished in Casp3DA/DA but not in Casp7DA/DA mice. Lack of CASP3 processing was also associated with reduced CASP8 and CASP9 processing confirming that both initiator caspases cannot be activated downstream of CASP3 but not CASP8. RIPK1 and RIPK3 were also not cleaved in BH3-mimetic- or TNF/birinapant-treated Casp3DA/DA cells, but this was insufficient to activate pMLKL-mediated necroptosis.

The most interesting aspect of this MS is the different impact of the Casp7DA/DA and Casp3DA/DA mutants in adult mice. Male Casp7DA/DA mice were infertile. Sperms of these mice did not show gross morphological alteration but they exhibited reduced motility indicating that CASP7 activation was required for the terminal differentiation of sperms. However, since no changes in apoptosis were detected in the testes of the Casp7DA/DA mice, it was suggested that CASP7 activation controls spermatogenesis independent to apoptosis. Interestingly transcriptome analysis shows that lack of CASP7 activation in testicular tissues reduces the expression of genes involved in several processes coordinating spermatogenesis. Mechanistically, CASP7 seems to proteolytically cleave acidic sphingomyelinase, thereby activating it leading to the production of ceramides which seem to be crucial for spermatogenesis. Indeed, while Casp3DA/DA showed ASM cleavage on Western blots, this was not the case in lysates of Casp7DA/DA mice. Asm-/- mice have also shown to be sub-fertile like Casp7DA/DA mice and lipidomics analysis shows reduced long-chain ceramides in these animals.

By contrast Casp3DA/DA mice showed extensive lymphofollicular hyperplasia characterizes by enlarged lymph follicles in cervical lymph nodes without altering a distinct cellular fraction within lymphatic tissues. However, both B and T cells showed a more activated phenotype. Transcriptome analysis of total spleen revealed that IFN signaling was significantly upregulated in Casp3DA/DA mice leading to a chronic perivascular inflammation. This role of CASP3 is not new as it has been previously published that activation of CASP3 is required to dampen aberrant IFN production by inactivating the cGAS/STING of MAVS. What is new is that the anti-inflammatory function of Casp3DA/DA cannot be replaced by Casp7DA/DA.

In general, the experiments have been **concisely conducted and the data presented are convincing and for most parts novel**. The MS therefore deserves publication in EMBO J. However, it is not clear how much and by what mechanism CASP7-mediated cleavage of ASM contributes to spermatogenesis.

We thank this reviewer for his/her thoughtful critiques and very helpful recommendations. We do agree that our data about CASP7-mediated ASM cleavage and its role in spermatogenesis is rather incomplete.

Based on a recent report, demonstrating that CASP7 cleaves ASM in intestinal tissues (Nozaki et al 2022 Nature) and the original report which discovered the cleavage of the 72-kDa pro-ASM zymogen at the non-canonical cleavage site after aspartate 253 (yielding an active 57 kDa ASM) (Edelmann et al 2011 EMBO J), we initially stated (initial submission) that "*these*

data suggested that the failure of CASP7(D198A) to activate ASM represents ***one of the underlying molecular mechanisms*** *which are controlled by CASP7 and ensures male fertility*". Indeed, additional data, in particular, analysis of testicular ceramide content conveyed further support for this notion, yet didn't provide an experimental proof for such process. We believe that key experimental proof for such molecular process would be to generate mice expressing cleavage resistant ASM, which is out of the scope of the current study.

During the course of revision, we realised that further biochemical characterisation of such process is rather struggling, as the reagents needed to specifically examine ASM expression/processing are limited. In particular, anti-ASM antibodies produced multiple unspecific bands in Western blotting (WB) when we used ASM-deficient mouse embryonic fibroblasts (MEFs) as negative control (please see below). We carried out a number of experimental works to address the major points #1 and #2 of this reviewer. The majority of results obtained were, however, not very convincing and difficult to interpret (please see below).

In contrast, the new results obtained in response to the **major point 3** of this reviewer provided decisive knowledge about the role of CASP7 in spermatogenesis (please see below). As pointed by this reviewer, CASP3 was relatively low in testis. Our new investigations clearly demonstrate that in contrast to the majority of other tissues, testis express CASP7, but barely CASP3. We would like to thank this reviewer for his/her discerning view and asking us to perform these new analyses.

Major points:

1. The Western blots in Fig. 3F showing ASM cleavage in Casp3DA/DA but not in Casp7DA/DA mice are not convincing. The majority of ASM is not cleaved and the cleavage fragments are barely visible. This is also true for the MAVS and cGAS cleavage fragments in the spleen in Fig. 5A (5C or 5D?).

Unfortunately, we were not able to produce better staining for cleaved ASM in mouse testicular tissue lysates, likely due to the lack of a better specific anti-ASM antibody and/or the low amount of the cleavage product accumulating in the healthy testes. Notably, previous studies demonstrating CASP7-mediated ASM cleavage were performed after acute caspase activation by FlaTox or TNF exposure (Nozaki et al 2022 Nature; Edelmann et al 2011 EMBO J) of cultured cells or intestinal organoids, which then led to an efficient cleavage of ASM. In contrast to these experimental conditions, mouse testis does not comprise high-level caspase activity under normal/physiologic condition (see **Fig. EV4D**) and , therefore, exhibit most likely less ASM cleavage. We tried several experimental approaches to monitor ASM cleavage in cultured cells after induction of caspase activity. The results obtained were, however, difficult to interpret as anti-ASM antibody(ies) detected several bands in cultured cells, also in ASM-deficient cells (see below). Based on these observations, we decided to rephrase the respective paragraph in our revised manuscript and particularly placed less emphasis on the involvement of CASP7-medaited ASM cleavage in male fertility, as our data by its own do not convey a proof for such conclusion. This notion was basically drawn upon previous studies demonstrating CASP7-medaited ASM cleavage and independent observations frequently indicating that sphingolipid metabolism controls male fertility.

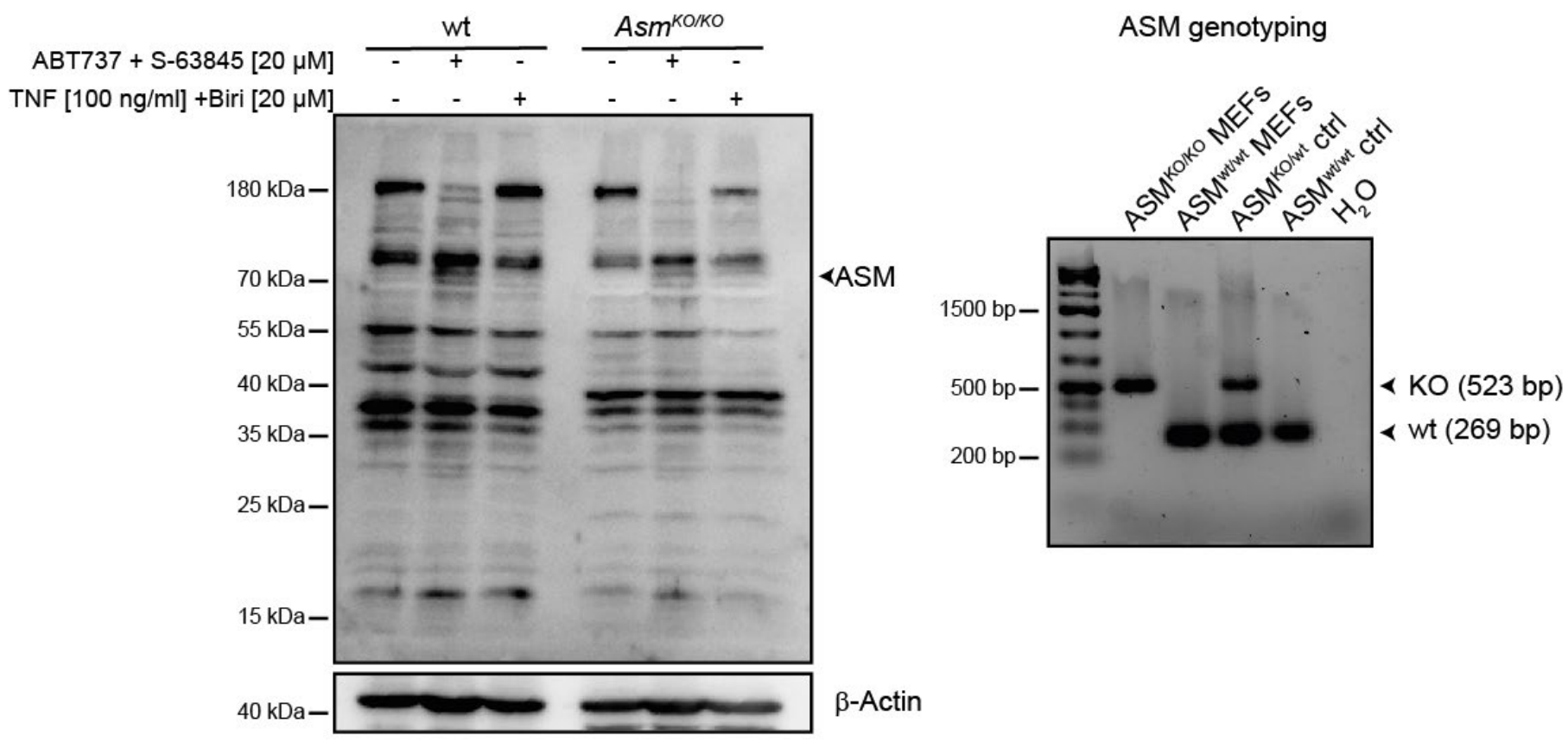

We are not sure, whether this reviewer addresses **Fig. 5C** or **Fig. 5D,** as **Fig. 5A** does not summarise WB analysis. We were not able to provide a stronger staining of cleaved cGAS and MAVS in crude spleen lysates, even after increasing the protein amount loaded for WB or overexposure of WB membranes for better detection of cleaved MAVS/cGAS fragments. Notably, treatment of cultured cells with BH3 mimetics, which induced ample CASP3/7 activation in almost all cells (**Fig. 6D**), reveal rather weak detection of cleaved MAVS/cGAS fragments, specifically only in cells expressing active CASP3. This is due to either relatively low MAVS/cGAS processing by CASP3 or simply because the cleaved products are less detectable (lacking the immunogenic epitope) by the available antibodies. As the minority of splenocytes activate CASP3/7 under normal condition (new **Fig. EV1B**), the available antibodies will unlikely be able to detect relatively low-level cleaved MAVS/cGAS fragments in the crude splenic lysate.

Independently, we realised that the insufficient quality of the cleaved ASM and also cleaved cGAS/MAVS is at least partly due to the fact that the resolution of our submitted figures has been somewhat downsampled, likely upon PDF conversion (also pointed by the **Referee#3**). We will make sure that the revised figures will be presented in a best quality, hopefully, immediately answering this point of the reviewer.

2. Although less long-chain ceramides are produced in Casp7DA/DA mice, there is no evidence presented that CASP7-mediated ASM cleavage in fact activates the enzyme. What is the cleavage site? What happens with the ASM enzyme when it is cleaved. Some structural analysis into ASM and an ASM activity assay of testicular lysates may answer these questions.

The original study, which discovered the cleavage of ASM by CASP7, already showed that CASP7 directly binds the 72-kDa pro-ASM zymogen in endocytic vesicles in response to TNF. Pro-ASM is then cleaved in TNF-R1 receptosome by CASP7 at the non-canonical cleavage site after aspartate 253 which leads to accumulation of the active 57 kDa ASM (Edelmann et al 2011 EMBO J). This information is now incorporated in the revised manuscript.

We could not detect altered ASM activity in testicular lysates (see below and **Fig. EV4H**). In cultured MEFs, basal ASM activity was reduced in *Casp3*$^{DA/DA}$ and, in particular, in *Casp7*$^{DA/DA}$ MEFs, which could serve as a proof for our conclusion and confirmation of previous studies describing the activation of ASM by CASP7. We would like to specify that our study describes the alteration of spermatogenesis in *Casp7*$^{DA/DA}$ mice, which has also been reported in mice or human lacking ASM or suffering malfunctional sphingolipid metabolism. Based on findings derived from more recent studies, indicating the activation of ASM by CASP7, we basically suggested that "*the failure of CASP7(D198A) to activate ASM represents* ***one of the underlying molecular mechanisms*** *which are controlled by CASP7 and ensures male fertility*" (from the original submission) and provided some confirmatory limited evidence (ceramide content in testes).

Based on some additional new evidence, which were collected during the phase of revision (new **Fig. 2** and new **Fig. 4G**, see response to point #3 of this reviewer), we decided to rephrase the respective paragraph in our revised manuscript and particularly placed less emphasis on the involvement of CASP7-medaited ASM cleavage in male fertility as our data by its own do not convey a proof for such conclusion. Noteworthy, ASM is ubiquitously expressed across various tissues and its activity is induced by a plethora of diverse tissue specific triggers. The regulation of ASM *via* CASP7 in testes represents a topic for future investigations and is beyond the scope of our current study.

New Fig. EV4H:

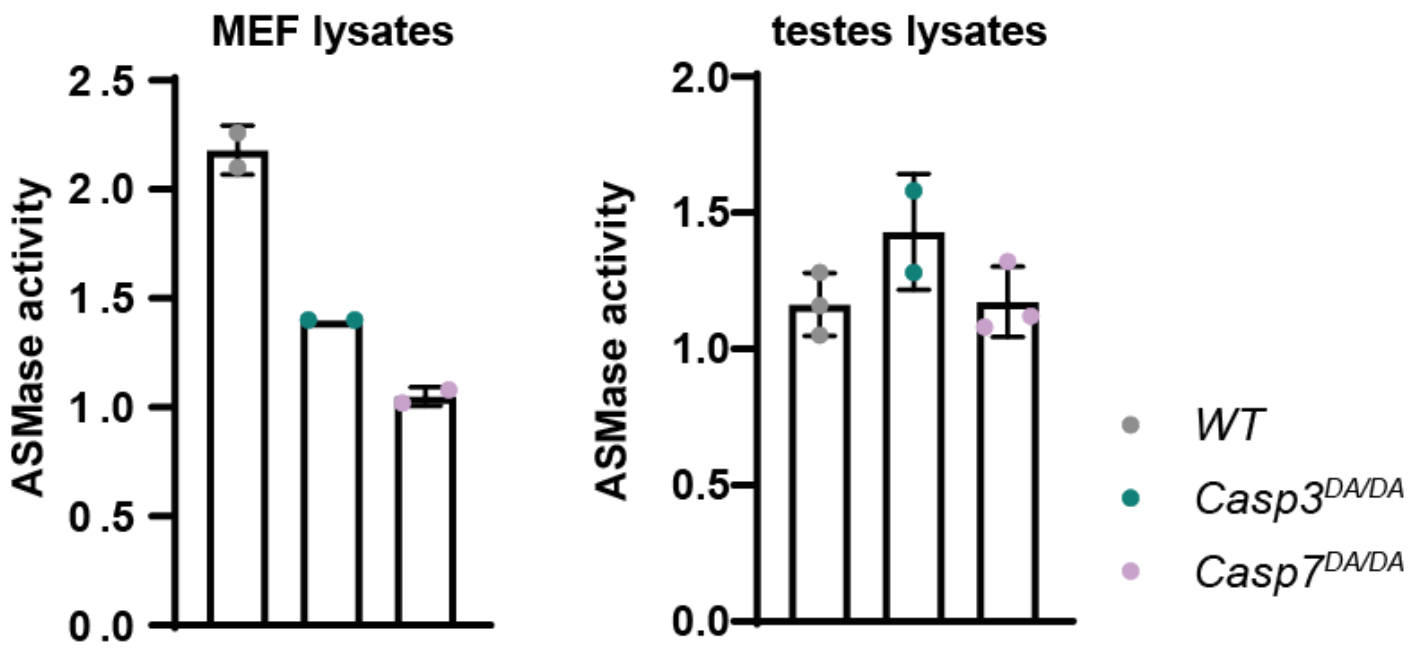

3. A simple explanation why CASP3 cannot replace CASP7 for sperm differentiation is that CASP3 is expressed at a much lower lever than CASP7 (which is indeed the case based on Fig. 3F). Alternatively, CASP3 may not be activated in these lysates. Would a treatment of the lysate with BH3-mimetics activate CASP3? And could BH3-mimetics trigger ASM cleavage via CASP3 in Casp7DA/DA lysates?

We thank this reviewer for raising this very decisive point. This indeed turned to be a simple explanation for this topic (please see below).

In order to examine whether specific involvement of CASP7 in spermatogenesis is based on its specific expression in testes, we analysed CASP3 and CASP7 expression levels in different mouse tissues (3 mice) (new **Fig. 4G**). Whereas both CASP3 and CASP7 were strongly expressed in tissues such as spleen and colon, CASP3 was barely detectable in testes. In contrast, CASP7 was strongly expressed in testes, but was barely detectable in brain or kidney. These data are completely in line with the data derived from tissue-specific atlas of mouse protein expression (Huttlin et al 2010) and indicate that the dependency of testicular tissue on CASP7 is due to the lack of CASP3.

Thus, our data indicate that testicular tissues, which barely express CASP3, respond to upstream pro-apoptotic cascades just by activating CASP7, which in turn induces membrane blebbing leading to cellular shrinkage (new **Fig. 2**) required for successful spermatid maturation/individualisation. This notion is completely in line with the data obtained in BAX-deficient mice (Knudson et al 1995), which also developed male infertility. Collectively, these observations indicate that the mitochondrial apoptotic pathway in testes leads to activation of CASP7, which is required for successful spermatogenesis.

New Fig. 4G:

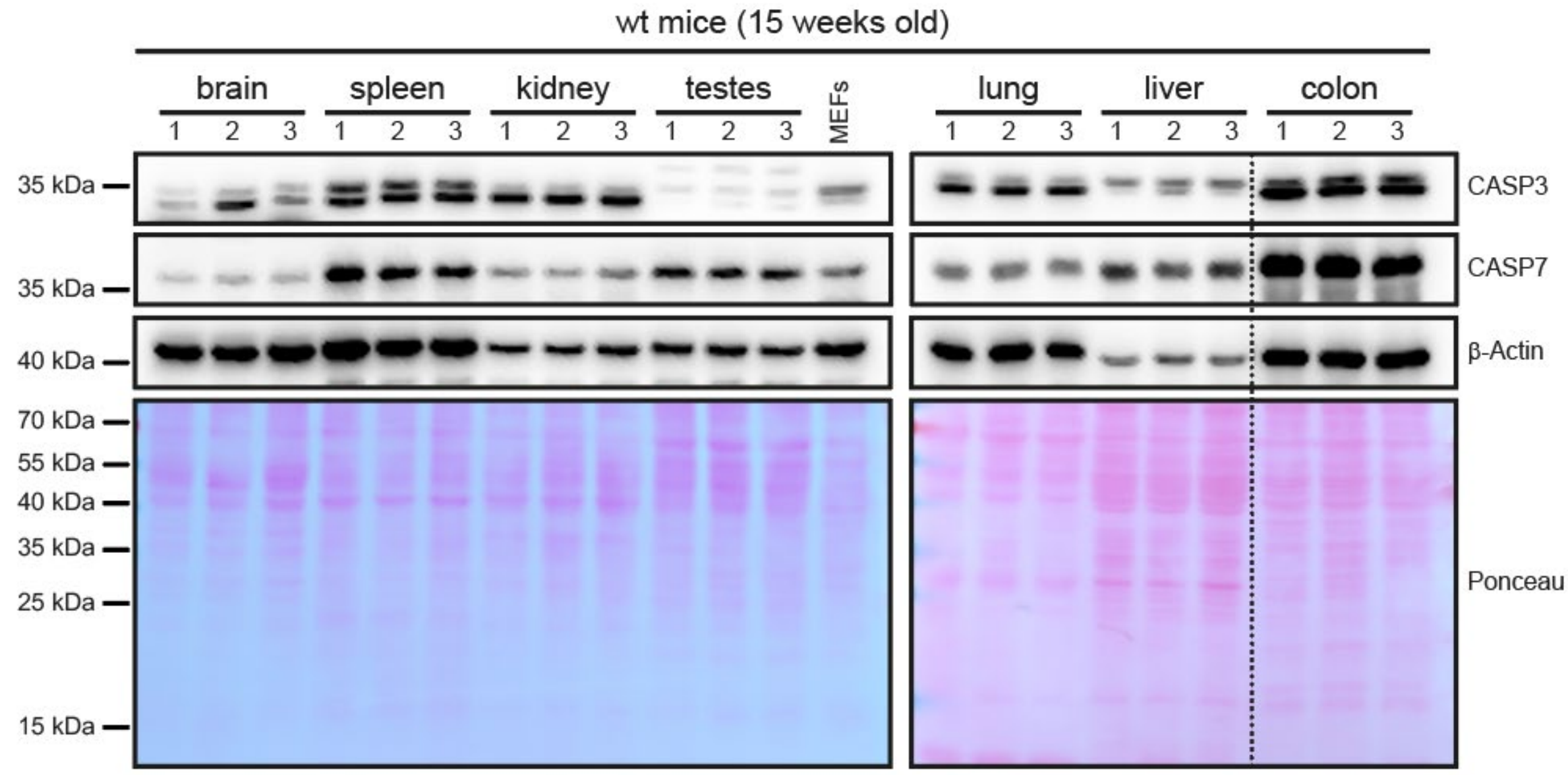

4. Do Casp-7-/- mice show the same spermatogenesis defect?

A defect in spermatogenesis has not been described in *Casp7*$^{-/-}$ mice. The data provided by using *Casp7*$^{-/-}$ mice are, however, difficult to interpret, as these mice were primarily reported to die during embryonic development (Zheng et al 1999). Later studies then indicated that *Casp7*$^{-/-}$ 129/SvJ mice survived weaning without developing any phenotype (Lakhani et al 2006). *Casp7*$^{-/-}$ mice were later backcrossed onto the C57BL/6 background (6 generations) in order to perform comparable physiological analysis between the two executioner caspases (Lakhani et al 2013). These investigations of Caspase-3/7 gene knockout models failed to provide robust information, in particular, in a comparable manner due to the strain-specific genetic background effects of mice deficient in either caspase. Whether or not *Casp7*$^{-/-}$ and *Casp7*$^{DA/DA}$ mice develop and behave similarly, remain an open question for future investigation with specific consideration of strain-specific genetic backgrounds (please also see the second paragraph of the Discussion).

Referee #2:

In this study, Sieg et al. generated elegant genetic mouse models with disabled proteolytic maturation of either caspase-3 or caspase-7, thereby showing that these apoptotic caspases exert non-overlapping physiological functions in mice. Whereas activated caspase-7 was non-redundantly needed for spermatogenesis and male fertility, caspase-3 non-redundantly prevented splenic and lymphoid inflammatory responses by restricting IFN signaling. These findings are novel and refine our knowledge on the complex interplay of caspases in health and disease. This study will be of particular interest to the cell death field. In general, I support publication of this study in EMBO Journal, provided that the authors address the questions below.

We thank this reviewer for his/her evaluation and highlighting the specific need to extensively analyse the interplay of Caspases for physiologic and pathophysiologic processes. We especially thank for the very helpful advices to further strengthening of our findings presented.

Major comments:

1. The authors show that Casp3DA/DACasp7DA/DA mice do not survive. E13.5 embryos were present, but none of these mice were present upon weaning. However, there is a too long time window in between E13.5 and weaning for the authors to conclude in the abstract and in the discussion that these caspases are 'needed for embryonic development'. Can the authors analyze more precisely when Casp3DA/DACasp7DA/DA mice succumb? Are these mice born as reported for Casp3-/-Casp7-/- mice by Lakhani et al? Additionally, histological analyses of these embryos/pups could shed some light on the causes of lethality. Do they show defects in cardiac development like Casp3-/-Casp7-/- mice do?

In the revised manuscript, we included additional data to better monitor the death of *Casp3*$^{DA/DA}$/*Casp7*$^{DA/DA}$ mice. *Casp3*$^{DA/DA}$/*Casp7*$^{DA/DA}$ embryos were present at the expected Mendelian frequencies at embryonic day 13.5 (E13.5) but died before or shortly after birth, as we didn't observe any mice at early postnatal P0 (see tables below, new **Fig. EV1E**). One *Casp3*$^{DA/DA}$/*Casp7*$^{DA/DA}$ embryo at E18.5 was observed at about expected Mendelian frequency (1.4 was expected) and appeared normal without any obvious alteration (see below, new **Fig. 1F**). This suggests that neither activation of CASP3 nor Casp7 are important for brain development, which is in line with the data obtained in *Casp3*$^{-/-}$/*Casp7*$^{-/-}$ mice on the C57BL/6 genetic background (Lakhani et al, 2013). We were, however, not able to collect statistically relevant numbers of *Casp3*$^{DA/DA}$/*Casp7*$^{DA/DA}$ embryos for further in-depth histological investigations such as cardiac development. Noteworthy, for the analysis of E18.5 embryos and P0 progenies, we required a specific permission by the local government (3 months duration) and intensive breading of *Casp3*$^{wt/DA}$/*Casp7*$^{wt/DA}$ embryos to yield the required number of *Casp3*$^{DA/DA}$/*Casp7*$^{DA/DA}$ embryos (1/16 chance, 6.25%). Even after the extension of revision time for additional 3 months to specifically address this issue, we did not obtain the required numbers. We hope that this reviewer agrees that such investigations could be rather a matter of future extensive investigations, in particular, by comparing the phenotypic alteration of *Casp3*$^{DA/DA}$/*Casp7*$^{DA/DA}$ mice with CASP8- or CASP9-deficinet mice.

New Fig. EV1E:

Casp3								
$Casp3^{DA/wt}$ $Casp7^{DA/wt}$ x $Casp3^{DA/wt}$ $Casp7^{DA/wt}$								
	**E13.5**		**E18.5**		**P0**		**weaned**	
**Genotype**	**expected**	**observed**	**expected**	**observed**	**expected**	**observed**	**expected**	**observed**
$Casp3^{wt/wt}$ $Casp7^{wt/wt}$	3.625	4	1.4375	0	2.75	5	5.0625	7
$Casp3^{wt/wt}$ $Casp7^{DA/wt}$	7.25	7	2.875	3	5.5	13	10.125	12
$Casp3^{wt/wt}$ $Casp7^{DA/DA}$	3.625	5	1.4375	2	2.75	4	5.0625	4
$Casp3^{DA/wt}$ $Casp7^{wt/wt}$	7.25	7	2.875	3	5.5	4	10.125	7
$Casp3^{DA/wt}$ $Casp7^{DA/wt}$	14.5	11	5.75	7	11	6	20.25	19
$Casp3^{DA/wt}$ $Casp7^{DA/DA}$	7.25	7	2.875	3	5.5	5	10.125	12
$Casp3^{DA/DA}$ $Casp7^{wt/wt}$	3.625	6	1.4375	1	2.75	5	5.0625	13
$Casp3^{DA/DA}$ $Casp7^{DA/wt}$	7.25	7	2.875	3	5.5	2	10.125	7
$Casp3^{DA/DA}$ $Casp7^{DA/DA}$	3.625	4	1.4375	1	2.75	0	5.0625	0
**Total**	**58**	**58**	**23**	**23**	**44**	**44**	**81**	**81**

New Fig. 1F:

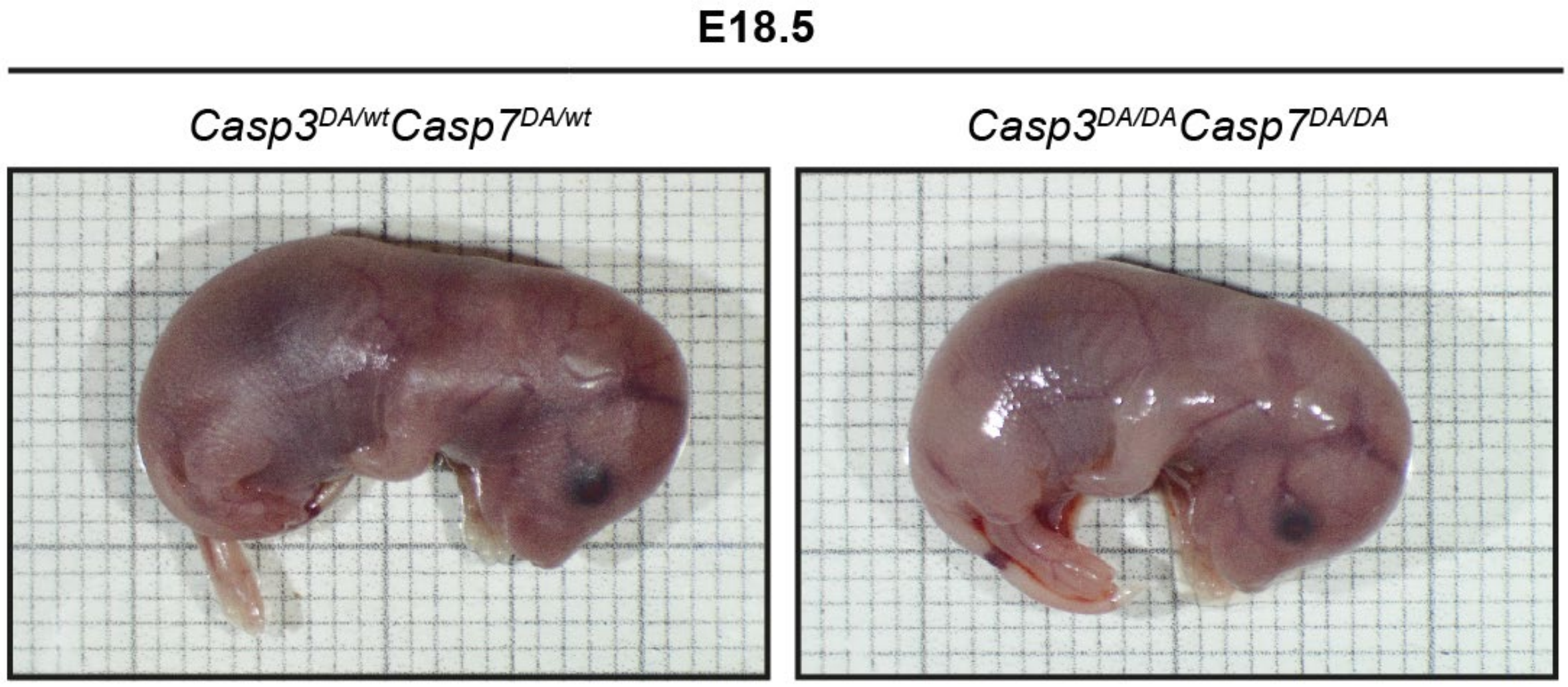

2. In Fig 2E-F, the authors show that caspase-7 cleavage is not involved in caspase-1 mediated pyroptosis based on LPS/Nigericin induced cell death and caspase-1 cleavage. These data would be stronger with additional quantitative analyses of mature IL-1b secretion. In addition, given the fact that caspase-7 deficient mice are resistant to LPS-induced endotoxic shock (https://doi.org/10.1182/blood-2008-09-178038), it might be interesting to evaluate the response of Casp7DA/DA BMDMs to non-canonical LPS-induced inflammasome responses, as well as the sensitivity of Casp7DA/DA mice to LPS-induced shock in vivo.

As requested, quantitative analysis of mature IL1β and also GSDMD cleavage are included in the revised manuscript (new **Fig. 3E-F,** see below). We also included some analyses by using BMDMs isolated from *$GsdmD^{D276A/D276A}$* mice (non-cleavable Gasdermin D) (*$GsdmD^{DA/DA}$*) (new **Fig. EV3G-I**) showing that cell death (LDH release) and IL1β secretion from BMDMs in response to LPS/Nigericin treatment is mainly derived from GSDMD cleavage. These data, however, cannot exclude the possible involvement of CASP3/7 in pyroptosis under different experimentla setting. Theferore, we specified in the revised manuscrpt that "under the experimetal setup used in our anaysis, CASP3/7 activation in BMDMs is not decisive for cell death and IL1β secretion".

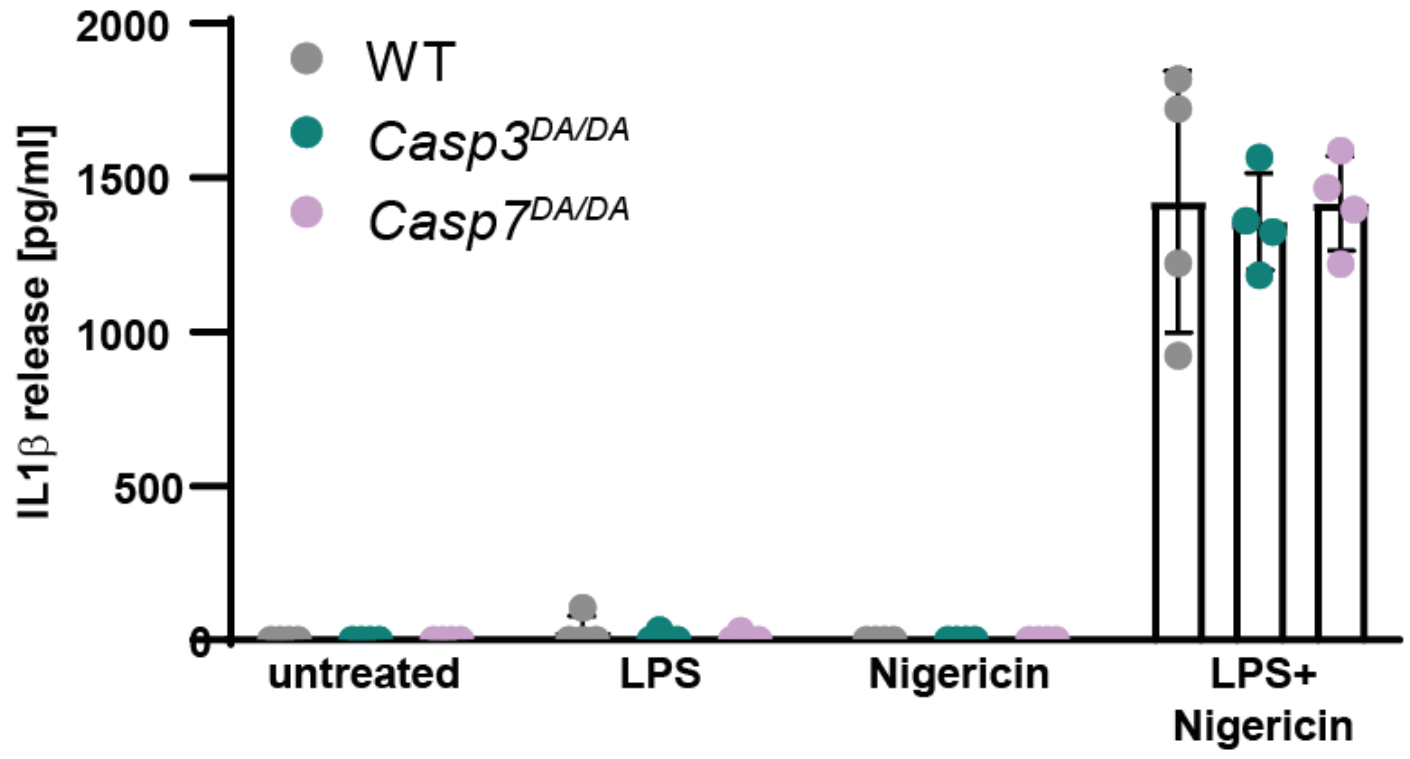

To examine the role of CASP3/7 in response to intracellular LPS, we employed Lipofectamin transfection assay and used LPS-B4 and LPS-K12 varinats or infected BMDMs with *Shigella flexneri* as previously described (Kayagaki et al Nature 2015) (see below). In all conditions, we could detect some protective effects in BMDMs expressing GSDMD(DA), lacking pyroptosis. BMDMs expressing CASP3(DA) or CASP7(DA) respond similar to WT BMDMs. However, we decided not to include these data into the revised manuscript as BMDMs were highly susceptible to Lipofectamin alone and such analysis, in our view, should be best performed in cells lacking CASP11 as control in combination with CASP7(DA) or CASP3(DA).

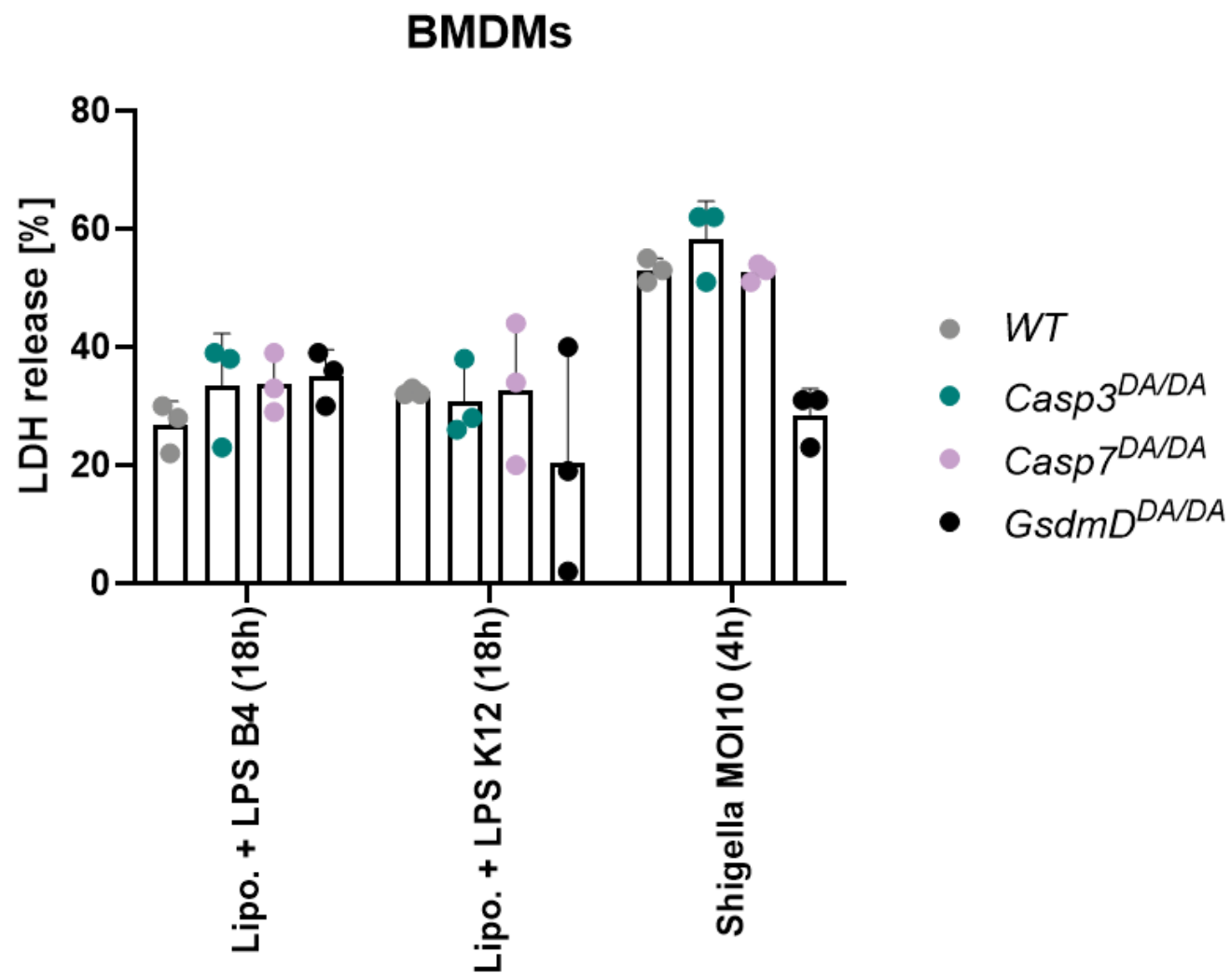

Minor comments:

1. In several figures the authors show the results of technical replicates, precluding statistical analyses. In most cases this is fine, but I feel that Fig 1E would benefit from biological replicates and statistics to support the combined involvement of casp3 and casp7 in intrinsic but not extrinsic apoptosis.

Figure 1A (now **Fig. EV2A**) is changed to “biological” replicates (three independent experiments with the same cell lines), showing the same results (see below).

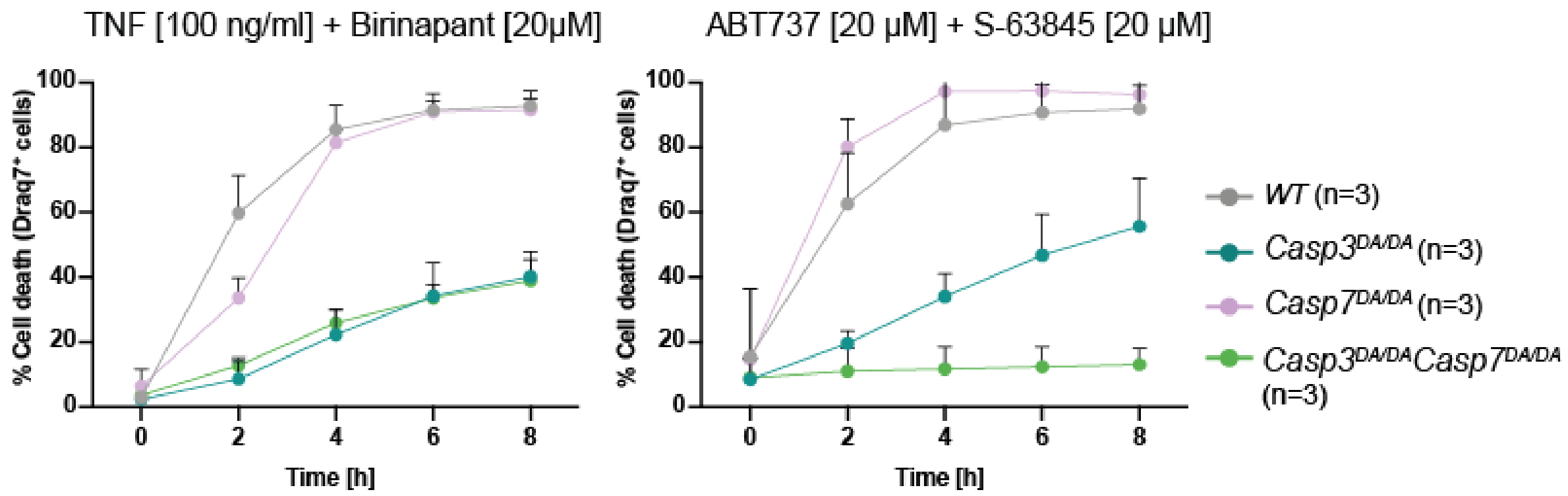

2. Fig 2C: use Ripk3 instead of RIP3.

We changed RIP3 to RIPK3 in Figure 2D (now **Fig. 3D**).

3. In Fig 3B it seems as if the eosin staining of the Casp3DA sections did not work in comparison to the other genotypes. Can you provide better stainings?

New staining was performed and the data are incorporated in new **Fig. 4B**.

4. In Fig 5E it is odd that the F4/80 staining does not show positive cells, as the lungs should have interstitial and alveolar macrophages. Did the staining really work?

We thank this reviewer for raising this point. We also realised that the initial staining of F4/80 was somehow inefficient. New staining was performed and the data obtained are included in the revised manuscript **(now Fig. 6E)**. These data clearly show that the perivascular inflammation in lung of *Casp3$^{DA/DA}$* mice also involve F4/80 macrophages.

5. Fig EV3: In the B panel what is M/ml? In C the pictures do not show anything, please provide better quality pictures. And in D, ideally please provide a positive control for clCasp3 since none of the pictures show positive cells.

In panel B, M/ml referred to the concentration of 1Million sperm/ml (changed to Sperm concentration [1 Mio./ml] in Fig. **EV4B**)

In panel **EV4C**, we provided a higher magnification of the images (see below). We also included SEM images of *WT* vs Casp7$^{DA/DA}$ sperm in new **Fig. 4E** (see below)

Fig. EV4C:

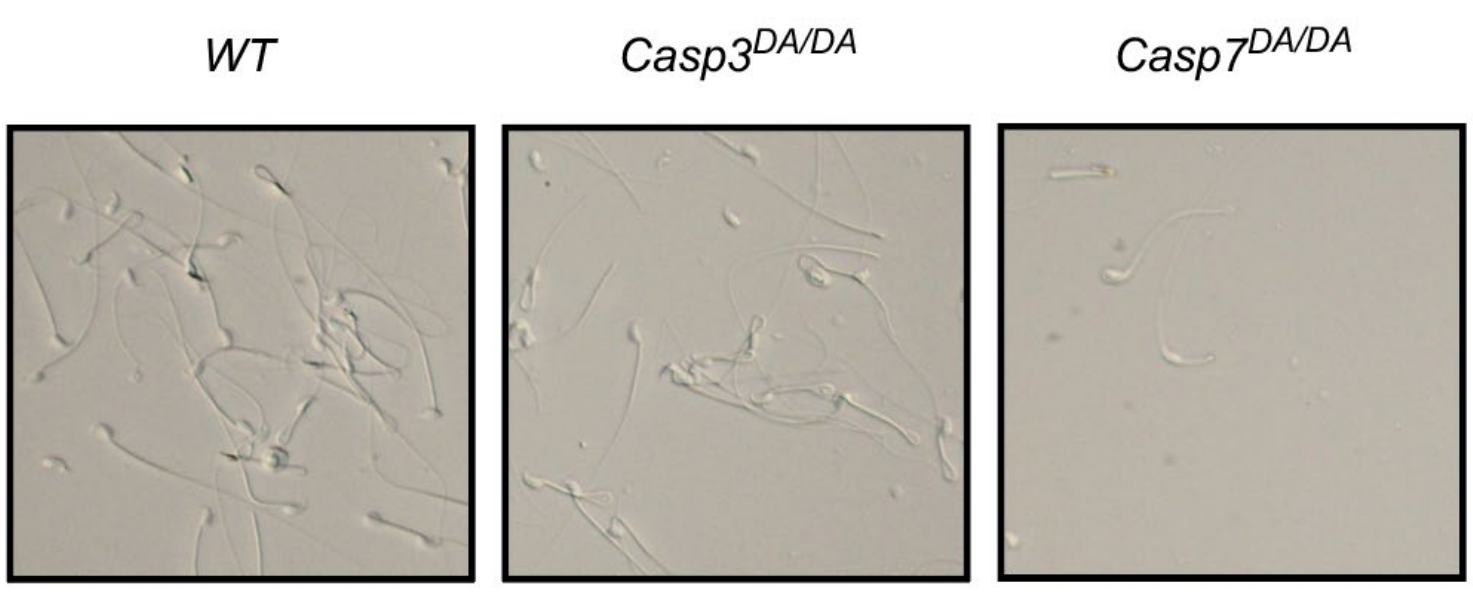

SEM (Fig. 4E):

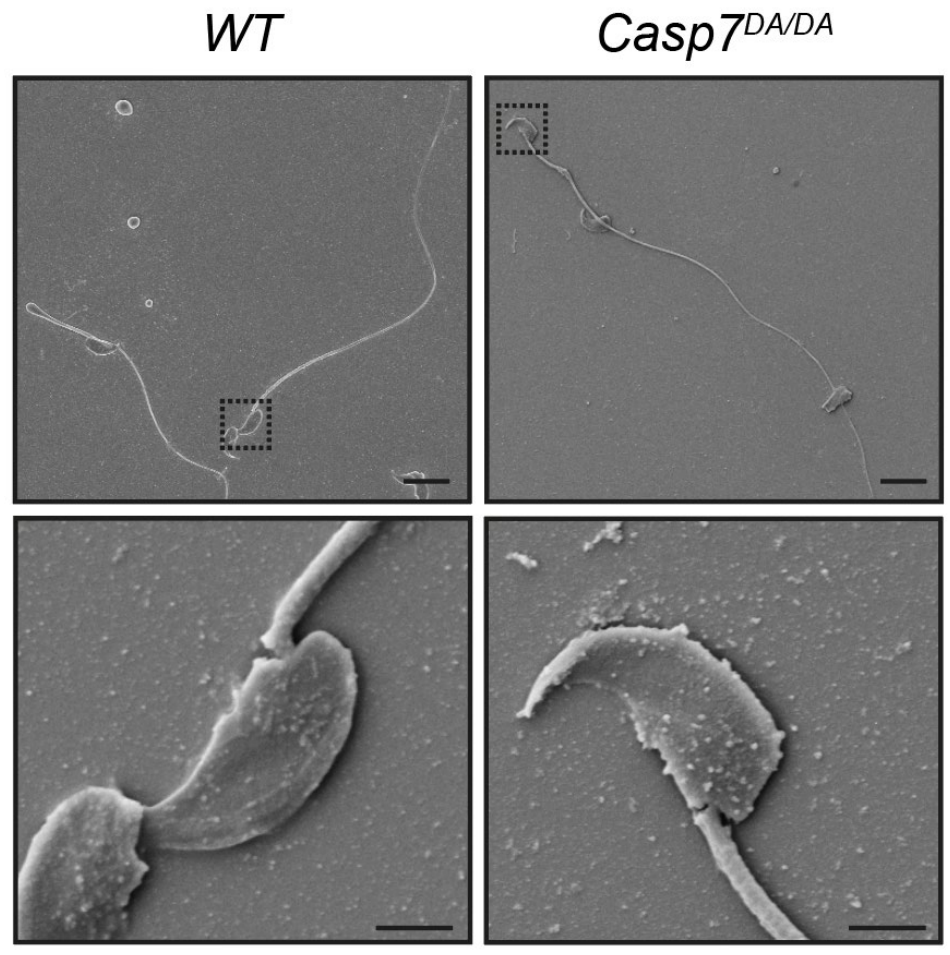

In panel D, we repeated the cl. CASP3 staining for the testes sections. Now, cells positive for cl. Casp3, marked with an arrow, can be seen. Independently, we performed TUNEL staining detect apoptotic cells in the testes (see below). These data confirm that the observed infertility of male *Casp7^DA/DA* mice are not due to an increase in apoptosis in testes.

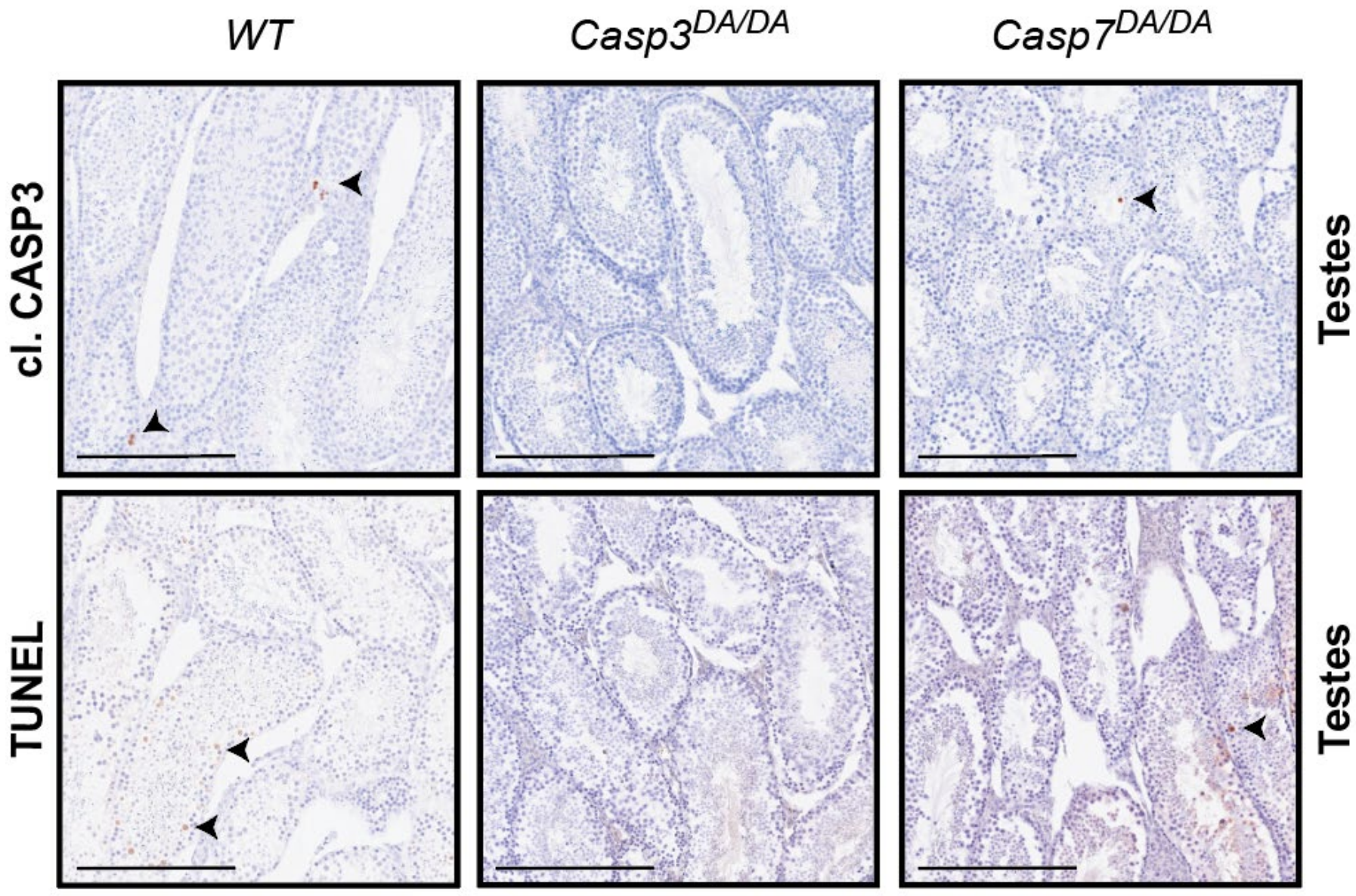

6. Fig EV4B: it seems that the Casp3DA/DA cervical lymph node is full of neutrophils. This is not mentioned or discussed in the results. The same figure also shows a Bcl6 staining, but this is not clear. Please provide a better staining.

The labelling of Fig EV4B was unfortunately wrong. The pictures presented showed staining of F4/80, but not Ly6G. We apologise for the confusion. We repeated the staining for F4/80 and Ly6G and the new data are now incorporated in the revised manuscript (see below). The lymph nodes do not show any neutrophil accumulation.

We also repeated the staining for BCL6 in lymph nodes of WT mice and used a higher magnification of images to show a clearer staining.

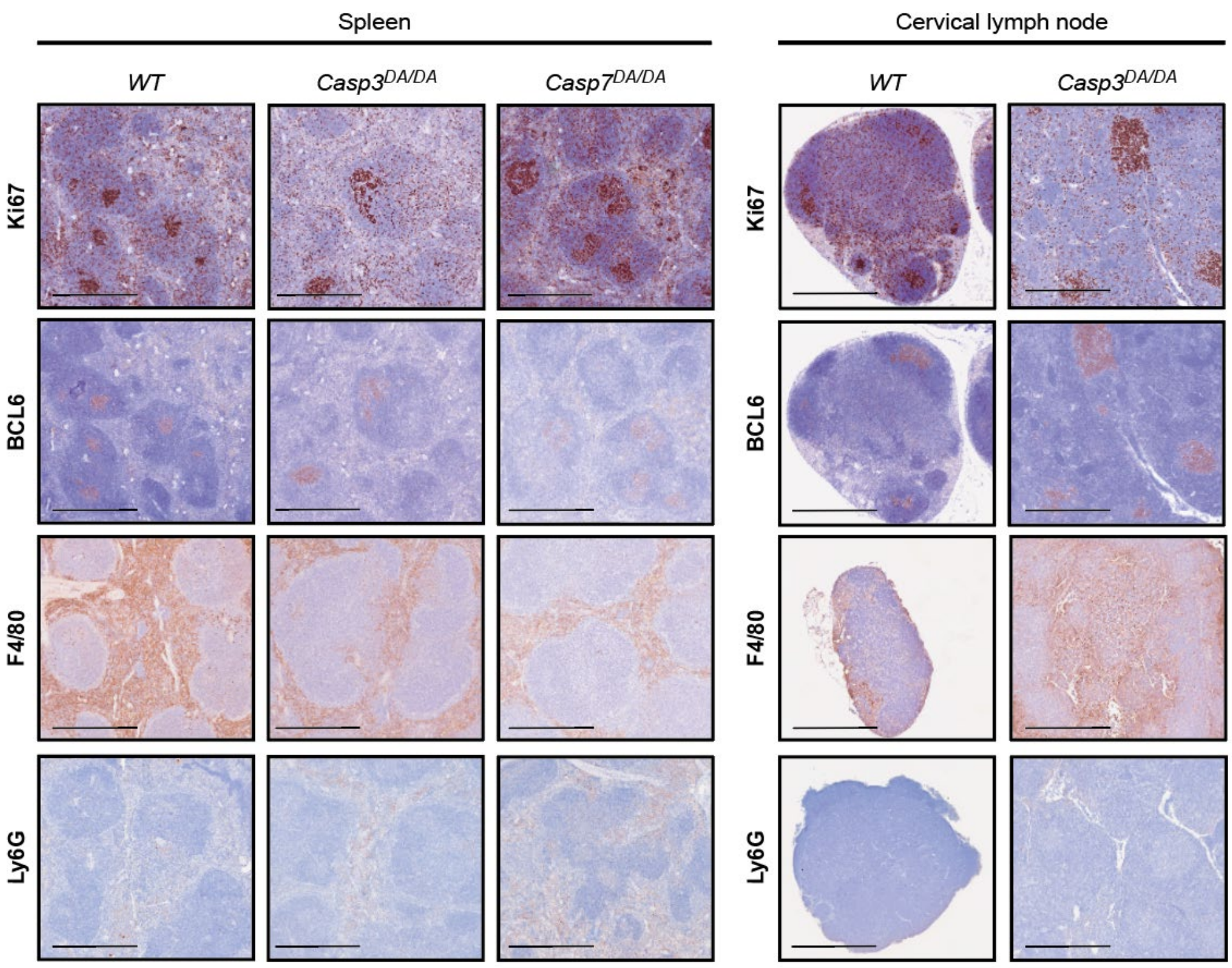

7. Based on Fig 4D, the authors claim 'increased fractions of CD95+ and MHC class II+ B cells and CD69+ and CD44+ memory T cells'. However, not all of these statements are supported by statistically significant differences.

We agree that the wording is misleading and therefore rephrased it: “$CD95^+$ and MHC class II^+ B cells and $CD69^+$ and $CD44^+$ memory T cells appeared slightly increased in *Casp3*$^{DA/DA}$ mice.

8. Also in figure 4D it is odd to only look at proportions of the immune cells and not absolute counts, especially considering the lymphoproliferative phenotype. With only proportion data it is hard to conclude that there are more activated cells when it could just be that the excessive proliferation is altering the proportions.

We provided some information about the total numbers of the immune cells and included the relevant results in **Appendix Fig. S1** (see below). As *Casp3*$^{DA/DA}$ mice develop splenomegaly, nearly all absolute numbers of immune cells were significantly higher in *Casp3*$^{DA/DA}$ mice.

Appendix Figure S1

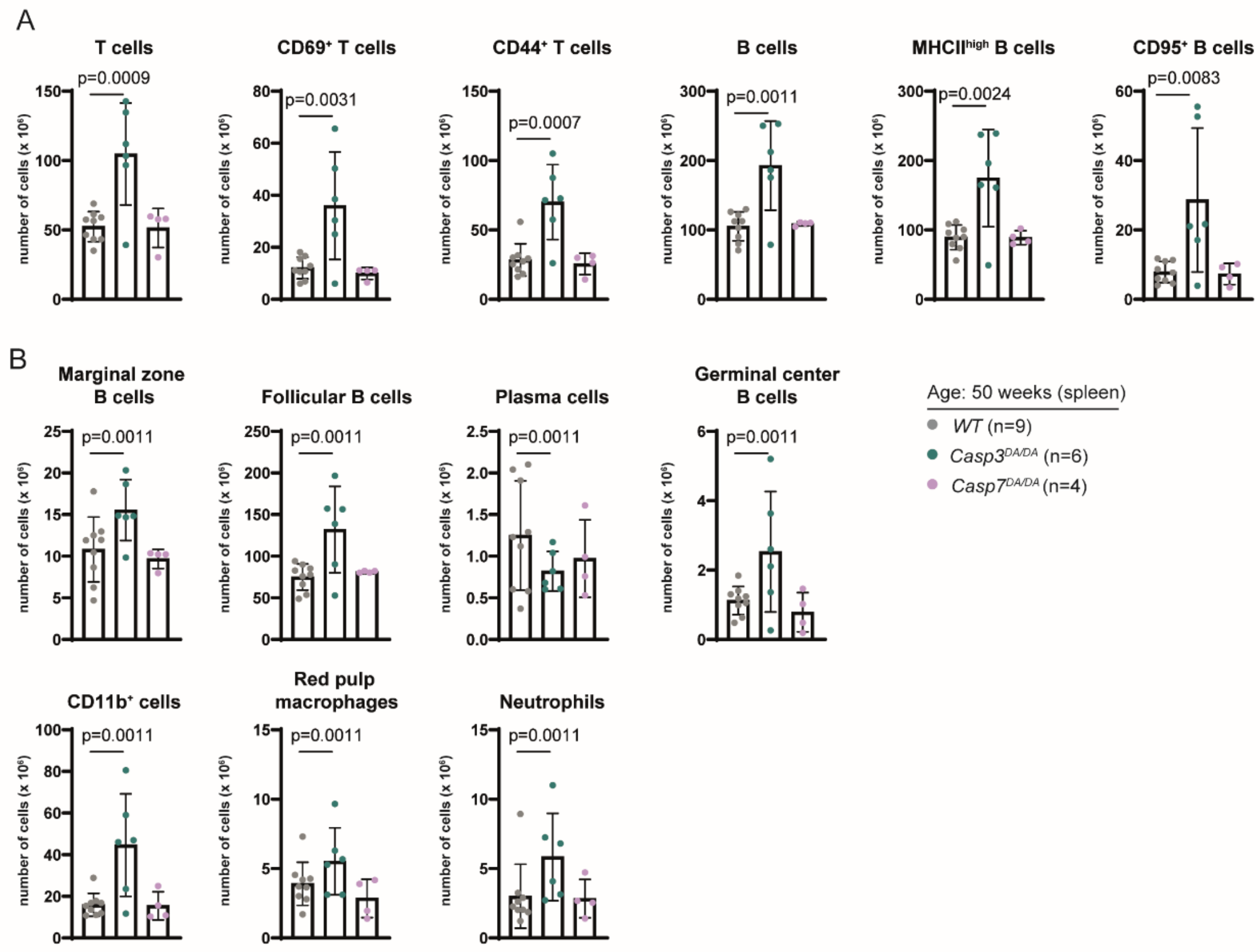

9. In Fig 4E, please provide separate data for CD4 and CD8 T cells to show whether the higher CD4/8 ratio is caused by increased CD4 or decreased CD8 T cell counts.

We thank the reviewer for this note. Our data show higher total numbers of T cells in *Casp3*$^{DA/DA}$ mice. Whereas total CD4^{+} numbers were significantly increased in *Casp3*$^{DA/DA}$ mice, we could not detect any significant elevation of total counts of CD8^{+} T cells. Therefore, increased CD4/CD8 ratio is due to an increase in CD4^{+} T cell numbers, which is in line with increased percentages of Ki67^{+} CD4^{+} T cells in *Casp3*$^{DA/DA}$ vs WT spleens (see below). The new data is incorporated in **Fig. 5F-G.**

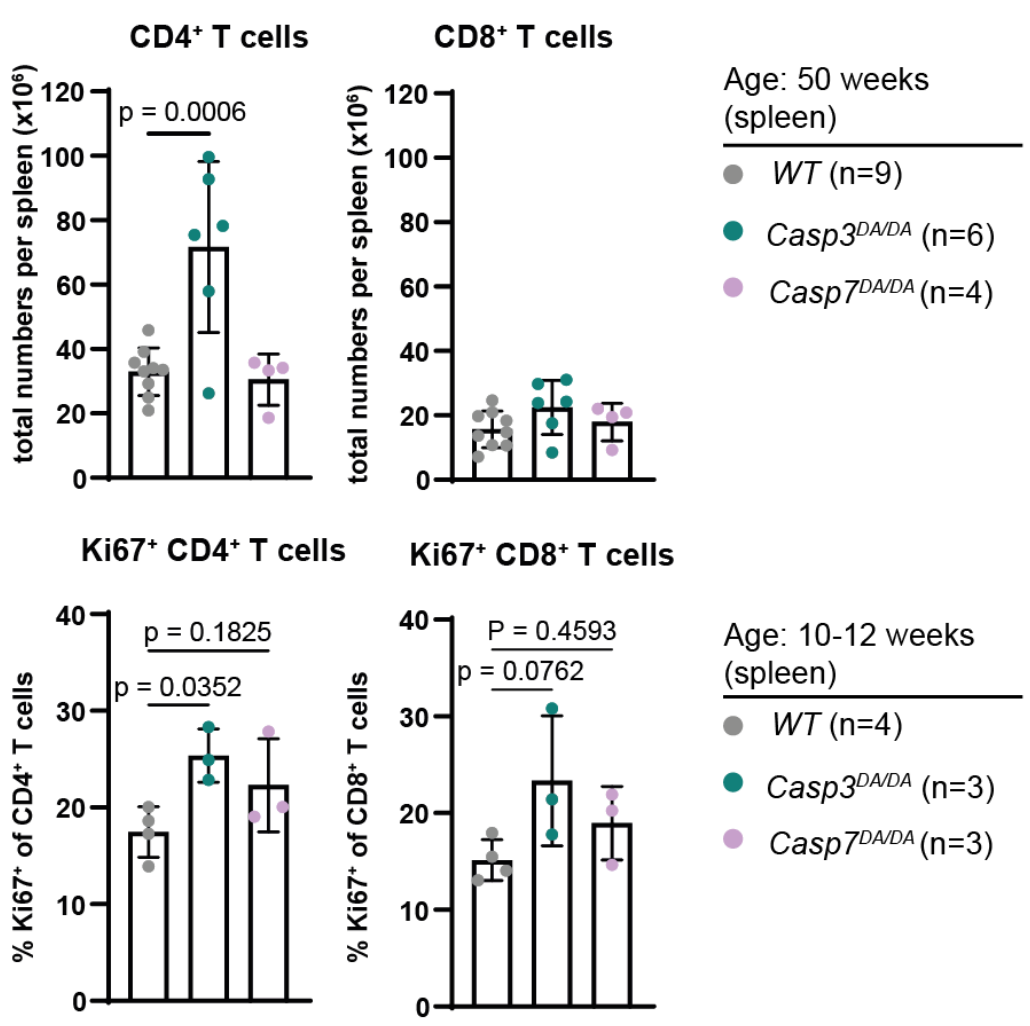

10. Fig EV4C: macrophages, not makrophages.

Labelling of the figure is changed in the revised manuscript.

11. Since the claimed upregulations in Casp3DA/DA cells of RIG-I, STAT1 and IRF3 in Fig 5C are not super convincing, and since they are not accompanied by data showing their Casp3-dependent cleavage, I would argue to omit these data and to solely base the IFN signature on the more convincing cGAS, MAVS and P-STING data in these figures.

We rephrased, accordingly.

Referee #3:

Summary

Sieg and colleagues describe knock-in mice expressing cleavage-resistant CASP3 (D175A) and CASP7 (D198A), dubbed CASP3^DA/DA and CASP7^DA/DA. They confirm CASP3 as the dominant executioner in apoptosis and show that double mutants die between E13.5 and weaning, mirroring prior CASP3/7 DKO reports. Adult phenotypes include CASP7-linked spermatogenesis defects and CASP3-driven lymphoid hyperplasia. While the study provides a useful genetic resource and highlights some non-redundant roles, it is largely confirmatory. The absence of substrate identification or genetic rescue limits mechanistic insight, making this more of a descriptive dataset than a conceptual advance. Minor points follow.

We thank this reviewer for his/her critical view of our data considering the conceptional advance provided by our study. However, we respectfully ask the reviewer to reconsider the relevant literature, particularly findings derived from caspase gene knockout models.

CASP3-deficient 129/SvJ mice were originally reported to die during the perinatal period due to neuronal defects. However, when these mice were backcrossed onto a C57BL/6 background for ten generations, CASP3 deficiency was no longer associated with lethality (Leonard et al 2002). Furthermore, 129/SvJ mice with CASP7-deficiency were initially reported to die during embryonic development (Zheng et al 1999). Later, these mice seemed to appear normal (Lakhani et al 2006). Importantly, earlier studies of CASP3- and CASP7-deficient mice did not report defects in spermatogenesis or lymphoproliferation. Moreover, a rigorous comparative physiological analysis of these two executioner caspases has not been performed, largely due to substantial differences in the genetic backgrounds used to generate these models.

In light of these considerations, we believe it is not fair to call our study as merely "confirmatory" just because *Casp3*$^{DA/DA}$/*Casp7*$^{DA/DA}$ mice embryonic lethality resembles that reported for *Casp3*$^{-/-}$/*Casp7*$^{-/-}$ mice. We believe our findings provide new insights that extend beyond prior observations using caspase gene knockout mice.

Note to authors: The resolution of all images (western, H&E, etc.) have been downsampled in the PDF. This makes it difficult to interpret any histological images and appreciate more subtle cleavage patterns, such as the ASM blot in Fig. 3F. In particular, Fig. EV3C displays as a grey box. Please remove compression on images.

We are thankful for this very helpful note and will carefully control the image quality after submission/file conversion of our revised manuscript.

Minor points

• Authors should indicate where cleavage-specific antibodies to CASP3 (Asp175) and CASP7 (Asp198) were used. This would be helpful in demonstrating the specificity of the Casp3DA/DA/Casp7DA/DA point mutations (i.e. Fig. 1B).

The anti-CASP3 and CASP7 antibodies used in our study detect caspase zymogen and cleaved caspase bands (antibody for full length and cleaved caspase were combined).

• In EV1B, EV2B, and EV2C: Are different blot exposures being spliced together and presented on a single panel? On close inspection, the dashed lines seem to separate blots of different exposures. If so, this is misleading. Please use the same exposure for each antibody combination. As currently displayed, there seems to be an increase in CASP7 total protein in the left three lanes (recombinant CASP9 treated) versus unstimulated in EV1B.

We apologize for the confusion. In all Figures, cropped blots always originate from the same exposure. The uncropped blots for each respective blot can be found in the Source data of the main figures and the Western Blot source data of EV Figures.

• The authors do not formally show lack of cell death in Casp3DA/DA/ Casp7DA/DA embryos. A TUNEL stain in conjunction with cleaved CASP3 staining in WT vs Casp3DA/DA/Casp7DA/DA embryos would strengthen the claim that "CASP3 and CASP7 provide redundant functions during embryonic development, likely be executing apoptosis."

We agree with this reviewer and rephrased the respective description accordingly to "*during embryonic development the activation of CASP3 or CASP7 seemed to act redundantly, either by controlling apoptosis or other cellular processes which needs further careful investigation*". In response to this point, we examined CASP3 cleavage and TUNEL staining in WT and *Casp3$^{DA/DA}$/Casp7$^{DA/DA}$* embryos (E13.5). Whereas cleaved CASP3 was specifically evident in WT embryos, TUNEL staining by using two different TUNAL kit assays appeared rather less specific without providing useful results (see below).

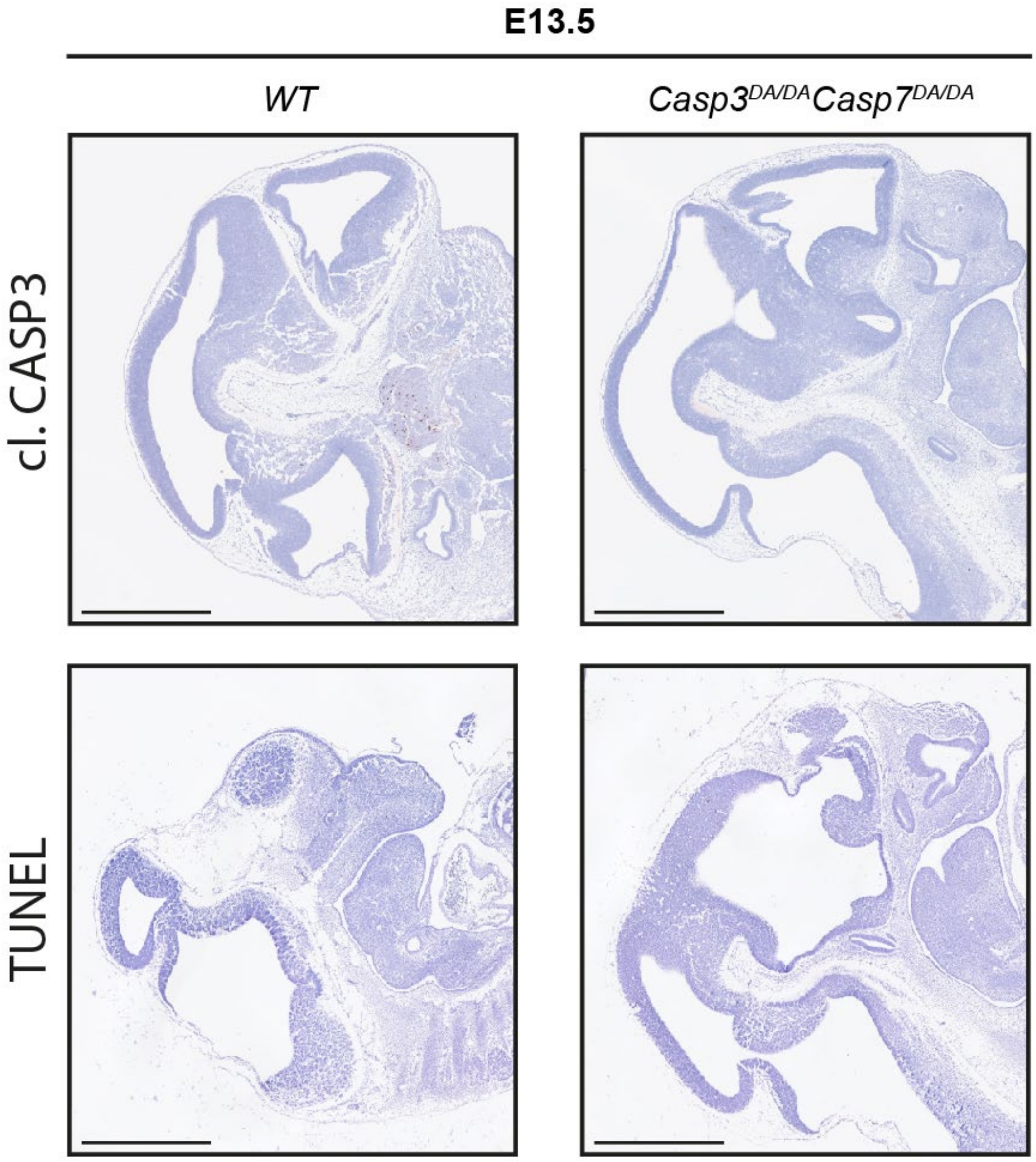

• In Fig. 3F, the unspecific band detected by CASP3 falls at roughly the same molecular weight as cleaved CASP3. It may be more informative to use the cleavage specific CASP3 antibody here to demonstrate that cleaved CASP3 is not being up-regulated in CASP7DA/DA animals

As already mentioned, the antibodies used for the detection of CASP3/7 detect both the zymogen and cleaved/activated caspases. For more clarity, we included a WB using the

cleavage-specific anti-CASP3 antibody alone and included lysates from MEFs, treated with ABT737 + S-63845 (+) as a positive control. The blot demonstrates that the detected band is an unspecific antibody reaction (see below). We included the WB in **Fig. EV4E**.

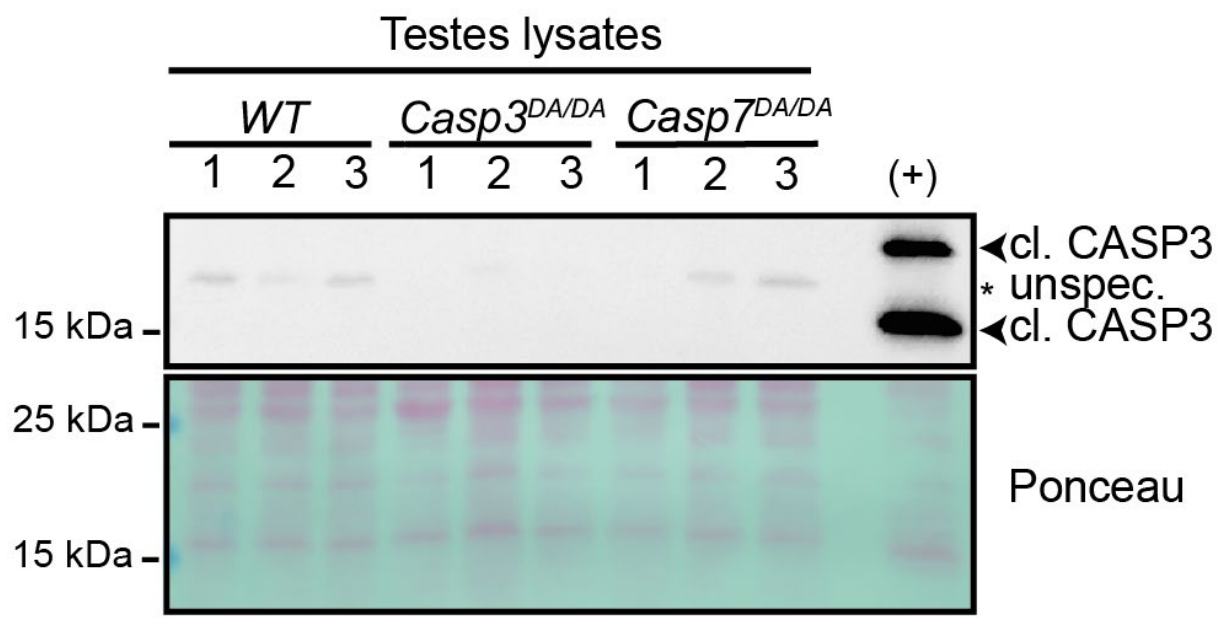

• A TUNEL stain in CASP7DA/DA testes sections would bolster the interpretation that the reduction in sperm is not due to apoptosis

As recommended, we performed TUNEL staining in the testicular sections (see below, new **Fig EV4D**) showing relatively similar appearance of TUNEL-positive cells in *WT* and *Casp7*$^{DA/DA}$ testes.

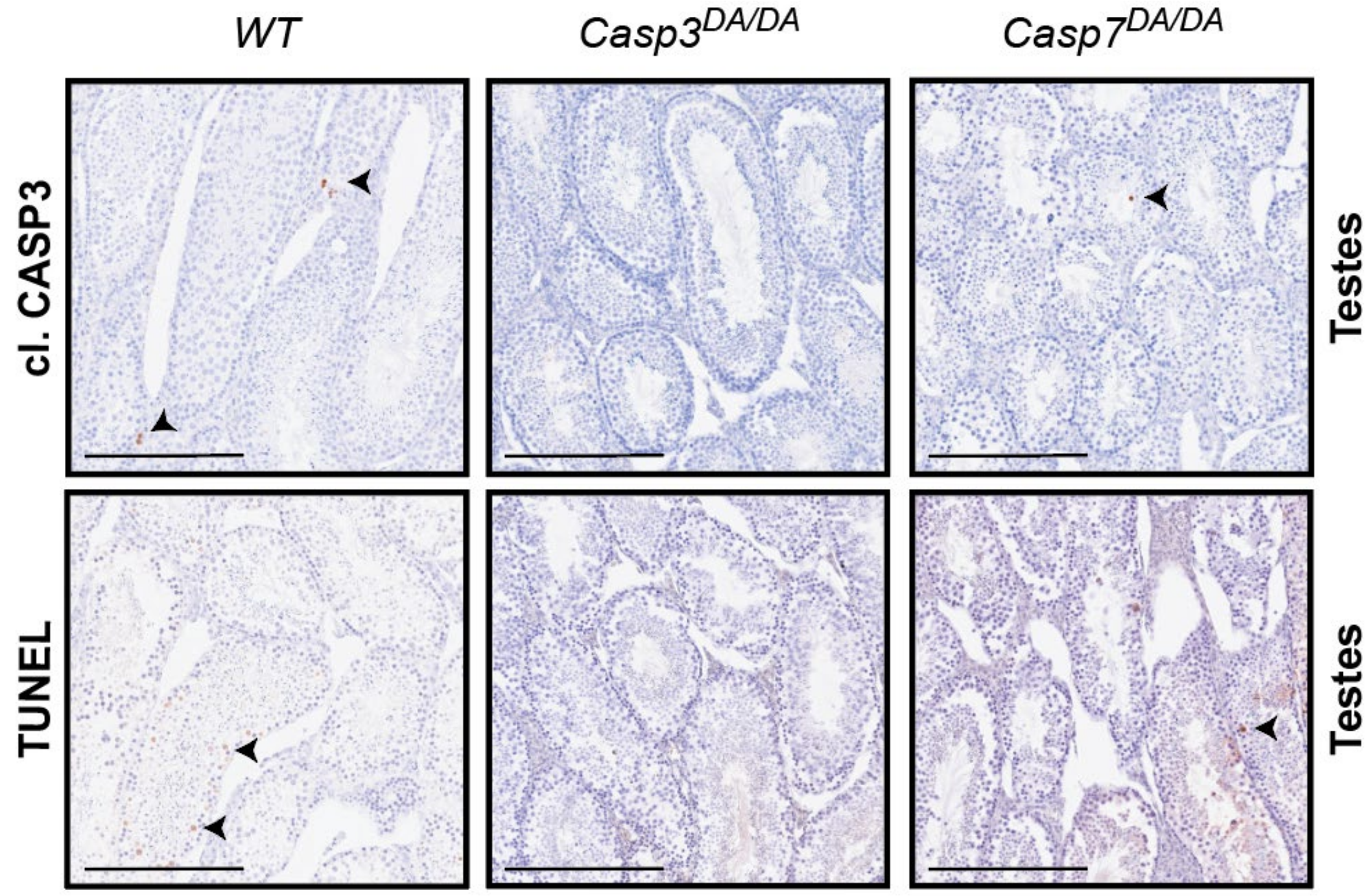

• Fig. 3C is missing a scalebar

Scale bar is included in the revised manuscript.

• In Fig. 4D and E, the authors note increased fractions of CD95+ B cells, MHCII high B cells, and CD69+ T cells. They also note increased CD4 to CD8 Y cell ratios in spleen and cervical lymph node. However, none of these measurements seemingly reach statistical significance, based on the figure panel itself. The authors should clarify this interpretation.

We agree with this reviewer and appreciate this point. In the revised manuscript, we changed the description of our data and stated that "CD95$^+$ and MHC class II$^+$ B cells and CD69$^+$ and CD44$^+$ memory T cells appeared slightly increased in *Casp3*$^{DA/DA}$ mice". We also included absolute counts of CD4$^+$ and CD8$^+$ T cells in the spleens (new **Fig. 5F**). There were significantly

more CD4$^+$ T cells in the spleen of *Casp3$^{DA/DA}$* mice, while CD8$^+$ T cell numbers remained similar, leading to the increase in the CD4/CD8 T cell ratio in the spleen.

• Fig. EV4D has a floating "h" on the y-axis in Pro/Pre cells panel

The floating “h” was removed.

• Please indicate where drugs (Birinapant, TNF, s-63845, ABT-737, LPS, Nigericin, etc) were purchased from

This information is included in the revised manuscript.

Referee #4:

This is an interesting study that identifies distinct requirements for caspase-3 and caspase-7 in vivo for the suppression of Type I IFN signaling in the lymphoid compartment and spermatogenesis, respectively. Processing-defective Casp7DA mice exhibited spermatogenesis defects, whereas Casp3DA mice exhibited splenomegaly and perturbation of the ratios of CD4/CD8 T-cells and other lymphoid abnormalities, potentially related to imbalanced Type I IFN signaling.

We thank this reviewer for very helpful advices and all details to improve our explanations.

1. In Figure 1E and 1F it is shown that BH3 mimetic-induced cell death is greatly suppressed in the Casp-3DA/Casp-7DA MEFs, at least at the endpoint of 8h that is shown. However, given that BAX/BAK channel opening is typically considered the point in commitment in cell death, with caspase-3/7 activation only necessary to produce the apoptotic phenotype, can the authors clarify whether the Casp-3DA/Casp-7DA cells survived BH3 mimetic treatment for several days, or whether these cells simply died with a kinetic delay. Furthermore, I feel that it's important to clarify what the phenotype of cell death was when Casp-3DA/Casp-7DA MEFs did die, presumably this was very different to the WT phenotype. Some high resolution confocal images would be useful here.

This is indeed an important point that deserves further clarification. In the revised manuscript, we included extensive analysis summarised in new **Fig. 2**.

Specifically, we included additional data monitoring cell death for up to 4 days (new **Fig. 2A,** see below).

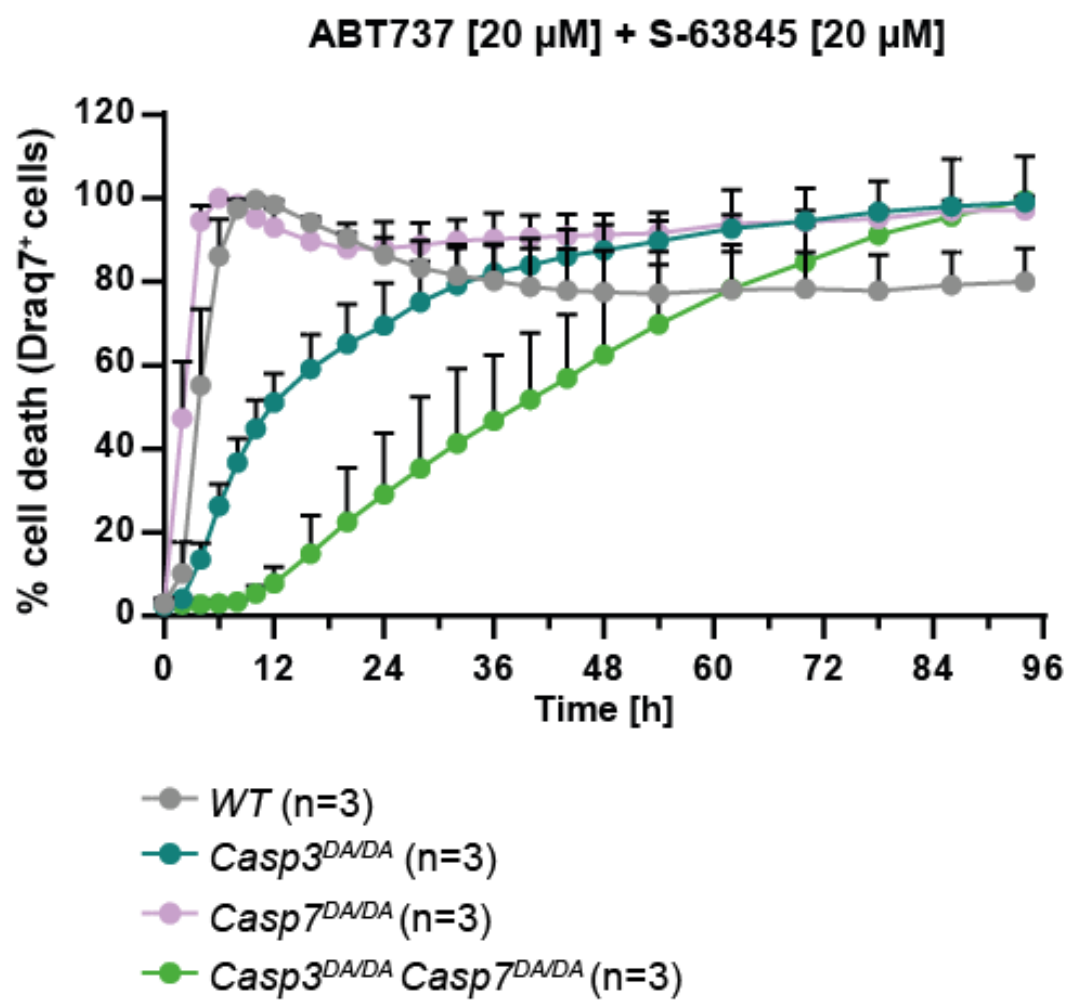

We analysed nuclear fragmentation and TUNEL staining of cells treated with BH3 mimetics (new **Fig. 2B**).

We also performed detailed scanning electron microscopy (SEM) (new **Fig. 2C-D**)

We additionally analysed cell viability based on cellular metabolic activity by using CellTiter 96® AQueous One Solution Cell Proliferation Assay (MTS) (new **Fig. EV2D**)

Our data collectively indicate that CASP3 is the main determinant of the apoptotic death in response to BH3 mimetics. Specifically, in the absence of active CASP3, cells showed a delayed kinetic of cell death (Draq7 positivity), lack of formation of apoptotic bodies and fragmentation of the nucleus/DNA (lack of TUNEL staining). Nevertheless, metabolic activity decreases after 24h of BH3 mimetic treatment in both *Casp3$^{DA/DA}$* and *Casp3$^{DA/DA}$Casp7$^{DA/DA}$* cells, most likely, upon mitochondrial damage induced by BH3 mimetics. In contrast to *Casp3$^{DA/DA}$Casp7$^{DA/DA}$* cells, *Casp3$^{DA/DA}$* cells expressing WT CASP7 showed membrane blebbing, without progressing to complete cellular fragmentation involving nucleus. These

observations indicate that CASP7 induces cellular shrinkage but is unable to efficiently execute apoptotic death.

2. In Figure 2, there is no positive control for the pMLKL antibody used. This should be provided as a supplementary figure.

In response to TNF/birinapant, p-MKLK was demonstrated in Fig. 2A. In response to this point, we also included a WB of the cellular lysates used in our analysis in old Fig 2B (now **Fig 3B**) and included a positive control for p-MLKL (MEFs treated with TNF, birinapant and emricasan) (new **Fig EV3C**) (see below).

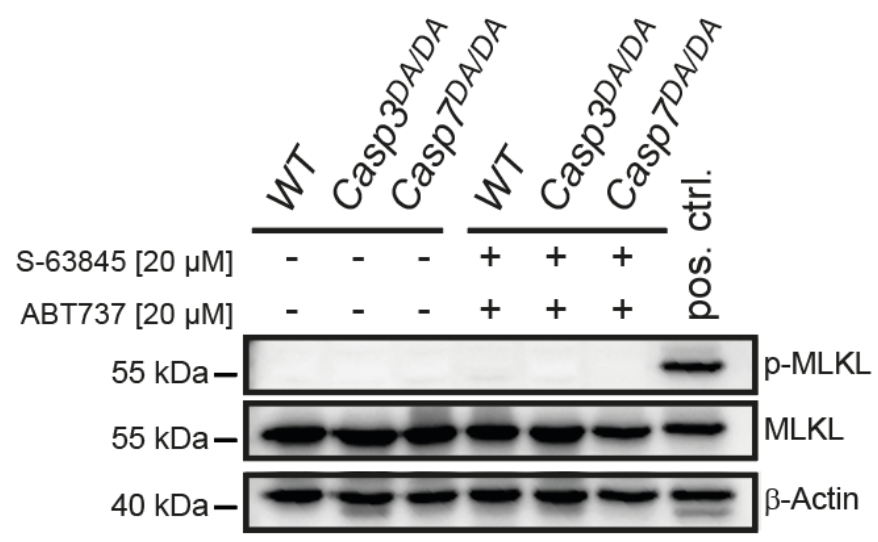

3. In Figure 2F GSDMD and possibly also GSDME proteolysis should be examined, as these are the key caspase substrate driving pyroptosis.

We included data showing GSDMD cleavage and IL-1β secretion in new **Fig. 3E** and **Fig. 3F**. We could not detect GSDME cleavage in these cells as GSDMD-induced pyroptosis is a much more efficient and rapid death process without involving GSDME. This is clearly demonstrated in BMDMs expressing cleavage mutant GSDMD(DA). Only upon blocking GSDMD-induced pyroptosis, BMDMs accumulated cleaved GSDME. These data are now included in new **Fig. EV3G-H** (see below).

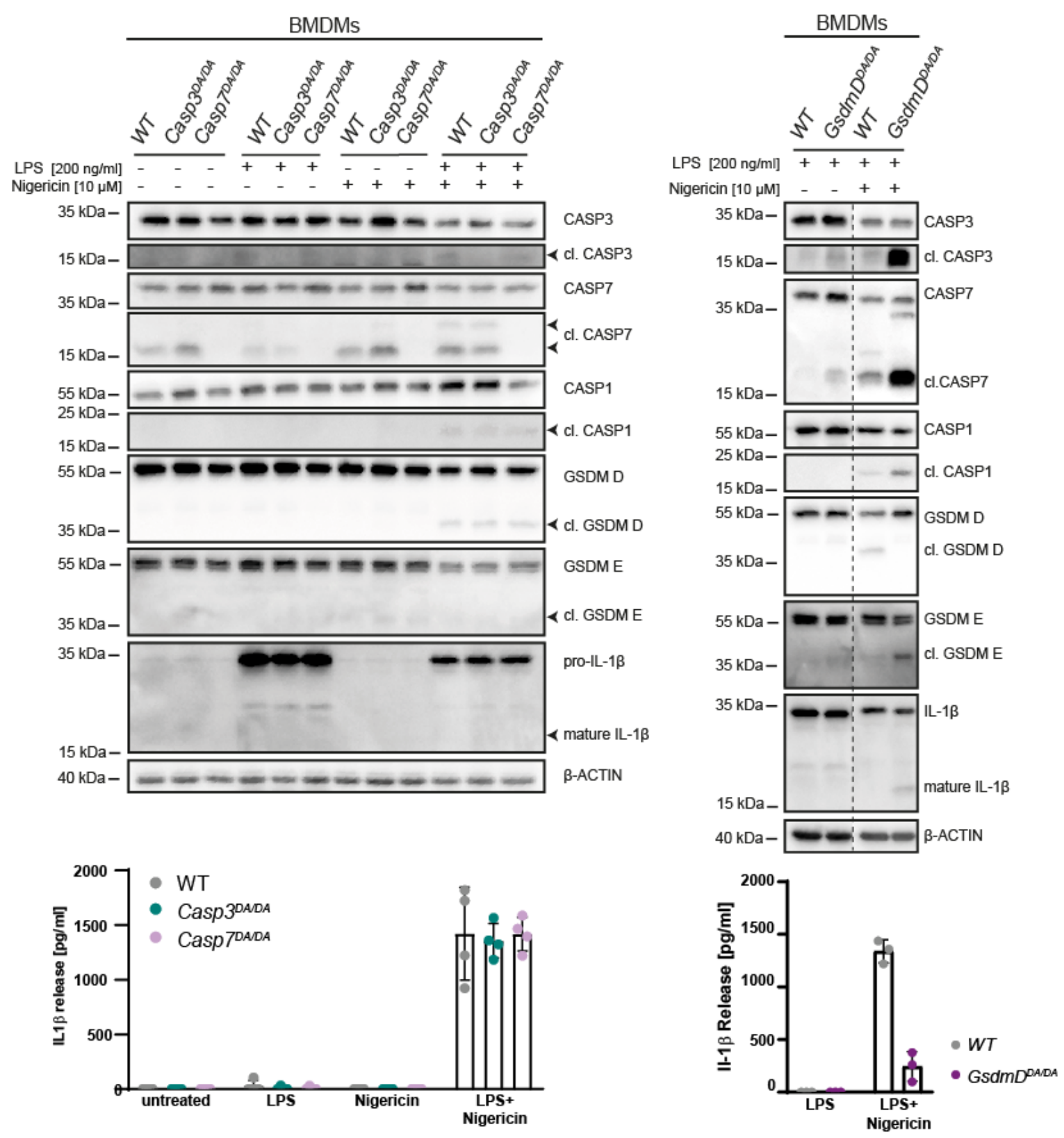

4. The ASM cleavage products depicted in Figure 3F (testicular lysates) is not convincing and could be improved. Perhaps the authors could probe the MEF lysates used in Figure 1 to determine ASM cleavage status in response TNF/Birnapant as well as ABT/S6 treatment in the various genotypes.

Unfortunately, we were not able to produce better staining for cleaved ASM in mouse testicular tissue lysates, likely due to the lack of a better specific anti-ASM antibody and/or the low amount of the cleavage product accumulating in the healthy testis. Independently, we realised that the insufficient quality of the cleaved ASM is at least partly due to the fact that the resolution of our submitted Figures has been somewhat downsampled, likely upon PDF conversion (this was pointed by the **Referee#3**). We will make sure that our revised Figures have the best resolution/quality, hopefully addressing this concern adequately.

Notably, previous studies demonstrating CASP7-mediated ASM cleavage were performed after acute caspase activation (e.g. FlaTox or TNF exposure) (Nozaki et al 2022 Nature; Edelmann et al 2011 EMBO J) which then led to an efficient cleavage of ASM. In contrast to these experimental conditions, mouse testis does not comprise high-level caspase activity under normal/physiologic condition (**Fig. EV4D**) and exhibit most likely therefore less ASM cleavage.

We also tried several experimental approaches to monitor ASM cleavage in MEFs after induction of caspase activity. The results obtained were, however, difficult to interpret as anti-ASM antibody(ies) detected several bands in cultured MEFs, also in ASM-deficient MEFs (see below). Based on these observations, we decided to rephrase the respective paragraph in our revised manuscript and particularly placed less emphasis on the involvement of CASP7-mediated ASM cleavage in male fertility, as our data by its own do not convey a proof for such conclusion. This notion was basically drawn upon previous studies demonstrating CASP7-medaited ASM cleavage and independent observations frequently indicating that sphingolipid metabolism controls male fertility.

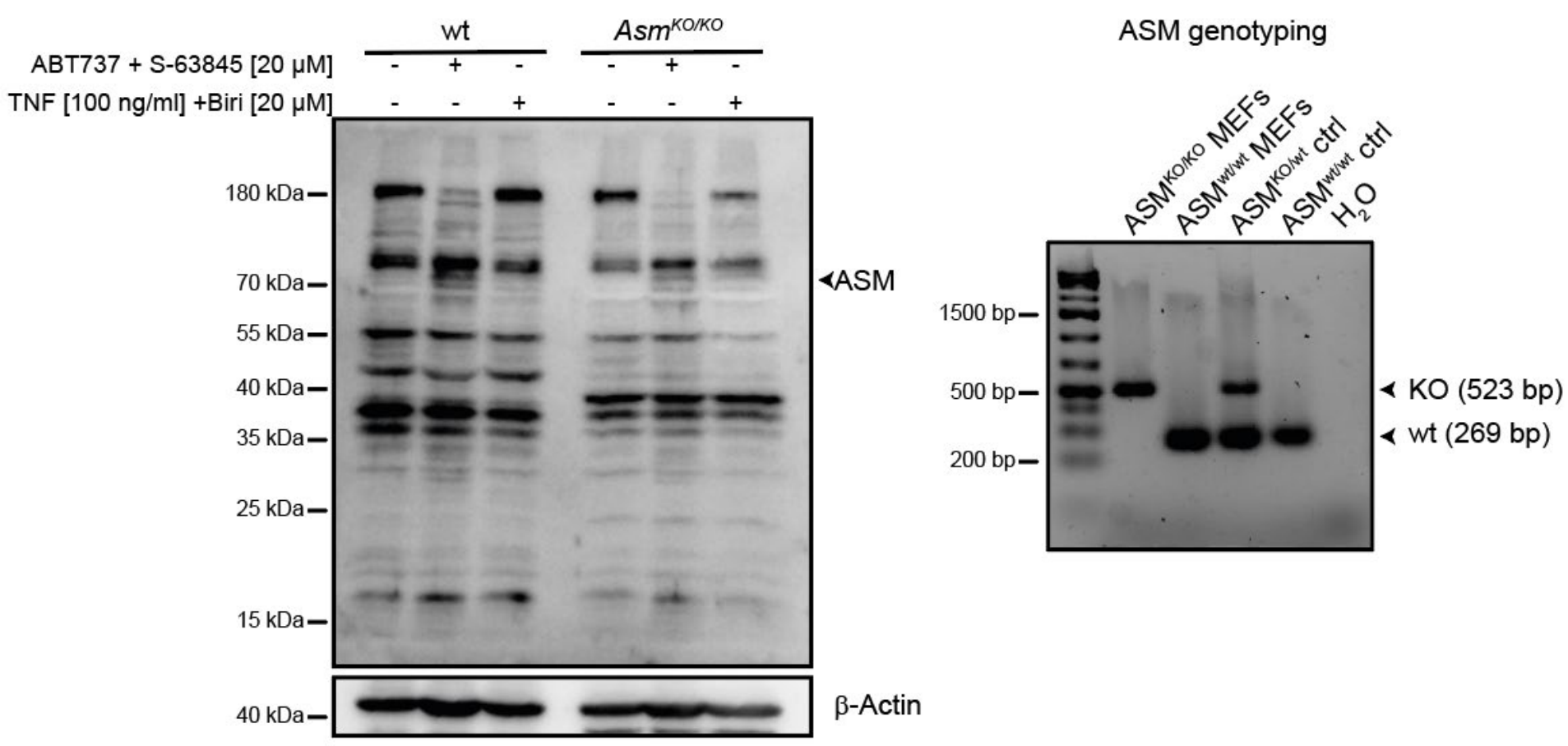

5. How caspase-7 may become activated during spermatogenesis is not discussed. Is it that caspase-3 cannot cleave ASM or that it is not activated during spermatogenesis? Can enough sperm be obtained from the various genotypes to promote apoptosis using BH3-mimetics to determine whether (a) caspase-3 and caspase-7 become proteolytically processed in sperm in response to a pro-apoptotic stimulus and (b) is ASM proteolytically processed in WT versus Casp-3DA or Casp-7DA sperm under these conditions?

Our new data summarised in new **Fig. 2** (see response to major point 1 of this reviewer) indicated that CASP7 induces cellular shrinkage but is unable to efficiently execute apoptotic death. It is important to note that such process have been frequently observed during spermatogenesis and have long been considered as apoptosis. Studies, initially performed in Drosophila, have already demonstrated caspase-mediated spermatid individualisation

(cytoplasmic shedding) without apoptotic death. Our new data examining the expression level of CASP3 and CASP7 in different mouse tissues (3 mice) (new **Fig. 4G**, see below) showed that whereas both CASP3 and CASP7 were strongly expressed in tissues such as spleen and colon, CASP3 was barely detectable in testes. In contrast, CASP7 was strongly expressed in testes, but was barely detectable in brain or kidney. These data are completely in line with the data derived from tissue-specific atlas of mouse protein expression (Huttlin et al 2010) and indicate that the dependency of testicular tissue on CASP7 is due to the lack of CASP3.

Thus, our data indicate that testicular tissues, which barely express CASP3, respond to upstream pro-apoptotic cascades just by activating CASP7, which in turn induces membrane blebbing leading to cellular shrinkage required for successful spermatid maturation/individualisation, without involving apoptotic cell death. This notion is completely in line with the data obtained in BAX-deficient mice (Knudson et al 1995), which also developed male infertility. Collectively, these observations indicate the mitochondrial apoptotic pathway in testes leads to activation of CASP7 (there is no CASP3 expression), which is required for successful spermatogenesis.

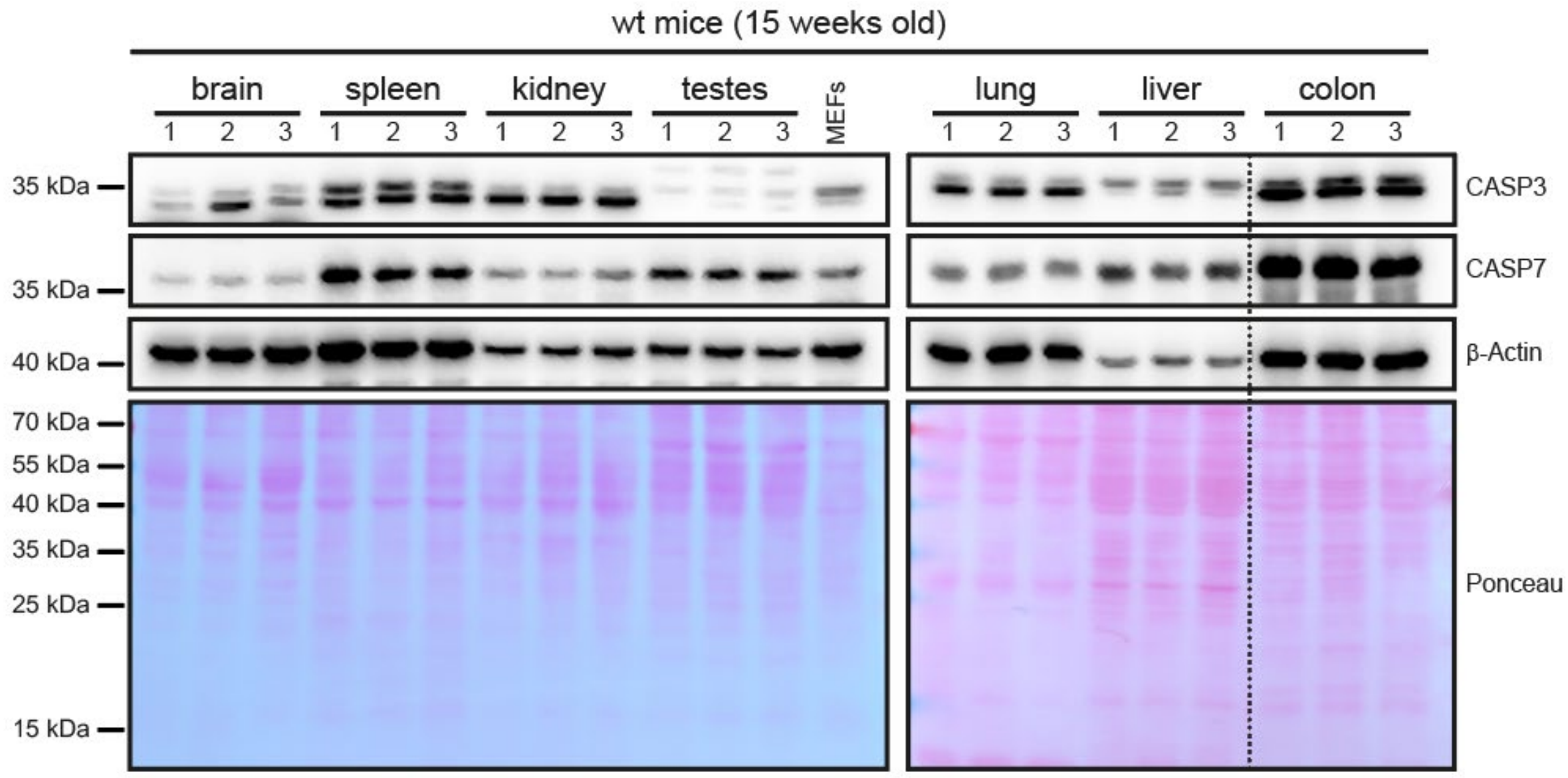

Independently, we were not able to interfere with sperm motility upon caspase inhibitor treatment (see below), suggesting that caspase activity in mature sperm is unlikely involved in their functionality. As already stated, *Casp7$^{DA/DA}$* mice hardly produce sperm and we could not provide additional experiments with isolated sperm. Based on histologic analysis of testes and epididymis, it seems that CASP7 is required for the final differentiation of spermatids, likely by mediating cytoplasmic shedding. As shown in **Fig 4B**, sperm precursors in *Casp7$^{DA/DA}$* mice exhibited rather diffuse and non-differentiated morphology.

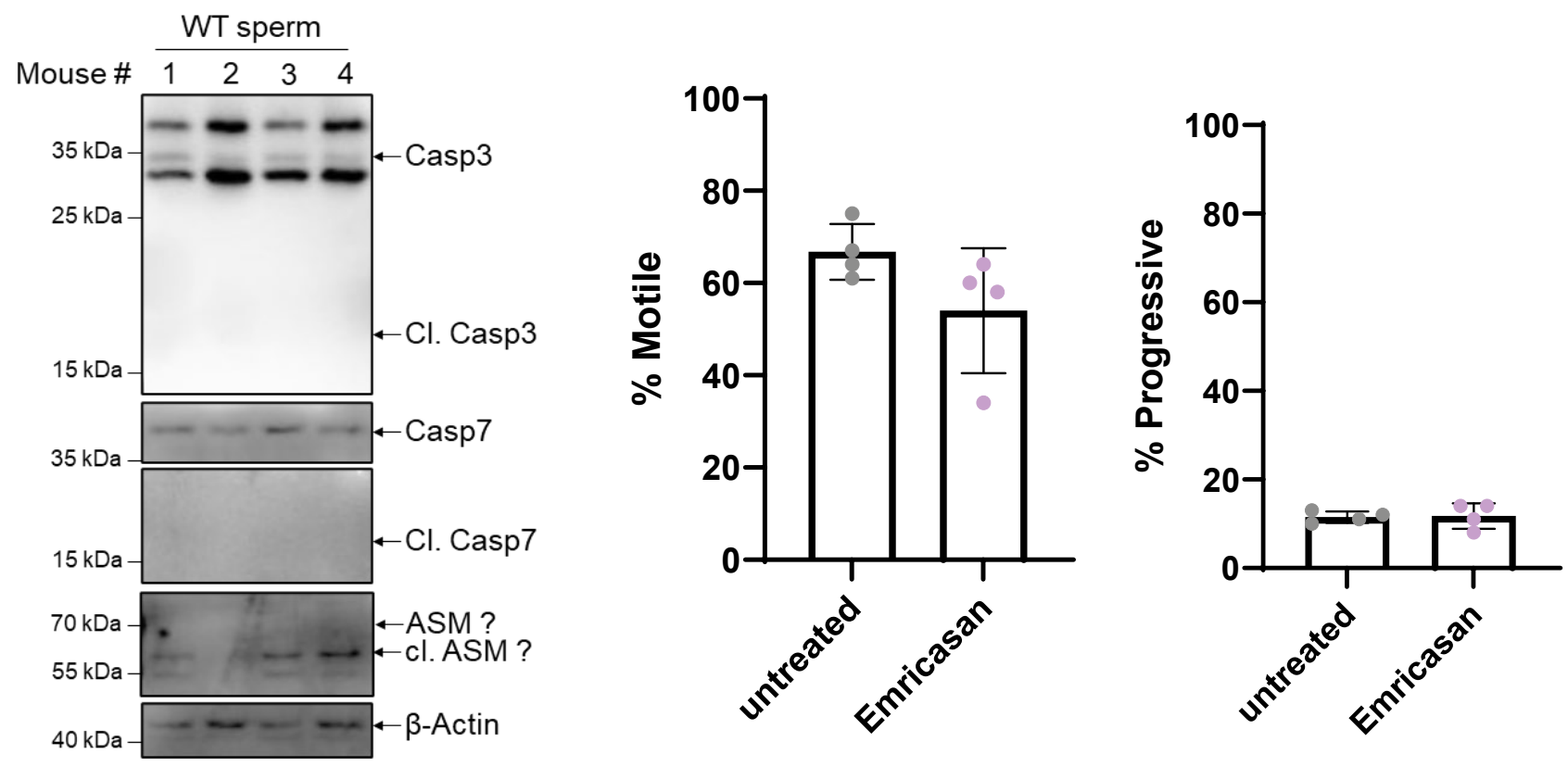

6. Caspase activity has been implicated in T-cell activation in previous studies (e.g. Chun HJ et al., 2002; Salmena et al. 2003; Misra et al. 2008). As an alternative explanation for the lymphocyte hyperplasia observed in Casp-3DA animals, the authors should isolate T- and B-lymphocytes from the various genotypes and examine TCR- as well as BCR-induced proliferation, IL-2/IL-4 production as well as other markers of T- and B-cell activation. Caspase processing activation should also be monitored in response to TCR and BCR stimulation. It seems possible that the previously documented role for caspase-8 activity downstream of TCR activation may promote activation of caspase-3 in T-cells to promote a non-apoptotic endpoint. Do MAVS, cGAS and other components of this pathway undergo caspase-3-dependent proteolysis in response to TCR or BCR engagement in vitro?

This is indeed an intriguing suggestion and we carried out some analyses to address this important point. However, the data obtained did not support the possible involvement of STING signalling, although caspase activation was detected in B and T cells upon antigen-receptor activation (see below).

As recommended, we have isolated T and B cells from the spleens of 10-12 weeks old *WT*, *Casp3*$^{DA/DA}$, and *Casp7*$^{DA/DA}$ mice, labelled them with CFSE and stimulated T cells *in vitro* with anti-CD3/CD28 antibodies and B cells with anti-IgM or ODN1826 (TLR9 activation) for 72 h. Flow cytometric analysis of CFSE signal did not show striking differences in proliferation between the genotypes (see below). We have included the data on T cell proliferation in new **Fig. EV5F**.

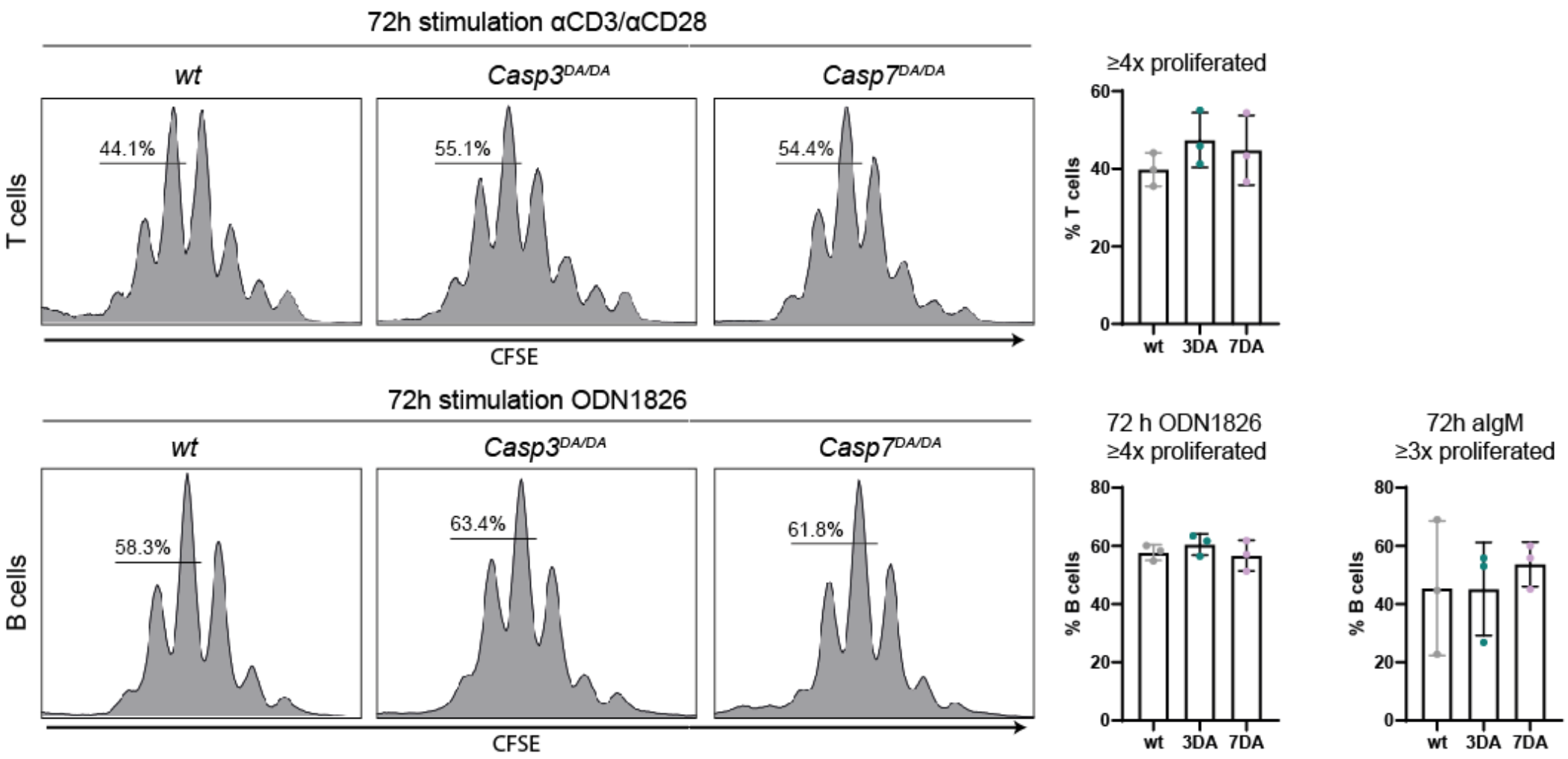

Ex vivo, we also analysed Ki67 expression of stimulated T and B cells and could observe a mild (but significant) increase in Ki67+ cells within the CD4+ T cell subset (see below). These new data are included in new **Fig. 5G**.

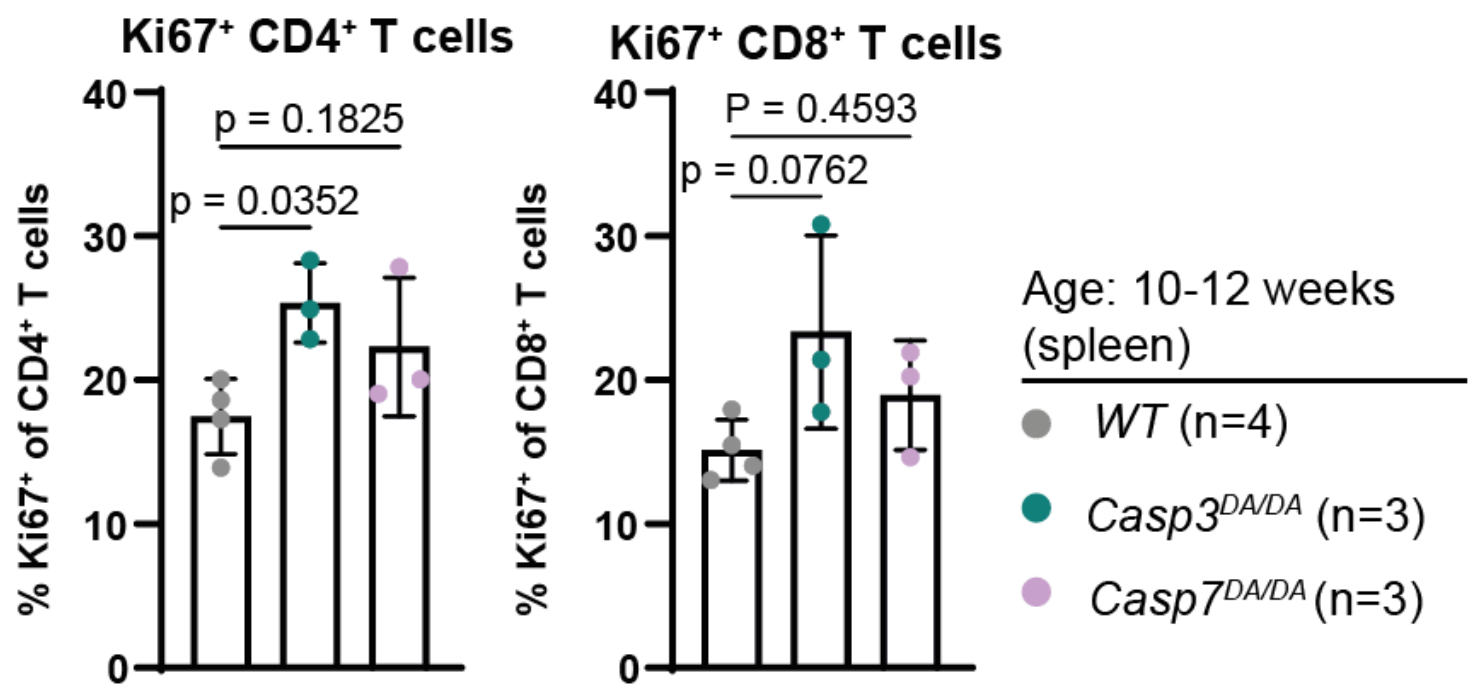

We also analysed TNF, IL2 and IFNγ expression of CD4^{+} and CD8^{+} T cells after anti-CD3/CD28 stimulation and IL6 expression in B cells stimulated with ODN1826, as anti-IgM stimulation did not lead to expression of cytokines. Cells were stimulated for 20 h, with Brefeldin A added in

the last 4 h. We could not detect any differences in cytokine production of B and T cells between the genotypes (see below exemplary plots for IFNγ and IL6). The cells were also stained for the activation markers CD69, CD25, CD44, and for B cells additionally CD71. Cells of all genotypes were nearly 100% positive for the analysed activation markers. Thus, no differences could be observed (data not shown).

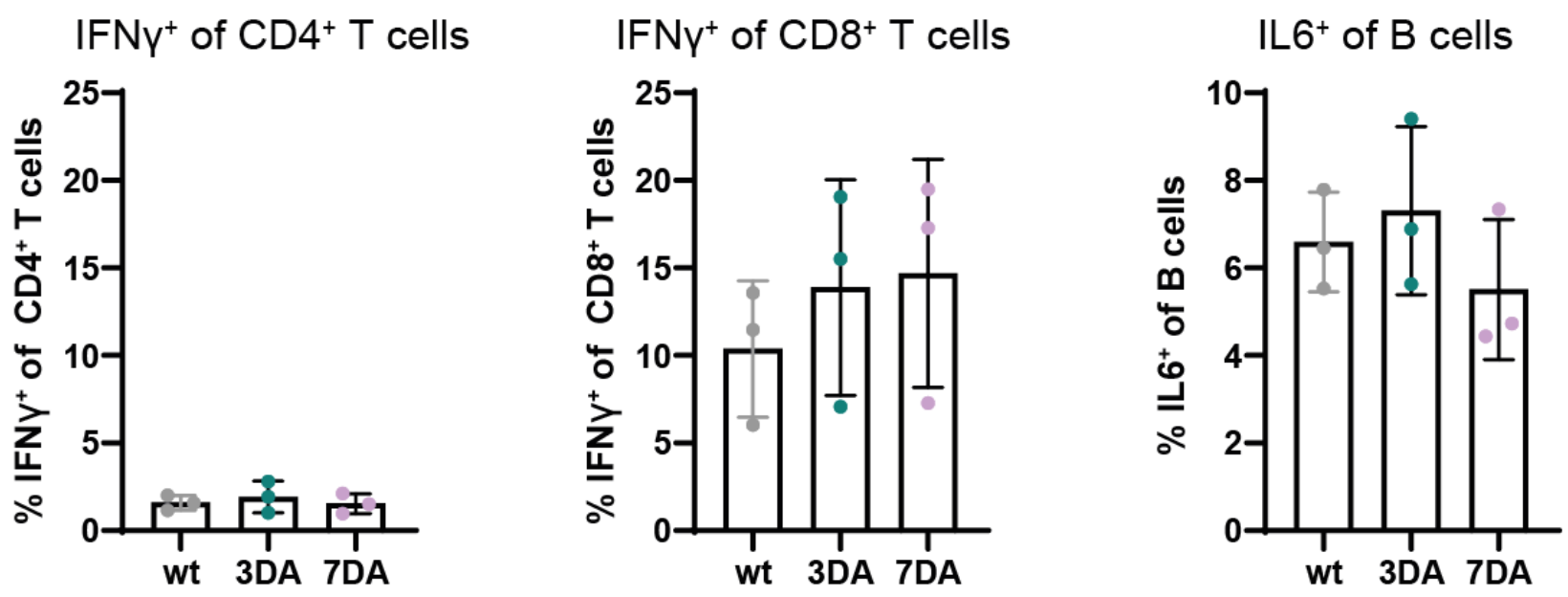

As suggested, we also performed WB analysis of T and B cells, stimulated with anti-CD3/CD28 and anti-IgM antibodies, respectively. Neither for short term (4 h) nor long term (48 h) stimulation, we could detect cGAS cleavage or phosphorylation of STING (T or B cells treated with the STING agonist DMXAA used as positive control).

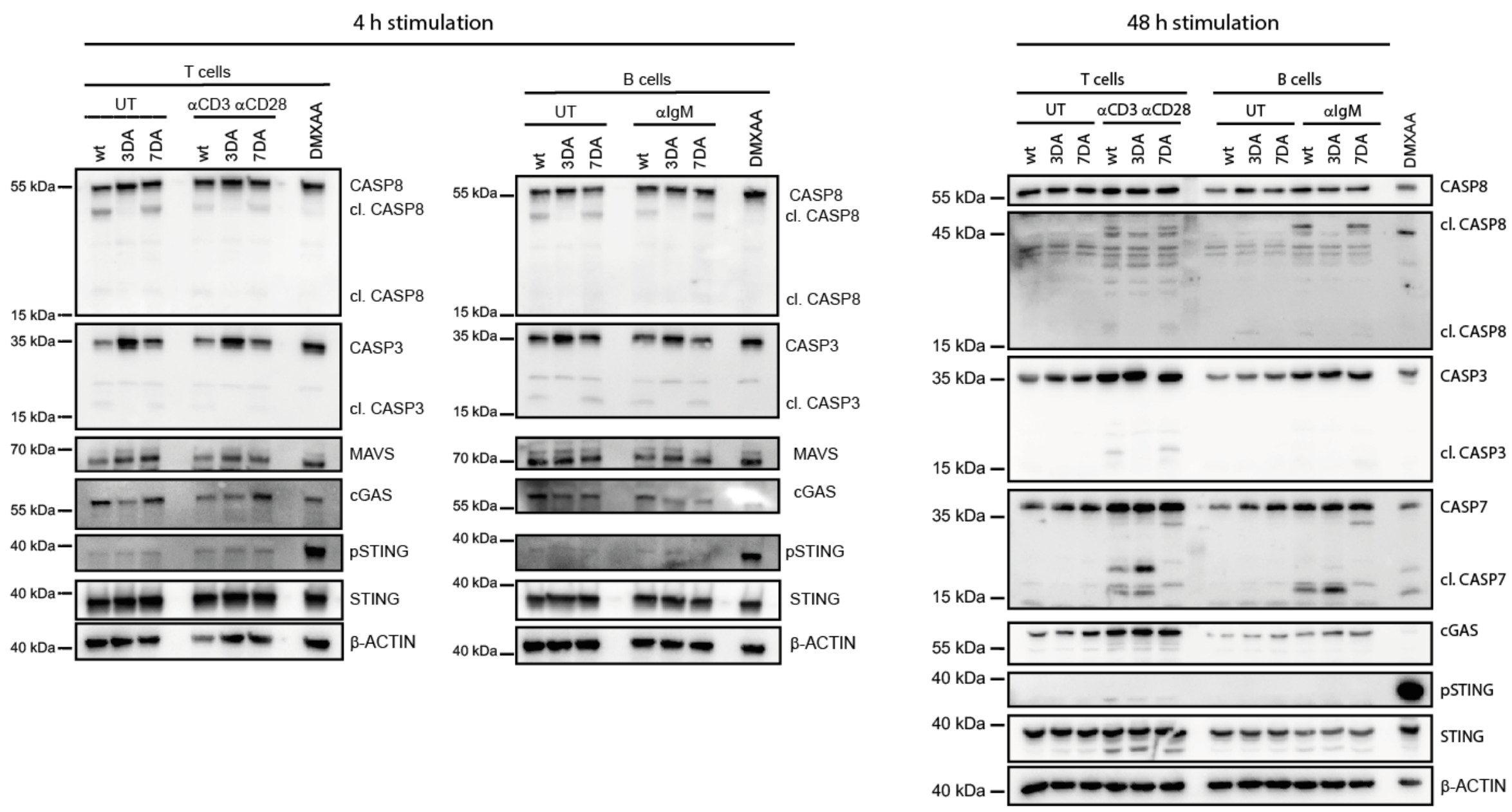

Minor comments

We also included additional information and adapted our explanations according to the recommendations in minor comments of this reviewer.

1." In vertebrates, the apoptotic caspases can be further subdivided into initiator caspases (caspases-8, -9 and -10) and executioner caspases (caspases-3, -6 and -7)." Comment: I would suggest re-wording all references to 'apoptotic caspases' to 'pro-apoptotic caspases' or 'apoptosis-associated caspases'. 'Apoptotic' refers to a mode of cell death and a caspase cannot be 'apoptotic', rather it can promote apoptosis (i.e. pro-apoptotic caspase) or inflammation (pro-inflammatory caspase).

The text is adapted, accordingly.

2. "Although apical caspases, including the initiator CASP8 and CASP9 and the inflammatory CASP1, respond to very different signals and are activated in distinct activation platforms, they all share the capability to cleave the linker peptide of executioner CASP3 (at D175) and CASP7 (at D198), which is viewed as the point-of no- return in apoptosis (Fuentes-Prior & Salvesen, 2004)."

Comment: The point of no return in the intrinsic pathway to apoptosis is generally viewed as BAX/BAK-dependent release of cytochrome c, rather than activation of caspase-3 and/or caspase-7 (or indeed caspase-9). If the latter caspases are eliminated, cells typically die via necrosis resulting from release of mitochondrial intermembrane space constituents and ATP depletion. The authors should clarify this section to avoid confusion.

The text is adapted, accordingly.

3. "Similar to extrinsic apoptosis, PARP cleavage induced by the BH3 mimetics ABT737/S-63845 was only reduced in Casp3DA/DA cells (Fig 2B). Failure of CASP3 activation was also associated with reduced CASP9 processing and lack of CASP8 processing in response to ABT737/S-63845."

Comment: This indicates a feedback loop for activation of caspase-9 via caspase-3. This has been demonstrated previously and should be cited here.

We cited Slee et al, 1999.

4. "Similar to initiator CASP8 and CASP9, CASP1 has also been shown to process CASP3 and CASP7 during the course of macrophage-mediated anti-bacterial immunity (de Vasconcelos et al, 2020; Lamkanfi et al., 2008). In order to examine the role of executioner caspase processing in pyroptosis, we isolated bone marrowderived macrophages (BMDMs) from mice and exposed them to LPS/nigericin which activates the NLRP3 inflammasome subsequently inducing pyroptosis (Fritsch et al., 2019; Simpson et al, 2022). These results indicated that the proteolytic activation of executioner caspases is unlikely decisive for the execution of pyroptosis (Figs 2E and F)."

Comment: The authors should describe the observations here more explicitly.

The text is adapted, accordingly.

5. Discussion: "CASP3 and CASP7 represent the two major executioner caspases that upon activation induce apoptotic cell death. They are closely related and are considered to have a virtually indistinguishable substrate specificity during apoptosis (Stennicke et al, 2000)"

Comment: This statement is not quite correct. Studies have shown that caspases-3 and -7 do exhibit distinct activity towards a range of specific substrates and that caspase-3 does much of the global proteolysis during apoptosis (Walsh et al., 2008 PNAS).

The text is adapted, accordingly. Walsh et al was cited in the respective section.

6. "CASP9 deficiency in mice leads to perinatal lethality which is associated with defects in neuronal tube closure, presented already at E10.5 and progresses to an expansion of the midbrain region underneath the fetal skin tissue at E13.5 (Kuida et al, 1998). None of these alterations could be observed in Casp3DA/DA/Casp7DA/DA embryos at E13.5 (Fig 1G), yet they died between E13.5 and before weaning (Figs EV1E and F). These observations are in line with a previous report analyzing Casp3-/-/Casp7-/- double-knockout mice (DKO) (Lakhani et al, 2006). Casp3-/-/Casp7-/- mice were present at normal Mendelian numbers at E20, but died rapidly after birth suggesting that unlike CASP9, neither CASP3 nor CASP7 is important for neuronal development. Independently, our data indicated that CASP3 and CASP7 provide redundant functions during embryonic development, likely by executing apoptosis."

Comment: The authors should discuss the implications of the differences observed between CASP9 deficiency in embryos and CASP3/CASP7 DKO embryos as well as Casp3DA/DA/Casp7DA/DA embryos. What are the implications of these differences? That caspase-9 cleaves additional substrates other than caspase-3 and caspase-7?

This issue is also discussed in the revised manuscript.

7.The authors repeatedly use the term 'sperms'. I think that 'sperm' is the plural.

The text is adapted, accordingly.

Dear Hamid,

Thank you for the submission of your revised manuscript (EMBOJ-2025-122134R) to The EMBO Journal and your patience during re-review. Your revision has now been seen by the four original referees, who had previously reviewed the first version of the manuscript, and we have received their comments, which are appended below.

As you will see, all four referees are very satisfied with the depth and quality of the revision, and they find the new version of the manuscript greatly improved and their initially raised criticisms and concerns satisfactorily addressed. They now all recommend publication of the manuscript without any further comments. In light of this input, I am happy to inform you that your manuscript has been accepted in principle for publication in The EMBO Journal. Congratulations on a very successful revision and thanks for comprehensively addressing the initially raised concerns of the referees.

Before we can move forward with the next steps towards publication, there are a few changes and corrections we need you to make in a final version of the manuscript:

- All funding information included in the Acknowledgements section of the manuscript should also be entered in our online manuscript handling system. The following information is currently missing from the online system: the Else Kröner-Fresenius-Stiftung (EKFS) and the Cologne Clinician Scientist Programm (CCSP); Cancer Research Centre Cologne Essen (CCCE); Deutsche Forschungsgemeinschaft (DFG, German Research Foundation), SFB1218 (269925409), SFB1607 (Project No. 501530074), Die Deutsche Krebshilfe (project number 70114685 and 70112113), and Netzwerke 2021", an initiative of the Ministry of Culture and Science of the State of North Rhine-Westphalia for the CANTAR project.

- Thank you for depositing your RNA sequencing data to a public repository. We kindly request that the mass spectrometry data reported in this manuscript be also deposited to an appropriate publicly accessible repository, and its accession ID and specific URL be provided in the Data Availability section of the revised manuscript. Please also make sure that all data will be publicly available at the time of publication. Confidential access information for the reviewers can now be removed from the Data Availability statement. Please also make sure that only one such statement is included in the revised manuscript, no separate "Materials availability" section is needed.

- Please change heading "Declaration of interests" to "Disclosure and competing interests statement".

- Heading "Summary" should be renamed to "Abstract".

- Heading "Materials and methods" should be changed to "Methods".

- The Author Contributions statement should be removed from the manuscript file. Instead, we use CRediT to specify the contributions of each author in the journal submission system. Please feel free to use the free text box to provide more detailed descriptions during submission. See also our guide to authors for more information: https://link.springer.com/partners/embo-press/editorial-policies#Authorship.

- We noticed that callouts for Table EV9 are missing in the manuscript.

- Source file names, titles, legends, and manuscript callouts all need to be updated to "Dataset EV1-EV6" instead of Table EV1-EV2, EV4-EV5, and EV8-EV9. Each Dataset should be uploaded as an individual Excel file (not zipped), with its legend included in a separate tab/sheet in the same file.

- Tables EV3, EV6-EV7 should be uploaded as individual Excel files (not zipped) and renamed to "Table EV1-EV3"; please make sure to also rename the corresponding callouts accordingly as well. The legend of each Table should remain above the table in each Excel file.

- The title page of the Appendix PDF file should contain heading "Appendix for", followed by the manuscript's title and the Table of Contents (author and affiliation list are not necessary).

- Please note that EMBO press papers are accompanied online by:
A) a short (2 sentences) summary of the findings and their significance,
B) 2-5 short bullet points highlighting the key results, and
C) a synopsis image in .jpg or .png format that is exactly 550 pixels wide x 300 pixels high. Please note that all text needs to be legible at this size.
Please upload this information along with your revised manuscript (the text for A and B should be provided in a separate Word file).

- Our data editors have checked your Figures and their legends, and raised the following queries. Please address all points

below completely in your revised manuscript (all changes should be highlighted or "tracked"):
1. Please provide the exact p-values in the legends of Figures 5B, EV2D, EV4 A.
2. Please indicate the statistical test used for data analysis in the legend of Figure EV4 F.
3. Please note that the error bars must be defined in the legend of Figure EV5 E.
4. Please note that scale bar and its definition are missing in the legend of Figure EV4 C.

- The manuscript sections need to be named and ordered as follows: Title page - Abstract - Introduction - Results - Discussion - Methods - Data Availability - Acknowledgements - Disclosure and Competing Interests Statement - References - Figure Legends - main Tables (if there are any) - Expanded View Figure Legends.

- Please also note that as part of the EMBO Press transparent editorial process, The EMBO Journal publishes online a Peer Review File along with each accepted manuscript. This File will be published in conjunction with your paper and will include the referee reports, your point-by-point responses and all pertinent correspondence relating to the manuscript. Your Author's Checklist will also be published at the end of the Peer Review File. Please let us know in case you want to remove any data or figures from your point-by-point responses before they are published as part of the Peer Review File. Retaining unpublished data in the Peer Review File means that these count as published and that the Peer Review File would need to be referenced in future publications. Please let the editorial office know in case you want to remove any data from this file (contact@embojournal.org).

We look forward to seeing a final version of your manuscript as soon as possible. Please let us know if you have any questions and use this link to submit your revision: https://emboj.msubmit.net/cgi-bin/main.plex.

Best regards,

Ioannis

Ioannis Papaioannou, PhD
Editor, The EMBO Journal
i.papaioannou@embojournal.org

Referee #1:

The authors have satisfactorily responded to my comments. The quality of the MS has greatly improved and hence the MS can now be accepted for publication by EMBO J.

Referee #2:

The authors have addressed all my comments satisfatorily and I therefore support publication of this manuscript.

Referee #3:

The authors have adequately addressed my concerns. I recommend publication

Referee #4:

This is an interesting study that identifies distinct requirements for caspase-3 and caspase-7 in vivo for the suppression of Type I IFN signaling in the lymphoid compartment and spermatogenesis, respectively. Processing-defective Casp7DA mice exhibited spermatogenesis defects, whereas Casp3DA mice exhibited splenomegaly and perturbation of the ratios of CD4/CD8 T-cells and other lymphoid abnormalities, potentially related to imbalanced Type I IFN signaling.

The authors have more than satisfactorily addressed all of my previous comments and are to be congratulated on an impressive and very interesting study.

All editorial and formatting issues were resolved by the authors.

2nd Revision - Editorial Decision 19th Jun 2026

Dear Hamid,

Congratulations on an excellent manuscript! I am very pleased to inform you that it has now been accepted for publication in The EMBO Journal. Many thanks for comprehensively addressing the initially raised referees' concerns and our editorial requests for changes and corrections.

Your manuscript will be processed for publication by EMBO Press. It will be copy edited and you will receive page proofs prior to publication. Please note that you will be contacted by Springer Nature Author Services to complete licensing and payment information.

You may qualify for financial assistance for your publication charges - either via a Springer Nature fully open access agreement or an EMBO initiative. Check your eligibility: https://link.springer.com/journal/44318/how-to-publish-with-us

Should you be planning a Press Release on your article, please notify the production team via this form in order to coordinate publication and release dates: https://forms.cloud.microsoft/e/eH4A8JVP6g

If you have any questions, please do not hesitate to contact the Editorial Office. Thank you for your contribution to The EMBO Journal. Working with you has been a pleasure.

Best regards,

Ioannis

Ioannis Papaioannou, PhD
Editor, The EMBO Journal
i.papaioannou@embojournal.org

*****
Please note that it is The EMBO Journal policy for the transcript of the editorial process (containing referee reports and your response letters) to be published as an online supplement to each paper. If you should prefer removal of any referee-only figures included in the point-by-point response(s), e.g. because they may still be used for future publication or because they have been reproduced from published work by others, please do let us know immediately via response email.
More information is available here: https://link.springer.com/partners/embo-press/editorial-policies#Peer%20review

** Click here to be directed to your login page: https://emboj.msubmit.net

EMBO Press Author Checklist

Corresponding Author Name: Hamid Kashkar
Journal Submitted to: The EMBO Journal
Manuscript Number: 2025-122134

USEFUL LINKS FOR COMPLETING THIS FORM
[The EMBO Journal - Author Guidelines]
[EMBO Reports - Author Guidelines]
[Molecular Systems Biology - Author Guidelines]
[EMBO Molecular Medicine - Author Guidelines]

Reporting Checklist for Life Science Articles (updated January
This checklist is adapted from Materials Design Analysis Reporting (MDAR) Checklist for Authors. MDAR establishes a minimum set of requirements in transparent reporting in the life sciences (see Statement of Task: 10.31222/osf.io/9sm4x). Please follow the journal's guidelines in preparing your
Please note that a copy of this checklist will be published alongside your article.

Abridged guidelines for figures
1. Data
The data shown in figures should satisfy the following conditions:
- the data were obtained and processed according to the field's best practice and are presented to reflect the results of the experiments in an accurate and unbiased manner.
- ideally, figure panels should include only measurements that are directly comparable to each other and obtained with the same assay.
- plots include clearly labeled error bars for independent experiments and sample sizes. Unless justified, error bars should not be shown for technical
- if n<5, the individual data points from each experiment should be plotted. Any statistical test employed should be justified.
- Source Data should be included to report the data underlying figures according to the guidelines set out in the authorship guidelines on Data

2. Captions
Each figure caption should contain the following information, for each panel where they are relevant:
- a specification of the experimental system investigated (eg cell line, species name).
- the assay(s) and method(s) used to carry out the reported observations and measurements.
- an explicit mention of the biological and chemical entity(ies) that are being measured.
- an explicit mention of the biological and chemical entity(ies) that are altered/varied/perturbed in a controlled manner.
- the exact sample size (n) for each experimental group/condition, given as a number, not a range;
- a description of the sample collection allowing the reader to understand whether the samples represent technical or biological replicates (including how many animals, litters, cultures, etc.).
- a statement of how many times the experiment shown was independently replicated in the laboratory.
- definitions of statistical methods and measures:
  - common tests, such as t-test (please specify whether paired vs. unpaired), simple χ2 tests, Wilcoxon and Mann-Whitney tests, can be unambiguously identified by name only, but more complex techniques should be described in the methods section;
  - are tests one-sided or two-sided?
  - are there adjustments for multiple comparisons?
  - exact statistical test results, e.g., P values = x but not P values < x;
  - definition of 'center values' as median or average;
  - definition of error bars as s.d. or s.e.m.

Please complete ALL of the questions below.
Select "Not Applicable" only when the requested information is not relevant for your study.

Materials

Newly Created Materials	Information included in the manuscript?	In which section is the information available? (Reagents and Tools Table, Materials and Methods, Figures, Data Availability Section)
New materials and reagents need to be available; do any restrictions apply?	Not Applicable	

Antibodies	Information included in the manuscript?	In which section is the information available? (Reagents and Tools Table, Materials and Methods, Figures, Data Availability Section)
For **antibodies** provide the following information:<br>- Commercial antibodies: RRID (if possible) or supplier name, catalogue number and or/clone number<br>- Non-commercial: RRID or citation	Yes	Reagents and Tools Table

DNA and RNA sequences	Information included in the manuscript?	In which section is the information available? (Reagents and Tools Table, Materials and Methods, Figures, Data Availability Section)
**Short novel DNA or RNA including primers, probes**: provide the sequences.	Yes	Material and Methods

Cell materials	Information included in the manuscript?	In which section is the information available? (Reagents and Tools Table, Materials and Methods, Figures, Data Availability Section)
**Cell lines:** Provide species information, strain. Provide accession number in repository **OR** supplier name, catalog number, clone number, and/**OR** RRID.	Yes	Material and Methods
**Primary cultures:** Provide species, strain, sex of origin, genetic modification status.	Yes	Material and Methods anf Figure legends
Report if the cell lines were recently **authenticated** (e.g., by STR profiling) and tested for mycoplasma contamination.	Yes	Material and Methods

Experimental animals	Information included in the manuscript?	In which section is the information available? (Reagents and Tools Table, Materials and Methods, Figures, Data Availability Section)
**Laboratory animals or Model organisms:** Provide species, strain, sex, age, genetic modification status. Provide accession number in repository **OR** supplier name, catalog number, clone number, **OR** RRID.	Yes	Materials and Methods, Figures
**Animal observed in or captured from the field:** Provide species, sex, and age where possible.	Yes	Materials and Methods, Figures
Please detail **housing and husbandry conditions**.	Yes	Materials and Methods

Plants and microbes	Information included in the manuscript?	In which section is the information available? (Reagents and Tools Table, Materials and Methods, Figures, Data Availability Section)
**Plants:** provide species and strain, ecotype and cultivar where relevant, unique accession number if available, and source (including location for collected wild specimens).	Not Applicable	
**Microbes:** provide species and strain, unique accession number if available, and source.	Not Applicable	

Human research participants	Information included in the manuscript?	In which section is the information available? (Reagents and Tools Table, Materials and Methods, Figures, Data Availability Section)
If collected and within the bounds of privacy constraints report on age, sex and gender or ethnicity for all study participants.	Not Applicable	

Core facilities	Information included in the manuscript?	In which section is the information available? (Reagents and Tools Table, Materials and Methods, Figures, Data Availability Section)
If your work benefited from core facilities, was their service mentioned in the acknowledgments section?	Yes	Acknowledgement section

Design

Study protocol	Information included in the manuscript?	In which section is the information available? (Reagents and Tools Table, Materials and Methods, Figures, Data Availability Section)
If study protocol has been **pre-registered, provide DOI in the manuscript**. For clinical trials, provide the trial registration number **OR** cite DOI.	Not Applicable	
Report the **clinical trial registration number** (at ClinicalTrials.gov or equivalent), where applicable.	Not Applicable	

Laboratory protocol	Information included in the manuscript?	In which section is the information available? (Reagents and Tools Table, Materials and Methods, Figures, Data Availability Section)
Provide DOI OR other citation details if **external detailed step-by-step protocols** are available.	Not Applicable	

Experimental study design and statistics	Information included in the manuscript?	In which section is the information available? (Reagents and Tools Table, Materials and Methods, Figures, Data Availability Section)
Include a statement about **sample size** estimate even if no statistical methods were used.	Yes	Material and Methods
Were any steps taken to minimize the effects of subjective bias when allocating animals/samples to treatment (e.g. **randomization procedure**)? If yes, have they been described?	Not Applicable	
Include a statement about **blinding** even if no blinding was done.	Yes	Material and Methods
Describe **inclusion/exclusion criteria** if samples or animals were excluded from the analysis. Were the criteria pre-established?<br><br>If sample or data points were omitted from analysis, report if this was due to attrition or intentional exclusion and provide justification.	Not Applicable	
For every figure, are **statistical tests** justified as appropriate? Do the data meet the assumptions of the tests (e.g., normal distribution)? Describe any methods used to assess it. Is there an estimate of variation within each group of data? **Is** the variance similar between the groups that are being statistically compared?	Yes	Figure Legends

Sample definition and in-laboratory replication	Information included in the manuscript?	In which section is the information available? (Reagents and Tools Table, Materials and Methods, Figures, Data Availability Section)
In the figure legends: state number of times the experiment was **replicated** in laboratory.	Yes	Figure legends
In the figure legends: define whether data describe **technical or biological replicates**.	Yes	Figure legends

Ethics

Ethics	Information included in the manuscript?	In which section is the information available? (Reagents and Tools Table, Materials and Methods, Figures, Data Availability Section)
Studies involving **human participants**: State details of **authority granting ethics approval** (IRB or equivalent committee(s), provide reference number for approval.	Not Applicable	
Studies involving **human participants**: Include a statement confirming that **informed consent** was obtained from all subjects and that the experiments conformed to the principles set out in the WMA Declaration of Helsinki and the Department of Health and Human Services Belmont Report.	Not Applicable	
Studies involving **human participants:** For publication of **patient photos**, include a statement confirming that consent to publish was obtained.	Not Applicable	
Studies involving experimental **animals**: State details of **authority granting ethics approval** (IRB or equivalent committee(s), provide reference number for approval. Include a statement of compliance with ethical regulations.	Yes	Material and Methods
Studies involving **specimen and field samples:** State if relevant **permits** obtained, provide details of authority approving study; if none were required, explain why.	Not Applicable	

Dual Use Research of Concern (DURC)	Information included in the manuscript?	In which section is the information available? (Reagents and Tools Table, Materials and Methods, Figures, Data Availability Section)
Could your study fall under dual use research restrictions? Please check biosecurity documents and list of **select agents and toxins** (CDC): https://www.selectagents.gov/sat/list.htm	Not Applicable	
If you used a select agent, is the security level of the lab appropriate and reported in the manuscript?	Not Applicable	
If a study is subject to dual use research of concern regulations, is the name of the **authority granting approval and reference number** for the regulatory approval provided in the manuscript?	Not Applicable	

Reporting

The MDAR framework recommends adoption of discipline-specific guidelines, established and endorsed through community initiatives. Journals have their own policy about requiring specific guidelines and recommendations to complement MDAR.

Adherence to community standards	Information included in the manuscript?	In which section is the information available? (Reagents and Tools Table, Materials and Methods, Figures, Data Availability Section)
State if relevant guidelines or checklists (e.g., **ICMJE, MIBBI, ARRIVE, PRISMA**) have been followed or provided.	Not Applicable	
For **tumor marker prognostic studies,** we recommend that you follow the **REMARK** reporting guidelines (see link list at top right). See author guidelines, under 'Reporting Guidelines'. Please confirm you have followed these guidelines.	Not Applicable	
For **phase II and III randomized controlled trials**, please refer to the **CONSORT** flow diagram (see link list at top right) and submit the CONSORT checklist (see link list at top right) with your submission. See author guidelines, under 'Reporting Guidelines'. Please confirm you have submitted this list.	Not Applicable	

Data Availability

Data availability	Information included in the manuscript?	In which section is the information available? (Reagents and Tools Table, Materials and Methods, Figures, Data Availability Section)
Have **primary datasets** been deposited according to the journal's guidelines (see 'Data Deposition' section) and the respective accession numbers provided in the Data Availability Section?	Yes	Data availability section
Were **human clinical and genomic datasets** deposited in a public access-controlled repository in accordance to ethical obligations to the patients and to the applicable consent agreement?	Not Applicable	
Are **computational models** that are central and integral to a study available without restrictions in a machine-readable form? Were the relevant accession numbers or links provided?	Not Applicable	
If publicly available data were reused, provide the respective **data citations in the reference list**.	Not Applicable	
