# Supplementary material for: Proteolytic activation of executioner caspase-3 and -7 regulates different physiological processes in mice
Source: EMBO J. 2026 Jul 21;45(17):6197–228. doi: 10.1038/s44318-026-00871-4 (PMC13534459; doi:10.1038/s44318-026-00871-4)
Supplement: Supplementary file 6 [file 44318_2026_871_MOESM14_ESM.zip › Source data Figure 3/3A/Labeling WBs.pdf]

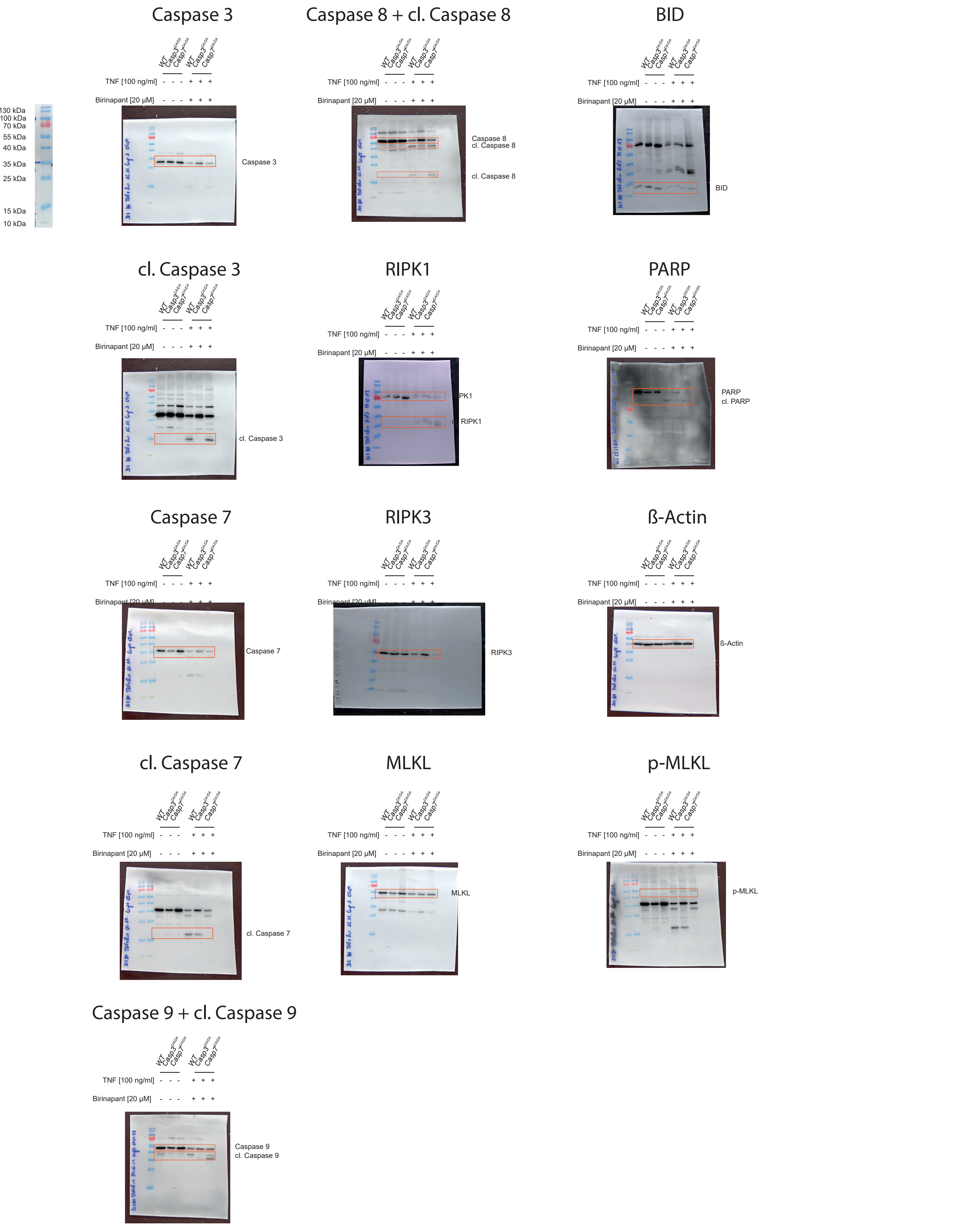
Caspase 3
Caspase 8 + cl. Caspase 8
BID
WT
Casp3DA/DA
Casp7DA/DA
TNF [100 ng/ml] - - - + + +
Birinapant [20 µM] - - - + + +
130 kDa
100 kDa
70 kDa
55 kDa
40 kDa
35 kDa
25 kDa
15 kDa
10 kDa
Caspase 3
Caspase 8
cl. Caspase 8
cl. Caspase 8
BID
cl. Caspase 3
RIPK1
PARP
cl. Caspase 3
RIPK1
PARP
cl. PARP
Caspase 7
RIPK3
ß-Actin
Caspase 7
RIPK3
ß-Actin
cl. Caspase 7
MLKL
p-MLKL
cl. Caspase 7
MLKL
p-MLKL
Caspase 9 + cl. Caspase 9
Caspase 9
cl. Caspase 9
