# Supplementary material for: Proteolytic activation of executioner caspase-3 and -7 regulates different physiological processes in mice
Source: EMBO J. 2026 Jul 21;45(17):6197–228. doi: 10.1038/s44318-026-00871-4 (PMC13534459; doi:10.1038/s44318-026-00871-4)
Supplement: Supplementary file 6 [file 44318_2026_871_MOESM14_ESM.zip › Source data Figure 3/3B/Labeling WBs.pdf]

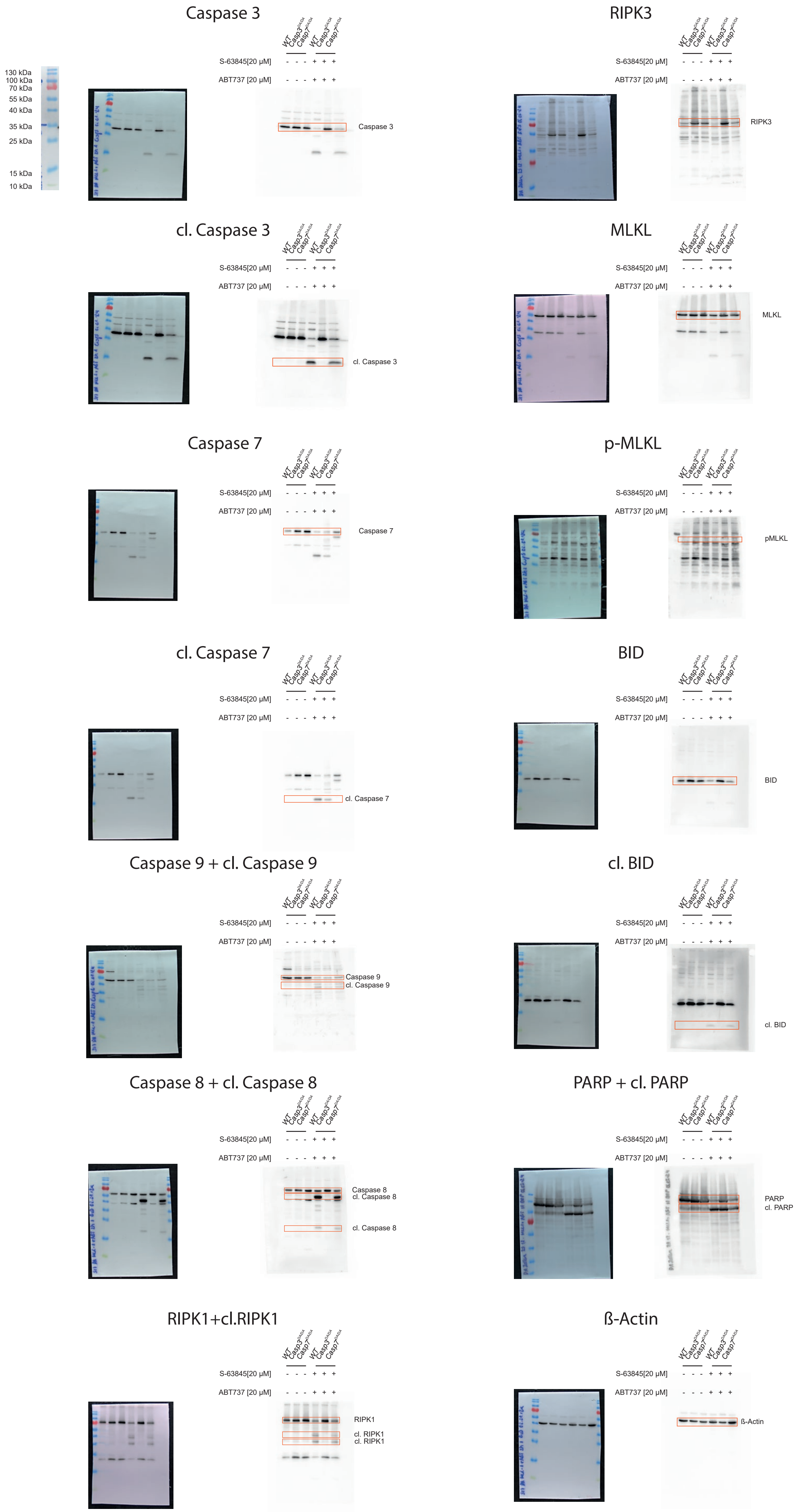
130 kDa
100 kDa
70 kDa
55 kDa
40 kDa
35 kDa
25 kDa
15 kDa
10 kDa
Caspase 3
cl. Caspase 3
Caspase 7
cl. Caspase 7
Caspase 9 + cl. Caspase 9
Caspase 9
cl. Caspase 9
Caspase 8 + cl. Caspase 8
Caspase 8
cl. Caspase 8
RIPK1+cl.RIPK1
RIPK1
cl. RIPK1
RIPK3
MLKL
p-MLKL
pMLKL
BID
cl. BID
PARP + cl. PARP
PARP
cl. PARP
ß-Actin
WT
Casp3
Casp7
S-63845[20 µM] - - - + + +
ABT737 [20 µM] - - - + + +
