# Supplementary material for: Proteolytic activation of executioner caspase-3 and -7 regulates different physiological processes in mice
Source: EMBO J. 2026 Jul 21;45(17):6197–228. doi: 10.1038/s44318-026-00871-4 (PMC13534459; doi:10.1038/s44318-026-00871-4)

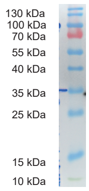

Caspase 3 + cl. Caspase 3

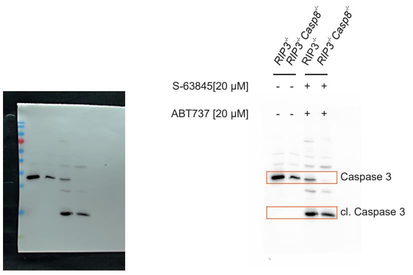

MLKL

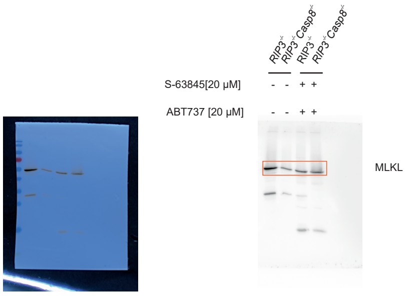

Caspase 7 + cl. Caspase 7

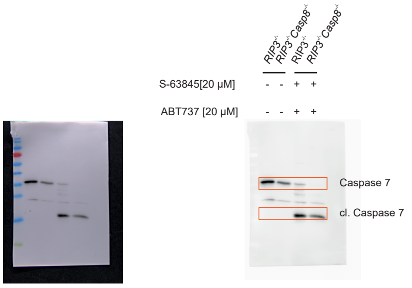

β-Actin

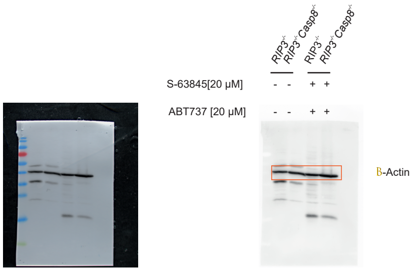

Caspase 8 + cl. Caspase 8

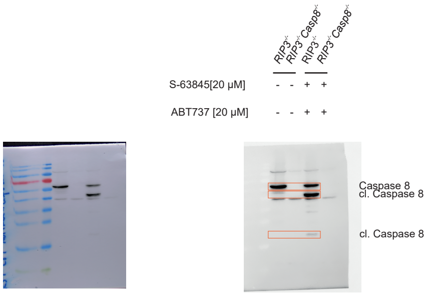

BID + cl. BID

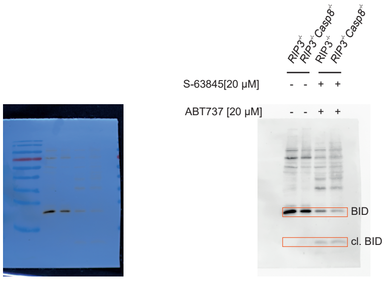

Caspase 9 + cl. Caspase 9

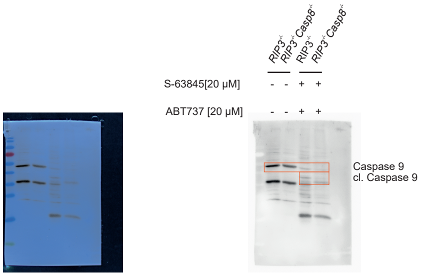

RIPK1 + cl. RIPK1

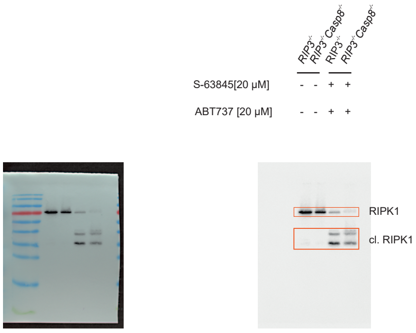

Supplement: Supplementary file 6 [file 44318_2026_871_MOESM14_ESM.zip › Source data Figure 3/3C/Labeling WBs.pdf]
