# Supplementary material for: Proteolytic activation of executioner caspase-3 and -7 regulates different physiological processes in mice
Source: EMBO J. 2026 Jul 21;45(17):6197–228. doi: 10.1038/s44318-026-00871-4 (PMC13534459; doi:10.1038/s44318-026-00871-4)
Supplement: Supplementary file 6 [file 44318_2026_871_MOESM14_ESM.zip › Source data Figure 3/3E/Labeling WBs.pdf]

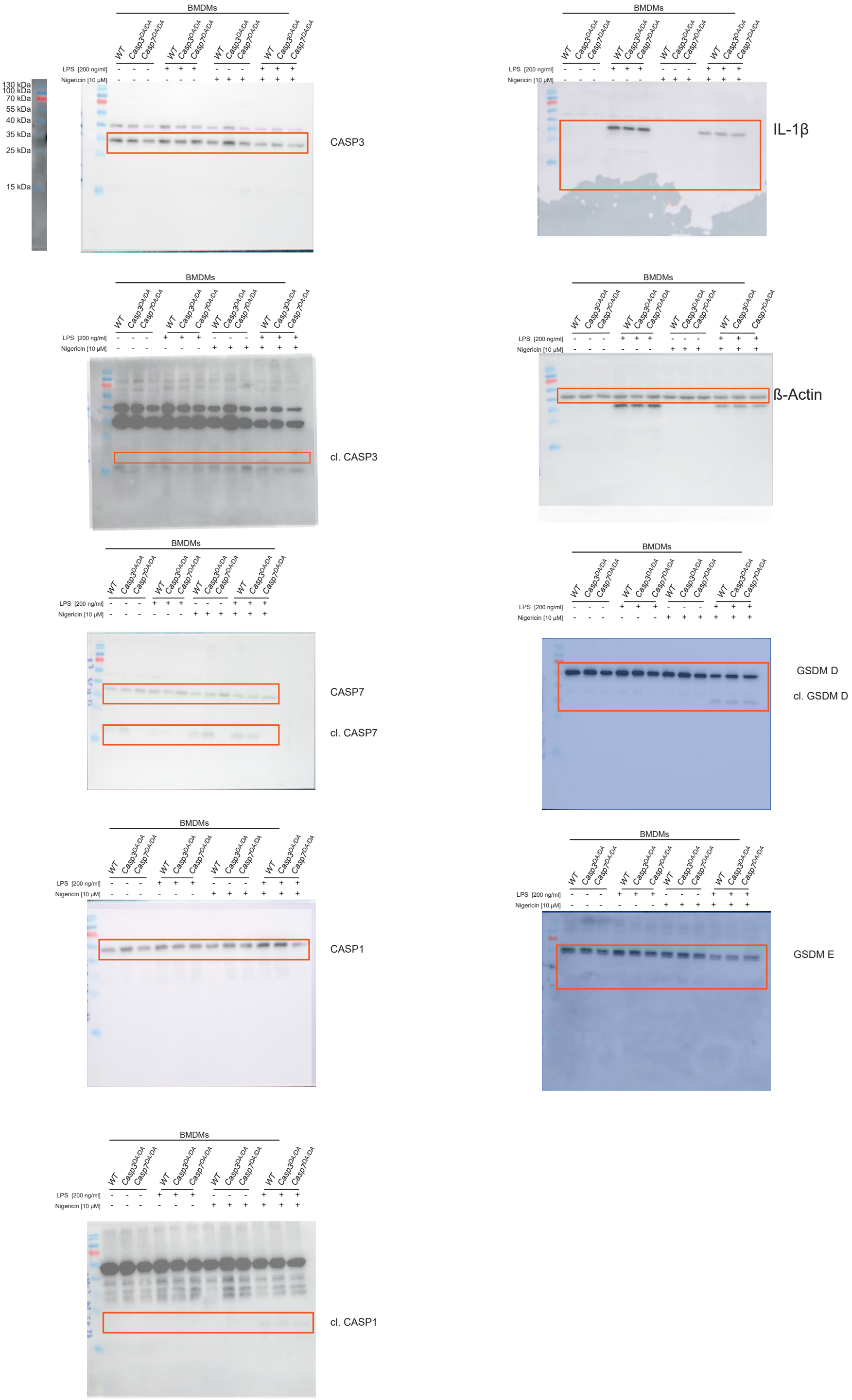
BMDMs
WT
Casp3DA/DA
Casp7DA/DA
LPS [200 ng/ml]
Nigericin [10 µM]
130 kDa
100 kDa
70 kDa
55 kDa
40 kDa
35 kDa
25 kDa
15 kDa
CASP3
IL-1β
cl. CASP3
ß-Actin
CASP7
cl. CASP7
GSDM D
cl. GSDM D
CASP1
GSDM E
cl. CASP1
