# Supplementary material for: Proteolytic activation of executioner caspase-3 and -7 regulates different physiological processes in mice
Source: EMBO J. 2026 Jul 21;45(17):6197–228. doi: 10.1038/s44318-026-00871-4 (PMC13534459; doi:10.1038/s44318-026-00871-4)

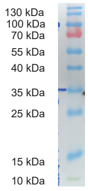

Caspase 3

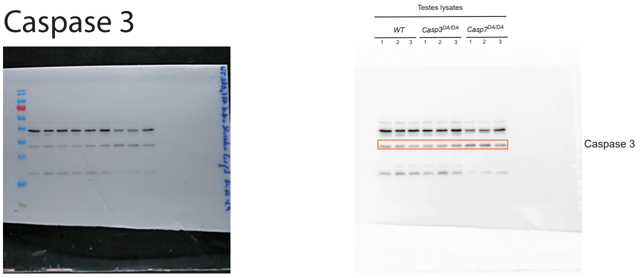

cl. Caspase 3 (unspecific)

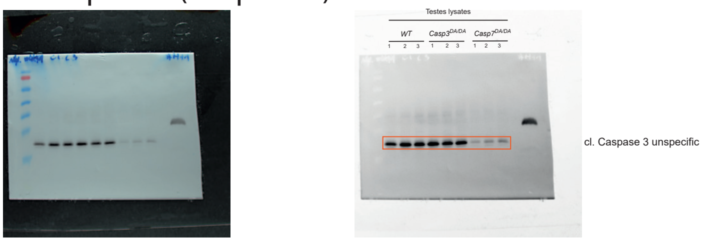

Caspase 7

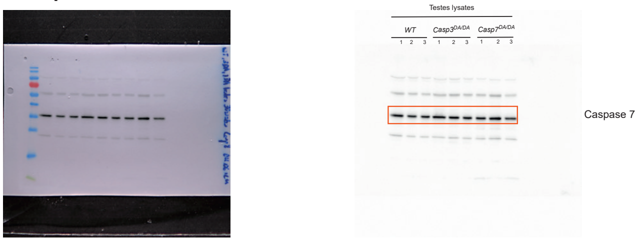

Caspase 8

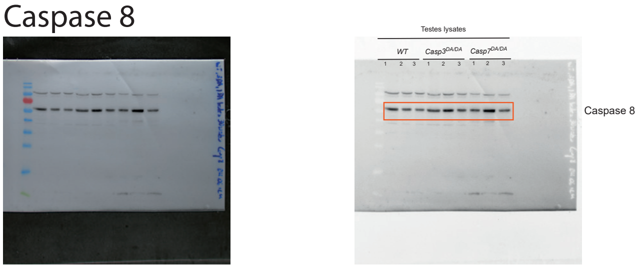

Caspase 9

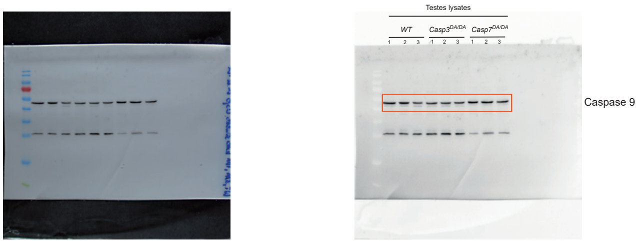

BAK

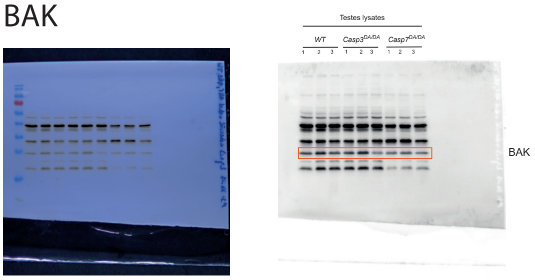

ASM + cl. ASM

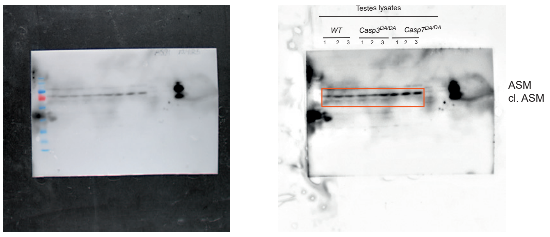

ß-Actin

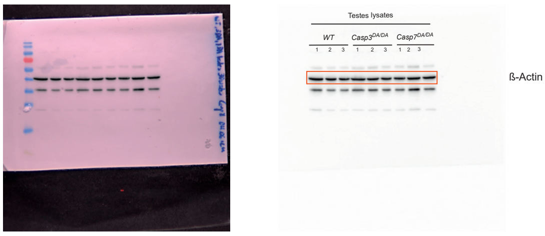

BAX

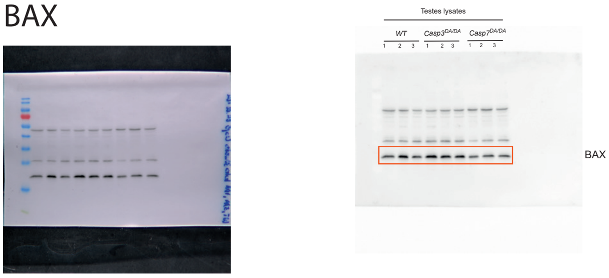

Supplement: Supplementary file 7 [file 44318_2026_871_MOESM15_ESM.zip › Source data Figure 4/4F/Labeling WBs.pdf]
