# Supplementary material for: Proteolytic activation of executioner caspase-3 and -7 regulates different physiological processes in mice
Source: EMBO J. 2026 Jul 21;45(17):6197–228. doi: 10.1038/s44318-026-00871-4 (PMC13534459; doi:10.1038/s44318-026-00871-4)
Supplement: Supplementary file 7 [file 44318_2026_871_MOESM15_ESM.zip › Source data Figure 4/4G/Labeling WBs.pdf]

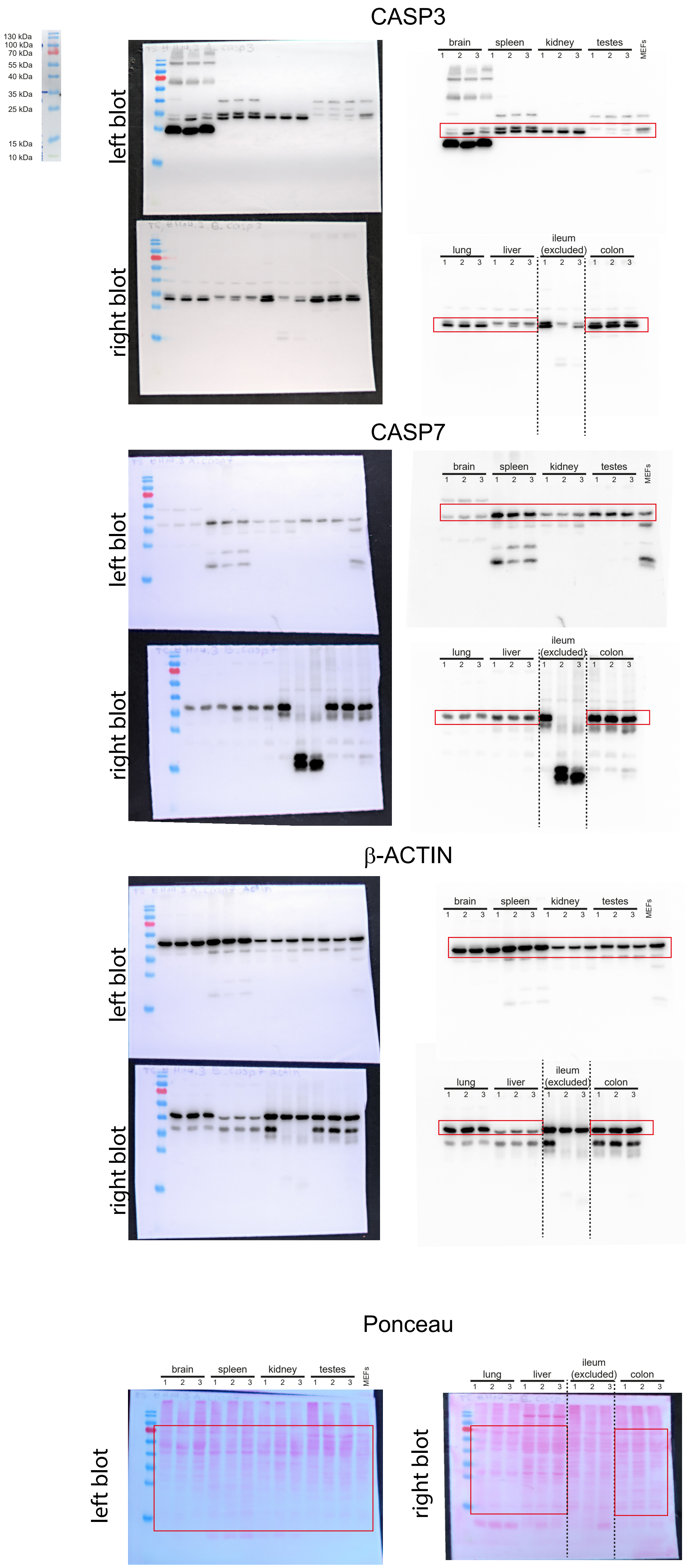
CASP3
130 kDa
100 kDa
70 kDa
55 kDa
40 kDa
35 kDa
25 kDa
15 kDa
10 kDa
left blot
right blot
brain
spleen
kidney
testes
MEFs
1 2 3
lung
liver
ileum (excluded)
colon
CASP7
β-ACTIN
Ponceau
