# Supplementary material for: Proteolytic activation of executioner caspase-3 and -7 regulates different physiological processes in mice
Source: EMBO J. 2026 Jul 21;45(17):6197–228. doi: 10.1038/s44318-026-00871-4 (PMC13534459; doi:10.1038/s44318-026-00871-4)
Supplement: Supplementary file 9 [file 44318_2026_871_MOESM17_ESM.zip › Source data Figure 6/6C/Labeling WBs.pdf]

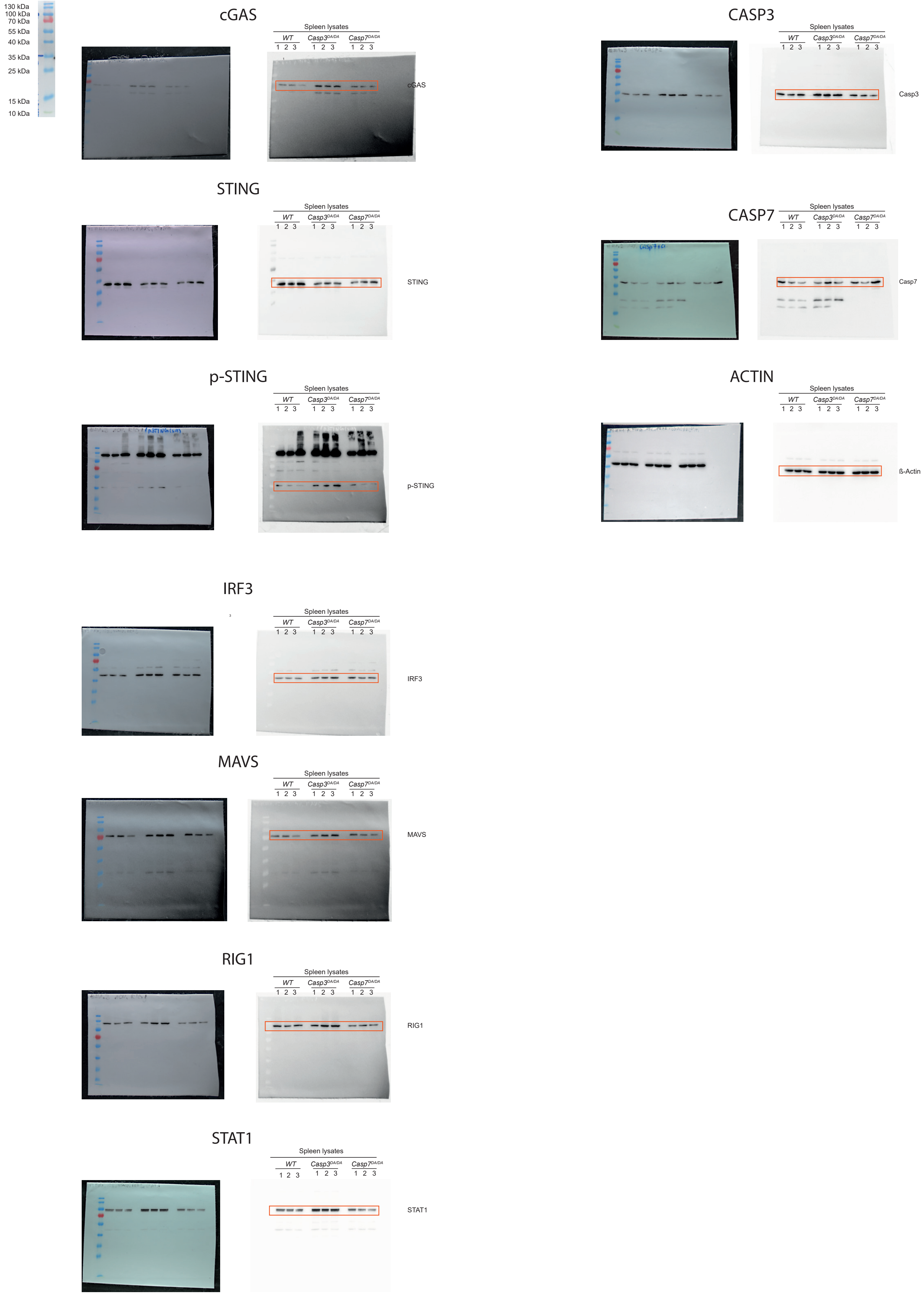
130 kDa
100 kDa
70 kDa
55 kDa
40 kDa
35 kDa
25 kDa
15 kDa
10 kDa
cGAS
Spleen lysates
WT
Casp3DA/DA
Casp7DA/DA
1 2 3
cGAS
STING
STING
p-STING
p-STING
IRF3
IRF3
MAVS
MAVS
RIG1
RIG1
STAT1
STAT1
CASP3
Casp3
CASP7
Casp7
ACTIN
ß-Actin
