# Supplementary material for: Proteolytic activation of executioner caspase-3 and -7 regulates different physiological processes in mice
Source: EMBO J. 2026 Jul 21;45(17):6197–228. doi: 10.1038/s44318-026-00871-4 (PMC13534459; doi:10.1038/s44318-026-00871-4)

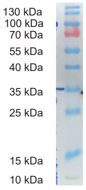

Caspase 3 + cl. Caspase 3

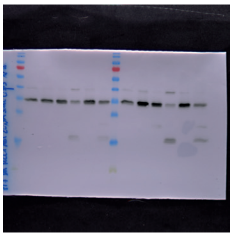

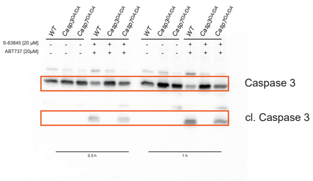

Caspase 7 + cl. Caspase 7

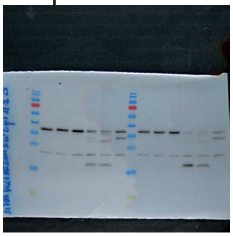

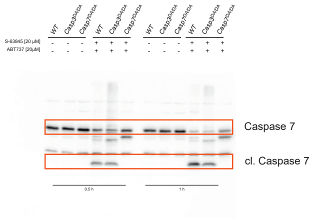

MAVS + cl. MAVS

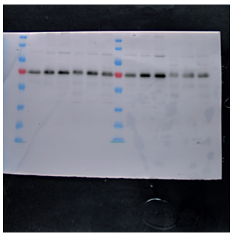

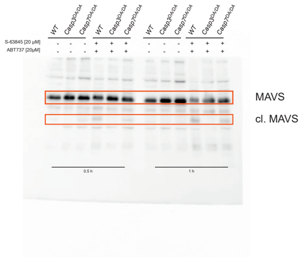

cGAS + cl. cGAS

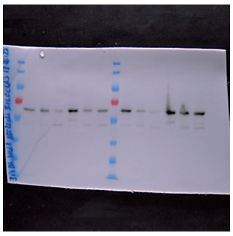

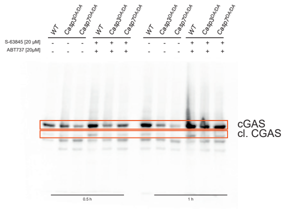

IRF3

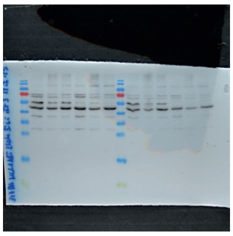

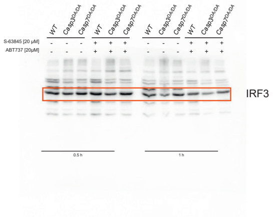

ß-Actin

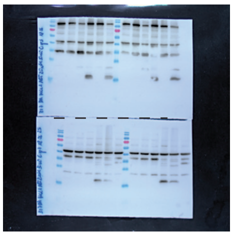

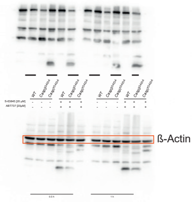

Supplement: Supplementary file 9 [file 44318_2026_871_MOESM17_ESM.zip › Source data Figure 6/6D/Labeling WBs.pdf]
