# Supplementary material for: Proteolytic activation of executioner caspase-3 and -7 regulates different physiological processes in mice
Source: EMBO J. 2026 Jul 21;45(17):6197–228. doi: 10.1038/s44318-026-00871-4 (PMC13534459; doi:10.1038/s44318-026-00871-4)

Extended Figure 3E

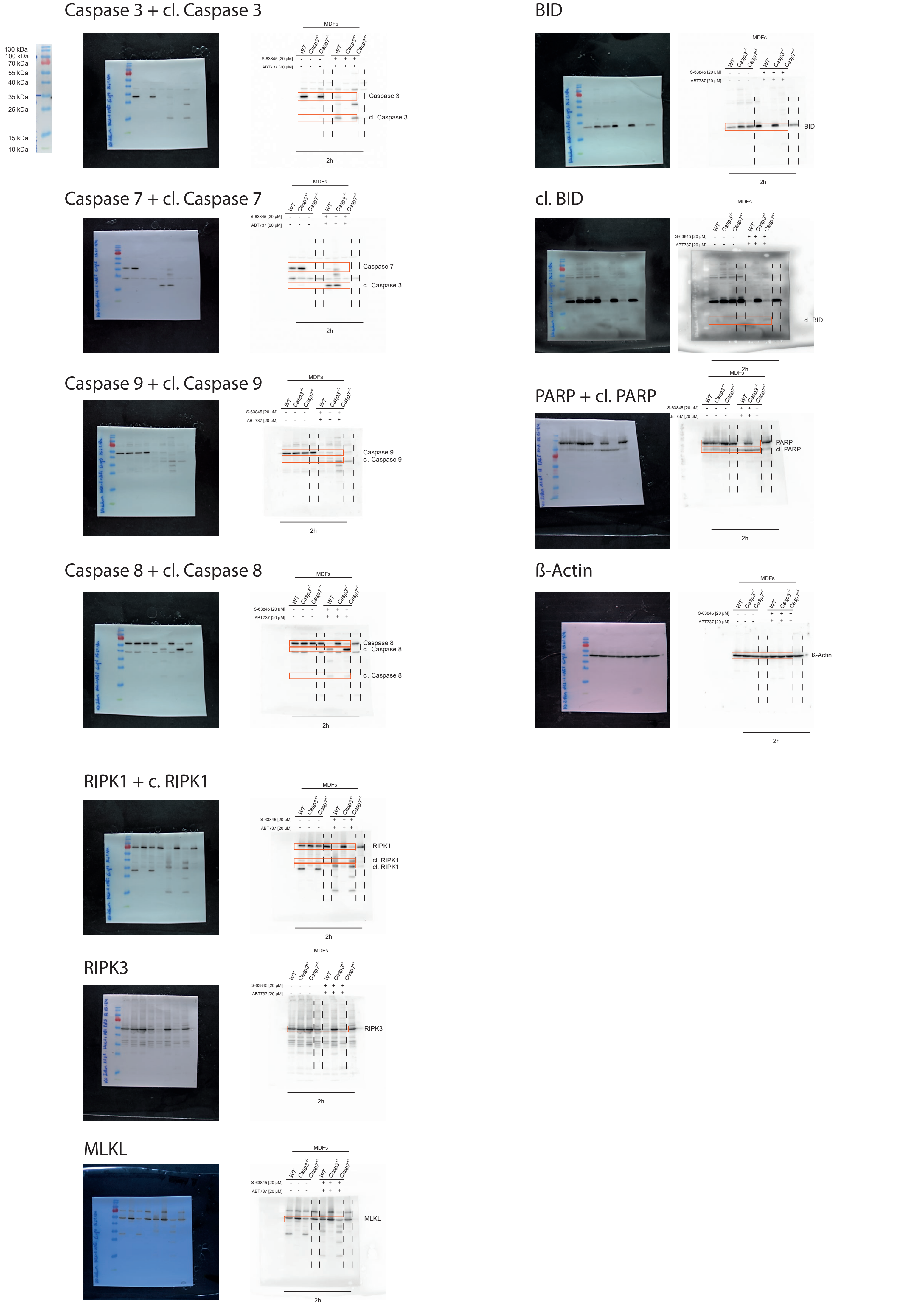

Supplement: Supplementary file 10 [file 44318_2026_871_MOESM18_ESM.zip › WB source data for EV figures/Figure EV3E source data WBs/Labeling WBs.pdf]
