# Supplementary material for: Proteolytic activation of executioner caspase-3 and -7 regulates different physiological processes in mice
Source: EMBO J. 2026 Jul 21;45(17):6197–228. doi: 10.1038/s44318-026-00871-4 (PMC13534459; doi:10.1038/s44318-026-00871-4)
Supplement: Supplementary file 10 [file 44318_2026_871_MOESM18_ESM.zip › WB source data for EV figures/Figure EV3G source data WBs/Labeling WBs.pdf]

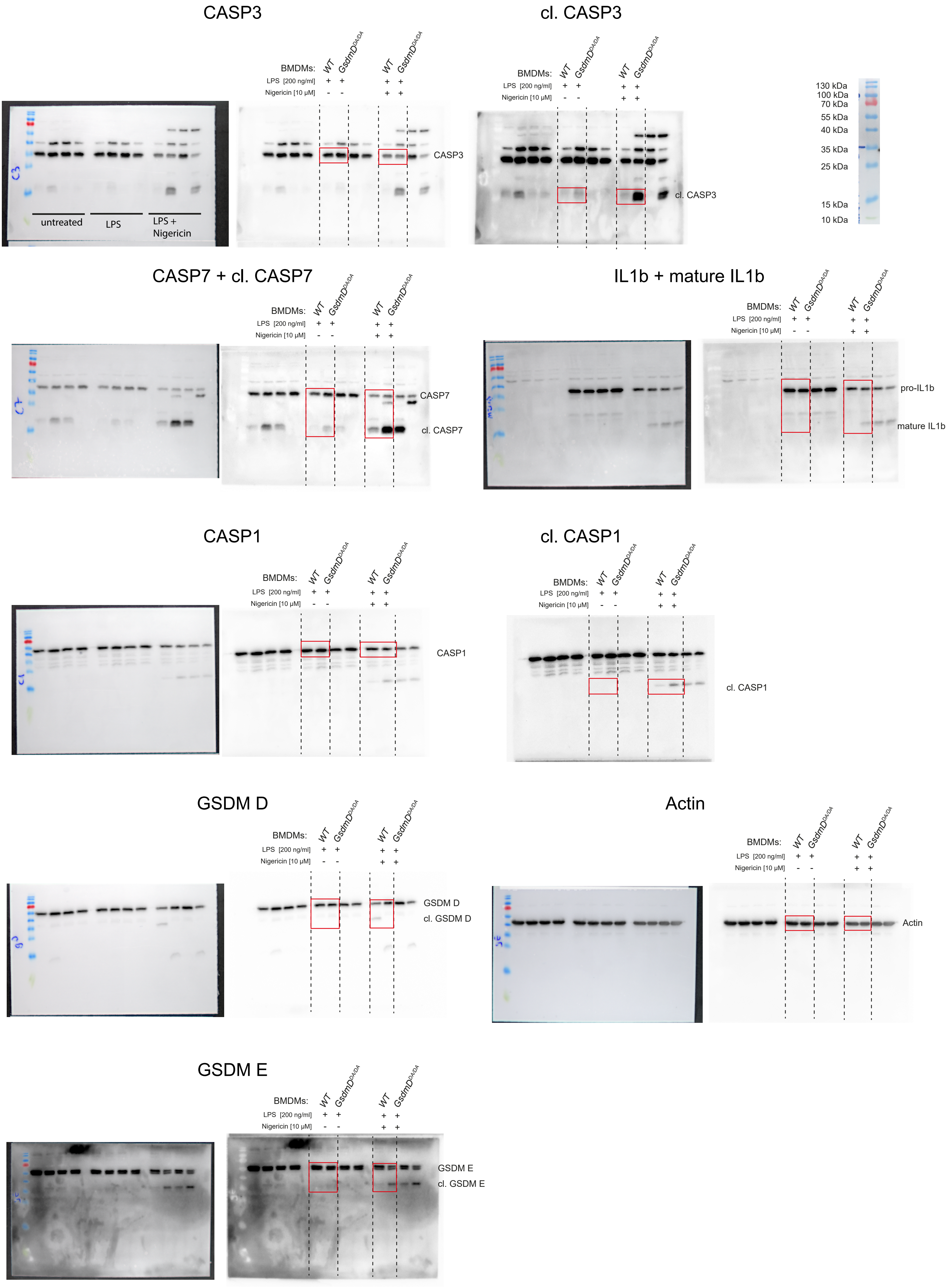
CASP3
cl. CASP3
BMDMs: WT GsdmDDA/DA WT GsdmDDA/DA
LPS [200 ng/ml] + + + +
Nigericin [10 µM] - - + +
untreated
LPS
LPS + Nigericin
CASP3
cl. CASP3
130 kDa
100 kDa
70 kDa
55 kDa
40 kDa
35 kDa
25 kDa
15 kDa
10 kDa
CASP7 + cl. CASP7
CASP7
cl. CASP7
IL1b + mature IL1b
pro-IL1b
mature IL1b
CASP1
cl. CASP1
GSDM D
cl. GSDM D
Actin
GSDM E
cl. GSDM E
